# Phosphorescent surface-anchored metal–organic frameworks for optical sensing of oxygen

**DOI:** 10.1039/d6tc02088g

**Published:** 2026-10-05

**Authors:** Theresa K. Mautz, Fabian Gasser, Roland Resel, Fernando G. Warchomicka, Christian Slugovc, Sergey M. Borisov

**Affiliations:** a Institute of Analytical Chemistry and Food Chemistry, Graz University of Technology 8010 Graz Austria sergey.borisov@tugraz.at; b Institute of Solid State Physics, Graz University of Technology 8010 Graz Austria; c Institute of Materials Science, Joining and Forming, Graz University of Technology 8010 Graz Austria; d Institute for Chemistry and Technology of Materials, Graz University of Technology 8010 Graz Austria slugovc@tugraz.at

## Abstract

Thin metal–organic framework (MOF) films, known as surface-anchored metal–organic frameworks (SURMOFs), offer convenient handling and a variety of new applications due to the direct immobilization of porous crystalline materials on various substrates. In this study, we demonstrate the feasibility of using luminescent SURMOFs for optical sensing of oxygen both in the gas phase and in organic solvents. Luminescent SURMOFs were prepared from indium(iii) chloride and platinum(ii) tetrakis(4-carboxyphenyl)porphyrin on pre-treated transparent substrates, such as rigid glass or flexible poly(ethylene terephthalate). These SURMOFs have an average thickness of ∼310 nm and exhibit room-temperature phosphorescence (*λ*_max_ = 645–676 nm). This phosphorescence is efficiently quenched by molecular oxygen in the gas phase (*I*_0_/*I*_air_ ∼50, Stern–Volmer constant *K*_SV_ ∼4–13 kPa^−1^). For the SURMOFs fabricated under similar conditions, the sensitivity does not correlate with the morphology (revealed by scanning electron microscopy) or degree of crystallinity (accessed by grazing-incidence wide angle X-ray scattering) of the porous films. Additionally, it has been demonstrated that the SURMOFs exhibit luminescence in toluene, which is quenched by oxygen (*I*_0_/*I*_air_ ∼ 2.5, *K*_SV_ ∼ 0.18 kPa^−1^). While optical oxygen sensing in organic solvents remains a significant challenge, the presented approach offers a viable solution, provided that future research can mitigate fast photodegradation of the SURMOFs under continuous irradiation.

## Abstract 2

Surface-anchored metal–organic frameworks (SURMOFs) grown on transparent glass and polymeric supports exhibit red luminescence that is quenched by molecular oxygen in both the gas phase and toluene.

## Graphical abstract

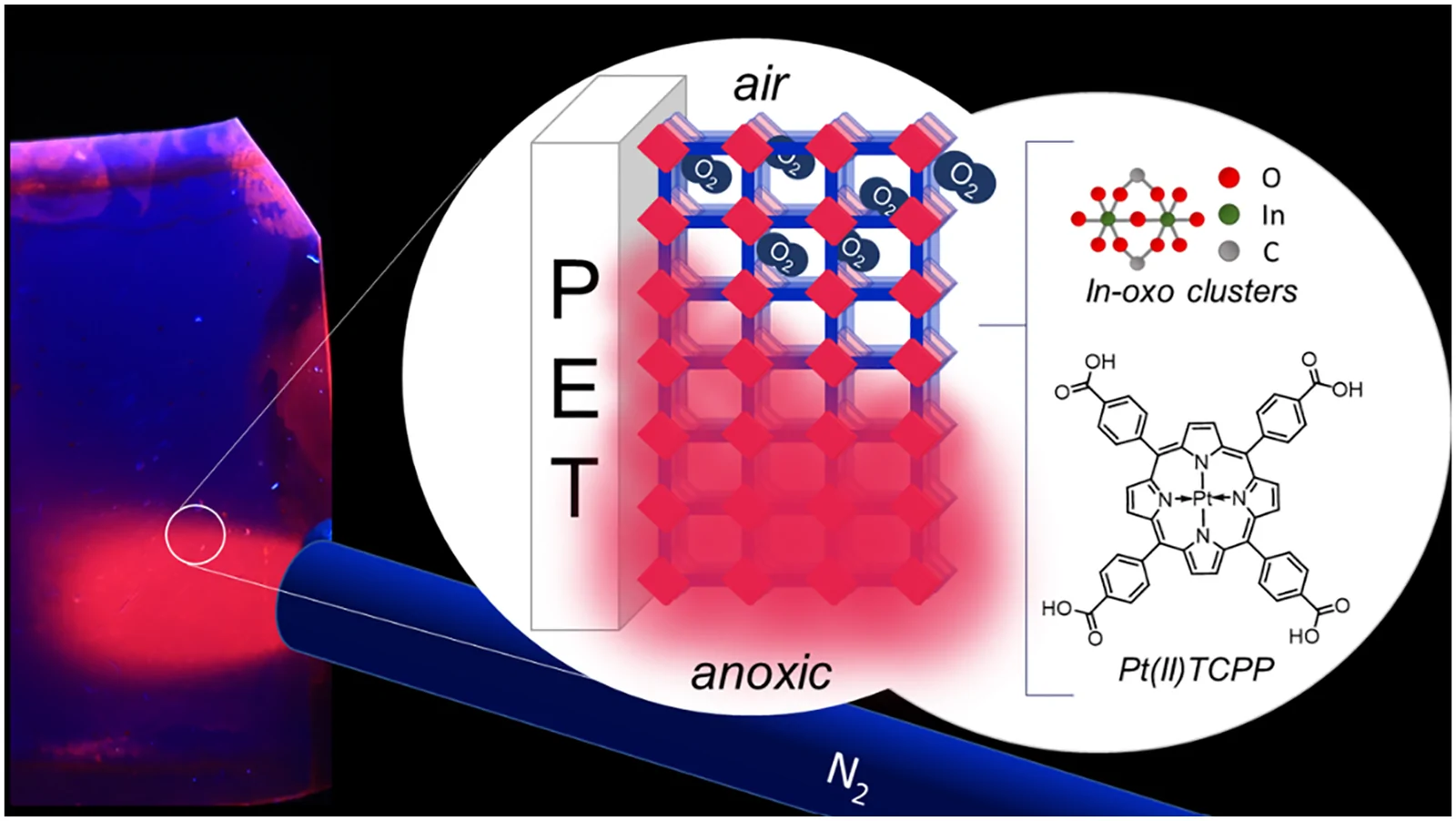

## 1. Introduction

Oxygen is one of the most important analytes monitored in industrial, biotechnological, environmental, and various other processes.^1–4^ Nowadays, oxygen quantification in gases and liquids under ambient and physiological conditions is often performed with optical sensors, based on the dynamic quenching of an indicator's luminescence by molecular oxygen.^5–8^ Typically, these materials rely on phosphorescent indicator dyes, immobilized in a gas-permeable matrix.^9^ Long luminescence lifetimes and efficient gas transport are crucial for effective oxygen quenching, resulting in highly sensitive probes.^5,9^ Among many different luminescent indicator families, platinum(ii) and palladium(ii) porphyrins are the most popular ones.^10^

State-of-the-art sensing materials are suitable for most common applications, but further improvements may be needed for the measurements in harsher environments, for instance higher temperatures or in the presence of chemicals that may affect the indicator or the immobilization matrix.^11,12^ Particularly, the measurement of oxygen in the presence of organic solvents challenges state-of-the-art materials due to swelling of the polymeric matrix and leaching of the indicator dye.^13,14^ The limited alternatives rely on fluorinated polymers,^12^ which raise environmental concerns such as long-term contamination of the food chain due to their non-biodegradability.^15^

Metal–organic frameworks (MOFs) are self-assembling crystalline materials composed of metal clusters and organic linkers.^16–18^ They offer a wide range of applications, including gas storage,^19,20^ catalysis,^21–23^ gas purification,^24^ and optical sensing.^25,26^ In particular, porphyrin-based MOFs are highly promising oxygen-sensing materials, as the indicator dye can be integrated into the framework as the organic linker while retaining its photophysical properties, enabling quenching by molecular oxygen.^27,28^ This results in a highly ordered structure with a defined environment, minimizing migration, leaching or agglomeration of the indicator. In particular, PCN-224, constructed from 5,10,15,20-tetrakis(4-carboxyphenyl)porphyrin (TCPP) and Zr–oxo-clusters as secondary building units (SBUs),^29,30^ has already shown promising luminescence quenching in the gas phase.^31^

Evidently, MOFs in the form of individual crystals have limited applications in sensing and require immobilization in a gas-permeable matrix that is coated onto a transparent solid planar support or an optical fiber, such as glass, polyethylene terephthalate (PET) or poly(methyl methacrylate).^27,32^ Finding a matrix that is resistant to harsher environments is particularly challenging;^12^ therefore, crystalline MOFs present no significant improvements over conventional sensor systems. Layer-by-layer (LbL) liquid-phase epitaxy (LPE) offers a route to a new subclass of rectangular materials: surface-anchored metal–organic frameworks (SURMOFs).^33–35^ In this process, MOF formation occurs directly on a functionalized surface by continuously exposing the substrate to solutions containing the building blocks. The LbL fabrication could result in highly homogeneous and oriented thin films, with controlled thickness.^34,36–39^ At this point, porphyrins are again popular building blocks and the intrinsic optical properties of these linkers make these SURMOFs interesting for various optical applications, such as energy storage^36^ or as highly sensitive photodetectors.^39^ The utilization of transparent substrates makes porphyrin-based SURMOFs^40^ particularly promising for sensing applications, overcoming the disadvantages of powder MOFs. While studies on bulk luminescent MOFs are extensive, the reports dedicated to luminescent SURMOFs, including porphyrin-based ones, are rarer.^41–43^ Moreover, oxygen-sensitive MOF thin films are often formed on a flat substrate in a procedure similar to that utilized for the preparation of powder MOFs rather than *via* LbL deposition typical for SURMOFs.^44^

In this work, the LbL-LPE method was utilized to prepare a series of phosphorescent SURMOFs based on Pt(ii)-porphyrins and In–oxo-clusters on rigid (glass) and flexible (PET) substrates. The porous layers composed of individual MOF “islands” allow fast gas diffusion. The SURMOFs show room-temperature phosphorescence originating from the Pt(ii) porphyrin, which is efficiently quenched by molecular oxygen in the gas phase. Additionally, the MOF films are found to be stable in toluene and show a luminescent response to oxygen, which paves the way for sensing of dissolved oxygen in organic solvents.

## 2. Experimental

### 2.1. Chemicals and materials

All chemicals were used without any further purification, unless otherwise mentioned. To obtain dry solvents, they were stored over molecular sieves (4 Å, Roth, https://www.carlroth.com) under inert conditions for at least three days. Platinum(ii) 5,10,15,20-tetrakis(4-carboxyphenyl)porphyrin (Pt(ii)TCPP) was purchased from Porphyrin Systems (https://www.porphyrin-systems.de). Bis(benzonitrile)dichloro-platinum(ii), sodium propionate, boron trifluoride diethyl etherate, pyrrole, iodomethane, anhydrous indium(iii) chloride, toluene and potassium carbonate were obtained from Sigma-Aldrich (https://www.sigmaaldrich.com). Methyl-3,5-difluoro-4-formylbenzoate and 3-(triethoxysilyl)propylsuccinic anhydride (3-ESPSA) were obtained from BLDPharm (https://www.bldpharm.com). 2,3-Dichloro-5,6-dicyano-1,4-benzoquinone (DDQ) was acquired from TCI (https://www.tcichemicals.com). Dichloromethane (DCM), ethanol, and potassium hydroxide (KOH) were obtained from Fisher Scientific (https://www.fishersci.at). Triethylamine, dimethyl sulfoxide (DMSO), and *N*,*N*-dimethylformamide (DMF) were acquired from Roth (https://www.carlroth.com). Methanol obtained from VWR (https://www.vwr.com) and hydrochloric acid obtained from Merck (https://www.merckmillipore.com) were used. 1*H*,1*H*,2*H*,2*H*-Perfluorooctyldimethylchlorosilane was obtained from ABCR (https://www.abcr.com). Deuterated dimethyl sulfoxide (DMSO-*d*_6_) for NMR analysis was purchased from Eurisotop (https://www.eurisotop.com). Nitrogen (purity 99.999%) and compressed air were obtained from Linde Gas (https://www.linde.at). Microscope glass slides (ISO 8037/1) were acquired from Epredia (https://www.epredia.com). Polyethylene terephthalate (PET) foils were purchased from Pütz (https://www.puetz-folien.com), and indium tin oxide (ITO) coated PET foils (100 mm × 200 mm) were obtained from Exp-Tech (https://www.exp-tech.de).

### 2.2. Instruments and methods

Flash chromatography was performed using a Biotage Selekt Chromatography system with Biotage Sfär silica-gel columns (https://www.biotage.com).

^1^H NMR spectra were recorded on a Varian INOVA 500 MHz spectrometer from Agilent Technologies (https://www.agilent.com) operating at 499.98 MHz.

Absorption spectra were acquired on an Agilent Cary 60 UV-VIS spectrophotometer from Agilent Technologies (https://www.agilent.com). Emission and excitation spectra were acquired on a Fluorolog-3 luminescence spectrometer from Horiba Scientific (https://www.horiba.com) equipped with an NIR sensitive R2658 photomultiplier from Hamamatsu (https://www.hamamatsu.com). The samples were placed in a homemade sample chamber exposed to a continuous gas flow of 200 mL min^−1^. The gas flow and the gas composition were controlled with two mass-flow controllers from Voegtlin (https://www.voegtlin.com) using compressed air and nitrogen (99.999% purity). To assess the spectral and oxygen-sensing properties of SURMOFs in toluene, the slides were fixed in a quartz cuvette (2 cm × 1 cm × 5.5 cm) filled with the solvent (see Fig. S39 for the set-up). The concentration of dissolved oxygen was adjusted by bubbling gas mixtures (flow rate of 40 mL min^−1^) through the solvents. In order to minimize evaporation, the gases were saturated with solvent vapors by bubbling through a glass flask containing 10 mL of the solvent, which was positioned before the measuring cuvette. The emission spectra were recorded using an OG 570 filter from Schott (https://www.schott.com) positioned in front of the emission monochromator to eliminate the second order grating artefact. In order to limit photodegradation during investigation of O_2_ sensing properties in the gas phase, a 10% transmission filter from Schott was positioned before the measurement chamber. Photostability of the SURMOFs was assessed with the same equipment by continuously illuminating the samples with a Xe lamp into the Soret band of the organic linker (400 nm, 7 nm slit, 10% transmission filter) and exposing the samples to an alternating atmosphere of nitrogen and air (10 min each). For the characterization of the MOF powder, a two-armed optical fiber bundle was utilized. The excitation light was guided to an NMR tube containing the MOF sample and the emission signal was guided back to the photodetector. The emission spectrum was recorded using an OG 570 filter from Schott under a nitrogen atmosphere (two mass-flow controllers from Voegtlin, with a gas flow of 200 mL min^−1^). Luminescence intensity at different oxygen concentrations was recorded using a 10% transmission filter from Schott.

Luminescence decays of SURMOFs and powder MOFs were recorded on the same instrument using a DeltaHub module and a 392 nm “SpectraLED” as an excitation source (both from Horiba). The heterogeneous microenvironment within the SURMOFs and the bulk MOF materials is held responsible for the deviation of the luminescence decays from the mono-exponential model. Therefore, the luminescence decays were processed with the DAS-6 Analysis software (Horiba) using a bi-exponential decay model for fitting, and average decay times were calculated.

Scanning electron microscopy (SEM) images were obtained at the field emission SEM TESCAN Mira 3 (https://www.tescan.com, Brno, CZ) using secondary and in-beam secondary electrons for characterization, with an acceleration voltage of 5 kV and a beam size of about 4.8 nm. For sample preparation, 17 nm of copper was deposited on the samples by thermal evaporation at a base pressure of 8 × 10^−6^ mbar with a deposition rate of 0.3 Å s^−1^.

Grazing-incidence wide-angle X-ray scattering measurements (GIWAXS) of SURMOF samples were performed on the XRD-1 beamline, Elettra Sincrotrone (Trieste, Italy), using X-rays with a wavelength of 1.4 Å and an incident angle of *α*_i_ = 0.1°. The data were collected on a Dectris Pilatus 2 M detector at a nominal sample–detector distance of 20 cm. For calibration, a LaB_6_ standard was used. The measured data were transformed into reciprocal space and azimuthally integrated using software GIDVis.^45^

Atomic force microscopy images were acquired on a Tosca 400 from Anton Paar (https://www.anton-paar.com) assembled with a vibrational reducing hood. The measurement was performed in the tapping mode, using the Arrow-Silicon SPM-Sensor from Anton Paar (thickness: 4.6 µm; length: 160 µm; width: 45 µm). An area of 25 µm × 25 µm or 20 µm × 20 µm was measured at a scan rate of 0.6 s per line. The resolution was set to 500 and the gain to 600.

Thermogravimetric analysis (TGA) was carried out on a TGA 8000 from PerkinElmer (https://www.perkinelmer.com) under a nitrogen atmosphere (gas flow: 20 mL min^−1^). The temperature range was set from 30 °C to 600 °C at a heating rate of 10 °C min^−1^. The temperature was held for 1 min at 30 °C and 600 °C.

### 2.3. Synthesis

#### 2.3.1. Synthesis of organic linkers

Platinum(ii) 5,10,15,20-tetrakis(2,6-difluoro-4-carboxyphenyl)porphyrin (*meso*-8F-Pt(ii)TCPP) was synthesized as reported previously^46^ from the metal-free porphyrin obtained *via* a Lindsey method using methyl-3,5-difluoro-4-formylbenzoate and pyrrole as educts.

#### 2.3.2. Glass substrate modification

Microscope glass slides (ISO 8037/1, from Epredia) were modified on both sides using two different silanization techniques. Prior to the silane treatment, the microscopy-grade glass slides were thoroughly cleaned with acetone and ethanol and dried under a nitrogen stream. The back side of the microscope glass slide was modified *via* drop-casting silanization using 1*H*,1*H*,2*H*,2*H*-perfluorooctyldimethylchlorosilane (150 µL per two “sandwiched” slides), followed by a cleaning step with EtOH. The COOH-groups on the front side were introduced using an established procedure.^47^ The glass slides were placed in a 100 mL round bottom flask together with an open container of 3-ESPSA on the bottom. Modification of the surface was performed under vacuum and heating to 65 °C for 24 h, followed by submerging in deionized water for 1.5 h and drying under a nitrogen stream.

#### 2.3.3. Modification of PET foils

PET foils were modified according to the literature.^48^ PET foils were cleaned with acetone and ethanol, dried under a nitrogen stream, immersed in 2 M NaOH and heated to 70 °C for 30 min. Afterwards the foils were rinsed with deionized water, immersed in acetic acid at 70 °C for 30 min and rinsed again with deionized water.

#### 2.3.4. Fabrication of SURMOFs

The SURMOFs were fabricated by the established LbL liquid-phase epitaxial dipping procedure,^34,49^ with the help of an autosampler ASX-1400 from CETAC (https://www.cetrac.com) equipped with a self-built sample holder. Briefly, the functionalized substrate was alternately immersed in the solutions of the organic linker (Pt(ii)TCPP or *meso*-8F-Pt(ii)TCPP, various concentrations, Table 1) and the metal node solutions (1.1 mM InCl_3_) with washing with a pure solvent after each immersion. After the deposition, the slides were rinsed with the solvent used for the MOF fabrication, the film from the hydrophobic side of the slide was removed by wiping with a paper towel soaked with ethanol and the slides were immersed in ethanol and methanol for several hours to remove DMF and the uncoordinated linkers from the pores. The solvent was exchanged several times and the slides were stored in ethanol until characterization. A detailed description of the fabrication is given in the SI (S2). The prepared SURMOFs are further referred to according to their fabrication parameters (Table 1). For example, SURMOF-DMF-38c indicates that the SURMOF was prepared from a DMF solution with a linker concentration of 38 µM and it is the third sample in triplicate.

**Table 1:** Experimental conditions for preparation of the SURMOFs

	Organic linker	Organic linker Conc. (µM)	Solvent	Substrate
SURMOF-DMF-38X^a^	Pt(ii)TCPP	38	DMF	Glass
SURMOF-DMF-25	Pt(ii)TCPP	25	DMF	Glass
SURMOF-DMF-50	Pt(ii)TCPP	50	DMF	Glass
SURMOF-DMF-8F	*meso*-8F-Pt(ii)TCPP	38	DMF	Glass
SURMOF-EtOH-38X^a^	Pt(ii)TCPP	38	EtOH	Glass
SURMOF-ITO	Pt(ii)TCPP	38	DMF	PET-ITO
SURMOF-PET	Pt(ii)TCPP	38	DMF	PET-COOH

a SURMOF-DMF-38X and SURMOF-EtOH-38X were fabricated multiple times and are labelled as “-a”, “-b”, “-c”, “-d” and “-e”.

## 3. Results and discussion

### 3.1. Synthesis of SURMOFs

All SURMOFs are grown using LbL LPE, one of the most frequently used techniques for SURMOF fabrication.^36^ In brief, functionalized substrates are alternately immersed in solutions containing individual reactants, with washing steps in between (Fig. 1c). For the SURMOFs composed of TCPP and In–oxo-clusters, the essential parameters (substrates, concentration of the building blocks and substrate exposure time) vary considerably^39,40,50^ (Chapter 6, SI). Therefore, we tested different linker concentrations (25, 38 and 50 mM) and two solvents (DMF and EtOH) to evaluate their influence on the sensitivity to molecular oxygen and the reproducibility of the manufacturing procedure. Pt(ii)TCPP was used as the organic linker in most of the experiments. We additionally tested a related linker, a recently reported partly fluorinated tetra-carboxyphenyl-porphyrin *meso*-8F-Pt(ii)TCPP (Table 1, Fig. 1).^46^

**Fig. 1:**
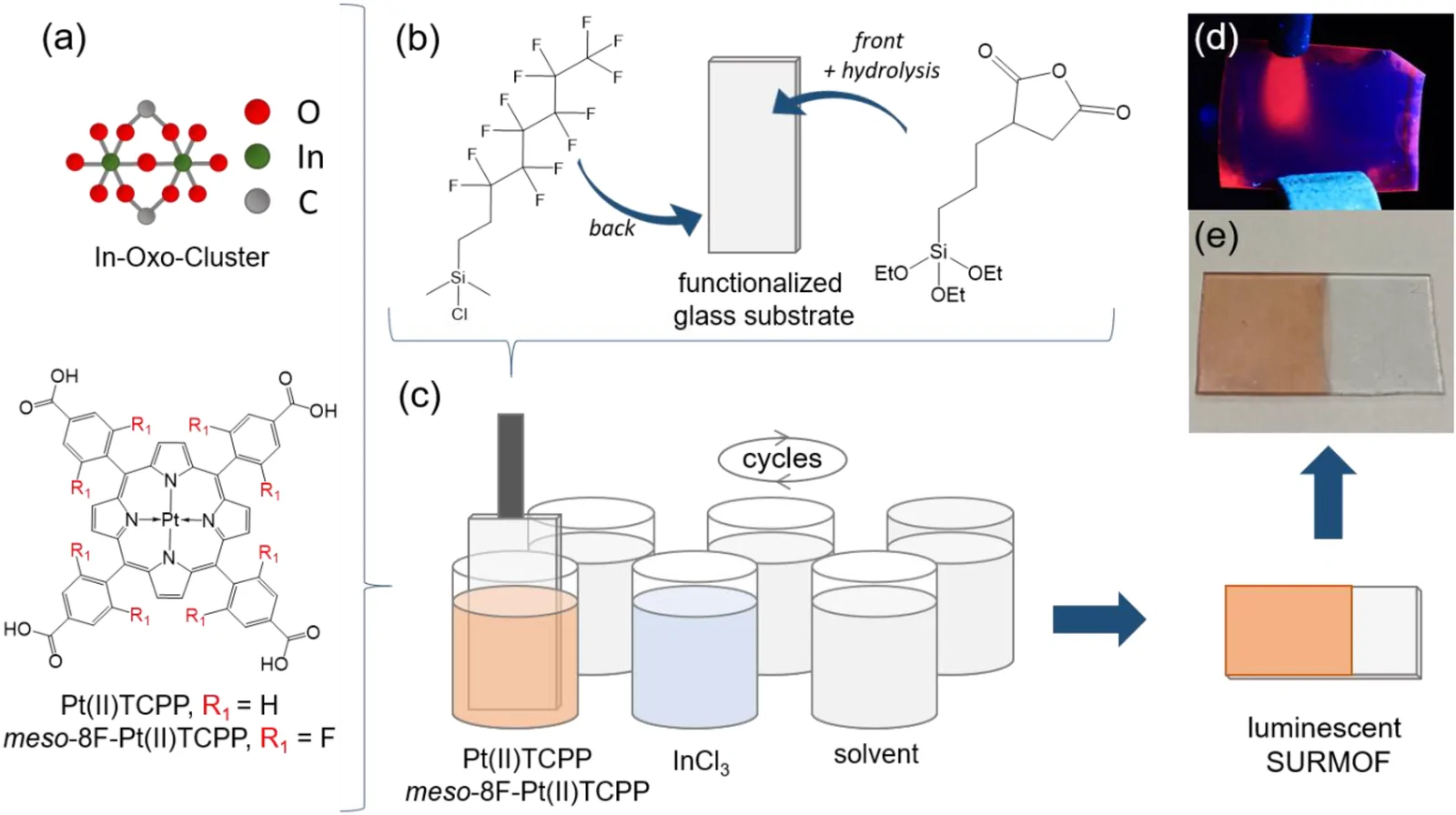
Schematic illustration of the synthesis of Pt(ii)TCPP In–oxo-cluster based SURMOF *via* LbL LPE: (a) Building blocks. (b) Functionalization of the glass substrate by immobilizing hydrophobic 1*H*,1*H*,2*H*,2*H*-perfluorooctyldimethylchlorosilane on the back side and 3-(triethoxysilyl)propylsuccinic anhydride on the front side. Hydrolysis leads to the cleavage of succinic anhydride, resulting in two –COOH groups. (c) Dipping set-up using an autosampler: metal-node solution, organic linker solution and solvent for washing steps. Photographic images of SURMOF-EtOH-38a (e) and SURMOF-PET (d) under ambient light and UV illumination, respectively. (d) Foil in air under UV illumination with nitrogen blown onto the foil from a plastic tube.

Unlike the literature, which mostly uses silicon substrates, we selected transparent substrates (glass and polyethylene terephthalate, or PET) to enable optical readout from the substrate side. Functionalization of the glass substrates (in the form of microscopic slides) can be conveniently performed using established silanization procedures. First, the back surface was modified with a hydrophobic silane (1*H*,1*H*,2*H*,2*H*-perfluorooctyldimethylchlorosilane) to block the surface for the deposition of SURMOF (Fig. 1b). Then, the front side was modified with –COOH groups^47^ to enhance the mechanical stability of the MOF films due to anchoring of metal–oxo-clusters on the glass surface. This modification was done *via* deposition of a self-assembling monolayer (SAM)^34^ of 3-(triethoxysilyl)propylsuccinic anhydride and subsequent hydrolysis to generate –COOH groups.^47^ In the case of the flexible PET substrate the –COOH groups were generated *via* alkali treatment at elevated temperature, resulting in partial hydrolysis of the polymer on the surface.^48^

SURMOFs are commonly grown from ethanolic solutions of the building blocks^39,51^ whereas DMF is a common solvent for the synthesis of MOFs in the powder form. Literature reports on the preparation of SURMOFs from DMF are limited;^52,53^ however, we expected less variation due to the comparably high boiling point of the solvent. In fact, we found it challenging to prevent partial evaporation of ethanol during the lengthy preparation process, which led to poorly controllable changes in reagent concentration during deposition.

Although the SURMOF film becomes visible after about 24 hours, the deposition was performed for a total of three days to ensure sufficient film thickness for optical applications. The resulting film covers the full area and shows the characteristic orange color similar to that of the dissolved dye (Fig. 1e).

Since the remaining DMF accelerates the photodegradation process of porphyrin-based powder MOFs,^45^ removing the solvent from the pores of the SURMOFs after the fabrication is crucial to prevent rapid degradation. Notably, drying of the SURMOF under vacuum at an elevated temperature leads to a strong decrease in luminescence intensity and a loss of sensitivity (Chapter 3.8, SI). Therefore, in order to avoid the above changes, the activation was performed exclusively by solvent exchange at room temperature. To achieve this, the SURMOF-coated substrates were immersed several times in ethanol. The procedure was then repeated with methanol.

### 3.2. Structural characterization of SURMOFs

SEM images (Fig. 2 and Fig. S12–S17 of the SI) reveal very different surface morphologies of the samples, which may indicate varying degrees of crystallinity. All the SURMOFs feature small separate units that grow together and cover the full surface. This behavior is similar to other SURMOFs reported in the literature and the closely related SURMOF based on In–oxo-clusters and metal-free TCPP.^39,52,54–57^ The average film thicknesses measured by AFM (Fig. S4–S6 and S9–S11, SI) revealed a homogeneous coating with an average thickness of 310 nm for SURMOF-DMF-38a, but the presence of agglomerates and significant differences in height for SURMOF-EtOH-38a (average film thickness of 431 nm). Therefore, based on the thickness of the film and the size of the units visible in SEM images, it can be assumed that the film is composed of several layers of separate MOF clusters. Additional macropores between the units, besides the micropores of the SURMOF itself, may favor diffusion of gases and other species. Comparison of the replicates (SURMOF-DMF-38a and SURMOF-DMF-38b), which were fabricated using the same ratio of the building blocks and identical conditions, indicates that the control of the surface morphology is challenging (Fig. 2).

**Fig. 2:**
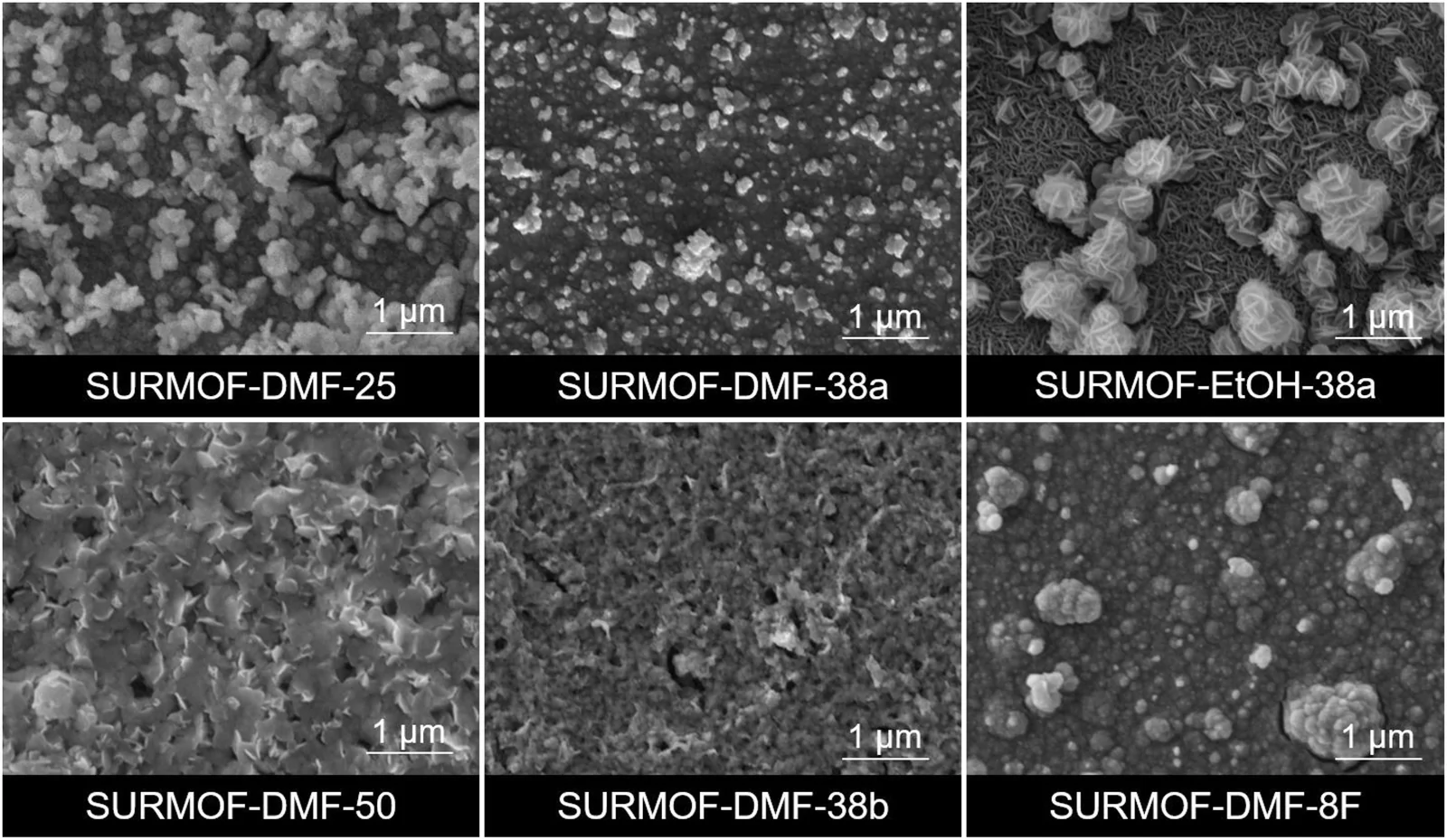
Scanning electron microscopy images of SURMOFs based on In–oxo-clusters and Pt(ii)TCPP.

The films were then investigated using grazing-incidence wide-angle X-ray scattering (GIWAXS). The obtained scattering patterns (Fig. 3 and Fig. S18–S26, SI) feature diffraction peaks at 0.54 A^−1^ and 1.08 A^−1^ and further weak peaks become visible at 0.75 A^−1^ and 0.89 A^−1^ indicating that all the SURMOFs have the same crystal structure.

**Fig. 3:**
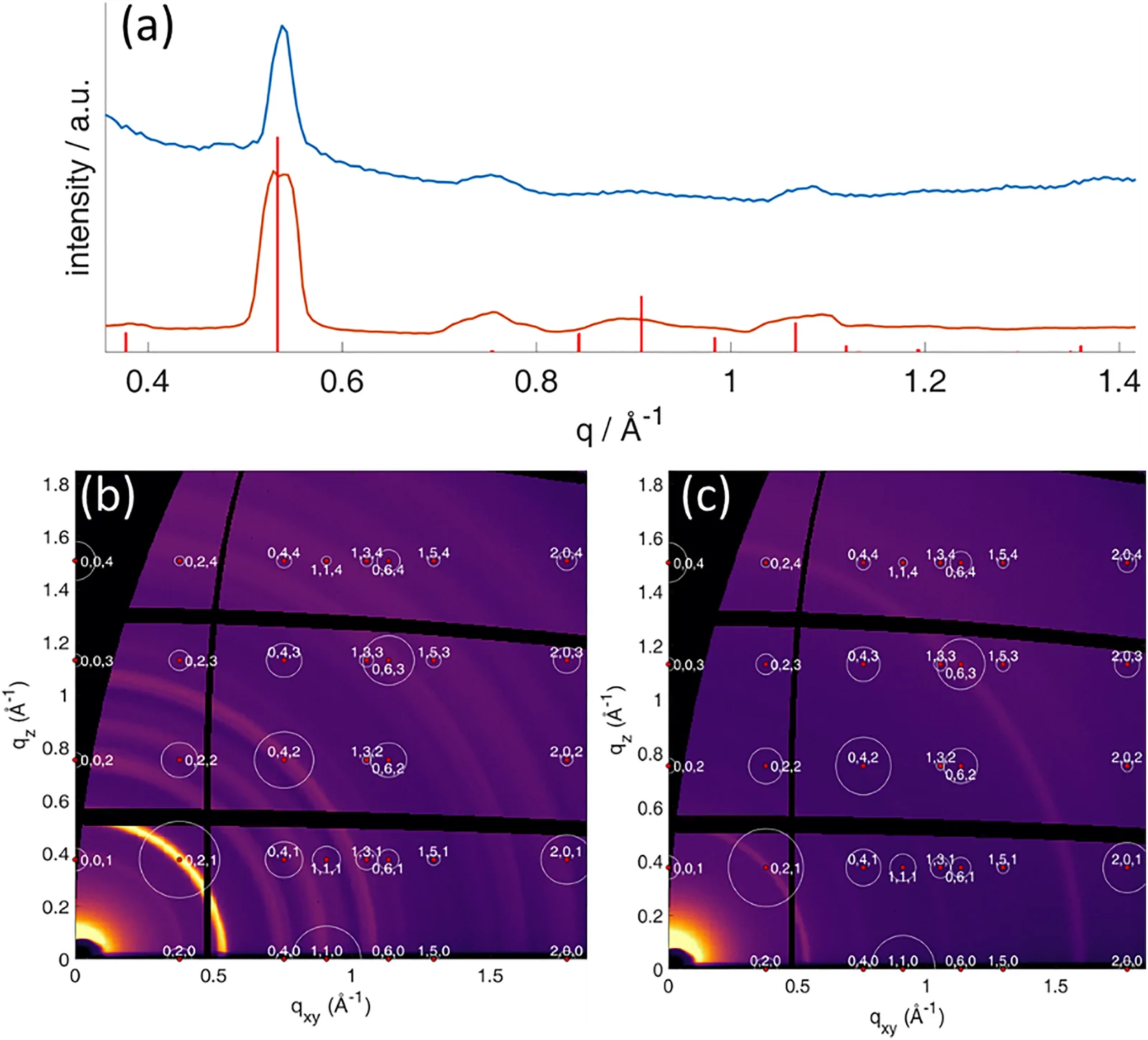
GIWAXS data from selected SURMOFs. (a) Diffraction pattern obtained by azimuthal integration (along Debye–Scherrer rings) based on the 2D-diffraction pattern of SURMOF-DMF-38a and SURMOF-EtOH-38a. GIWAXS patterns of SURMOF-EtOH-38a (b) and SURMOF-DMF-38a (c). The calculated peak positions and peak intensities are based on the structure of ERALAN,^58^ and the result is given in the case of (a) by vertical bars and in the case of (b) and (c) by circles with defined centers and areas denoting peak positions and peak intensities.

To identify the crystal structure, we selected MOFs from the Cambridge Structural Database with a comparable framework structure prepared from different metal-TCPP linkers and indium(iii) nitrate. An isomorphous series is found with an identical space group (*Cmmm*), slightly varying lattice constants and unit cell volumes between 3923 Å^3^ and 3981 Å^3^: ERALIV (based on TCPP), ERALOB (Co(ii)TCPP), ERALER (Ni(ii)TCPP), ERALUH (Cu(ii)TCPP) and ERALAN (with In(iii)).^58^ These MOFs exhibit similar diffraction patterns with nearly identical peak positions, but varying peak intensities due to the different metal atoms coordinated at the porphyrin core. To compare the experimental data with a calculated diffraction pattern, we selected ERALAN. Excellent agreement in peak position is observed, but clear differences are present in the peak intensities. Please note that the peak located at *q* = 0.75 Å^−3^ can be indexed by peaks with Laue indices 002 and/or 040. The differences in peak intensities are a consequence of the use of Pt-porphyrin and fluorinated organic linkers. Using the partly fluorinated organic linker does not affect the crystal structure formed during the LbL fabrication, as SURMOF-DMF-8F exhibits the same position of the diffraction peaks (with variations in intensities).

Different diffraction patterns of our samples (Fig. S18, SI) indicate different degrees of crystallinity of the SURMOFs. SURMOF-DMF-38a and SURMOF-DMF-38b show the same degree of crystallinity despite their very different surface morphology as seen from SEM images (Fig. 2). The reason is currently unknown and might originate from poorly controllable factors, for instance daily variations in temperature or humidity in the laboratory during the deposition process. Additionally, SEM images show only a small part of the sample, while the GIWAXS results present the average value recorded over the whole film. Nevertheless, the diffraction patterns show that despite these differences, all the SURMOFs are characterized by the same crystal structure.

Using GIWAXS, it was determined that SURMOFs show a similar structure to the powder MOF, consisting of the porphyrin based organic linkers and In–oxo-clusters. Accordingly, it can be assumed that the BET (Brunauer–Emmett–Teller) surface areas of SURMOF (for which experimental estimation is unfeasible due to the very small amount of coating, ≪ 1 mg) are comparable to those of the bulk material.

The literature values of the BET surface area of about 1200 m^2^ g^−1^ are rather high, ensuring fast gas diffusion in the MOF. The literature values for metalated organic linkers (800–1100 m^2^ g^−1)^ tend to be lower, but are still sufficiently high to allow good oxygen diffusion.^58^

The thermogravimetric analysis (Fig. S50 of the SI) of Pt(ii)TCPP/In–oxo-cluster powder MOF reveals the thermal stability of the material. Approximately 7% of the weight is lost between 150 °C and 250 °C, which can be attributed to the removal of residual solvent (mainly DMF) and water molecules entrapped in the pores. The major mass losses at higher temperatures (350–550 °C) are attributed to the decomposition of the organic ligand and decarboxylation reactions. These changes are typical for MOFs based on In–oxo-clusters and porphyrin based organic linkers.^58^

### 3.3. Photophysical properties and oxygen sensitivity of SURMOFs in the gas phase

Similar to the parent porphyrin linkers, the SURMOFs are colored (Fig. 1e) due to efficient light absorption in the green part of the electromagnetic spectrum. As expected, porphyrinic SURMOFs show phosphorescence in the red to far-red part of the electromagnetic spectrum. The emission spectra closely resemble the emission spectrum of the dyes in solution, with the most intense band at 650–670 nm originating from the 0,0 transition (Fig. 4 and Fig. S27 of SI). The excitation spectra show characteristic Q-bands: the most intense band with *λ*_max_ ∼ 505 nm (0,1 transition) and a shoulder at ∼535 nm corresponding to the 0,0 transition (Fig. 4, Fig. S29–S32 of the SI). Although the general form and approximate position of the excitation and emission bands are the same for all the SURMOFs, the spectra show some variation depending on the ratio of the building blocks and the preparation method (Table 2). This may originate from different crystallinity of the samples, distortion due to the inner-filter effect (different thicknesses for various samples) or the effect of light scattering due to different turbidity of the films. Notably, the triplicates obtained from DMF solutions show much higher consistency in their absorption and excitation spectra than the samples made from EtOH series solutions (Table 2).

**Fig. 4:**
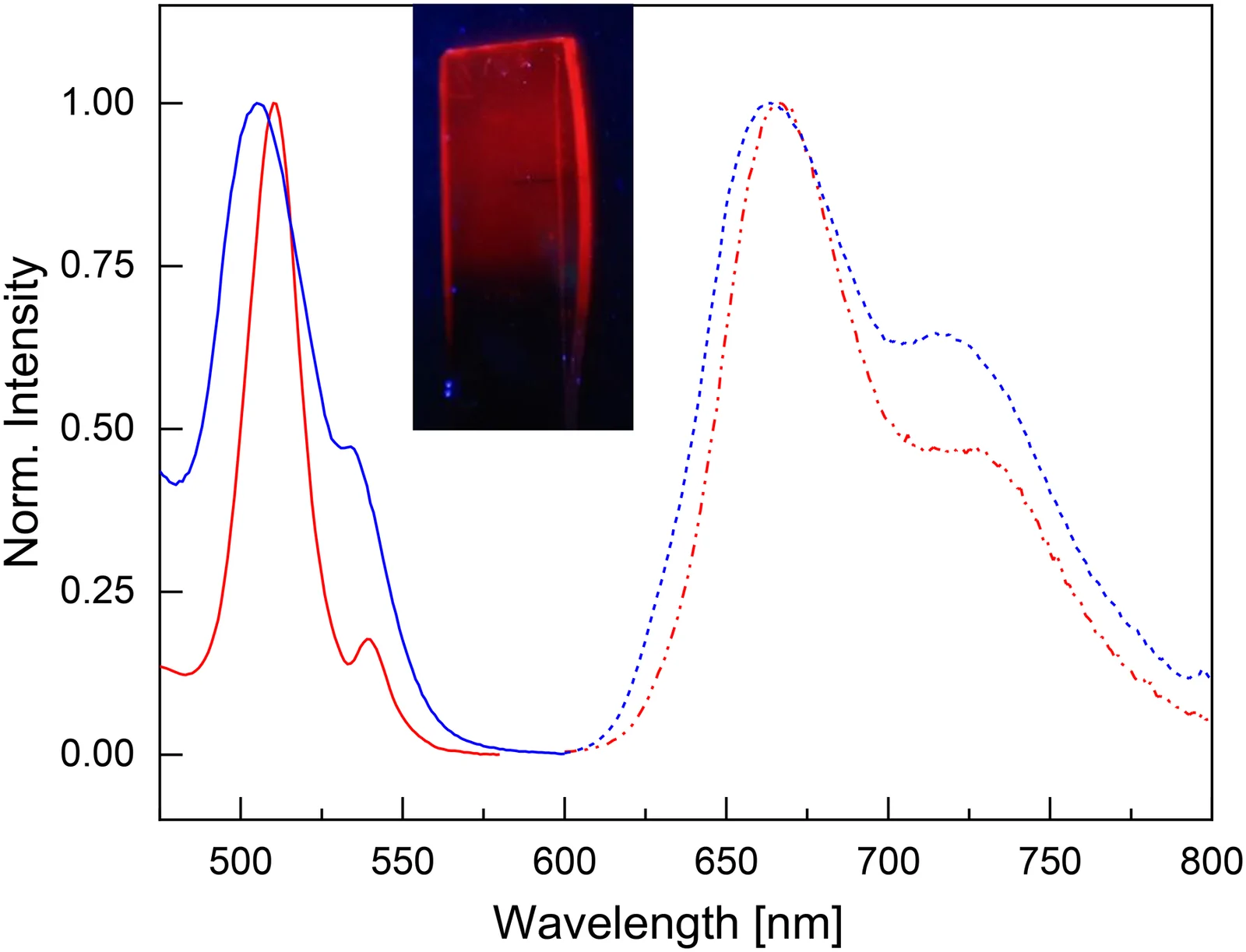
Spectral properties of Pt(ii)TCPP in solution and in SURMOF-DMF-38a. Red lines: absorption (solid line) and emission (dotted line, *λ*_exc_ = 400 nm, anoxic conditions) spectra of Pt(ii)TCPP in DMF; blue lines: excitation (solid line *λ*_em_ = 660 nm) and emission (dotted line, *λ*_exc_ = 400 nm) of the SURMOF under anoxic conditions. The photographic image shows the luminescence of the coating under UV excitation and nitrogen jet.

**Table 2:** Spectral and oxygen sensing properties of the porphyrinic SURMOFs

	*λ*_max_ Ex, Q-band (nm)	*λ*_max_ Em (nm)	*τ*_0_ (µs)	*I*_0_/*I* ∼ 20.9 kPa	*K*_SV1_ a (kPa^−1^)	*f*
SURMOF-DMF-25	509, 548	677, 729	41	44 ± 4	4.6	0.99
SURMOF-DMF-50	498, 540	685, 728	25	18 ± 0.9	1.9	0.97
SURMOF-DMF-38a	506, 535	665, 717	42	57 ± 8	11.4	0.99
SURMOF-DMF-38b	506, 534	665, 717	43	56 ± 8	11.3	0.99
SURMOF-DMF-38c	507, 536	659, 717	31	49 ± 8	7.2	0.99
SURMOF-EtOH-38a	503, 535	647, 712	26	64 ± 4	12.6	0.99
SURMOF-EtOH-38b	508, 534	657, 709	40	45 ± 2	12.4	0.98
SURMOF-EtOH-38c	503, 534	675, 733	32	23 ± 0.4	3.7	0.96
SURMOF-DMF-8F	508, 540	652, 710	15	40 ± 6	6.9	0.98

a *K*_SV2_ was set to 0.

The luminescence of the SURMOFs was found to be quenchable by molecular oxygen (Fig. 5, Fig. S37 and Video S1 of the SI). This is in line with previous reports on the crystalline MOFs composed of zirconium–oxo-clusters and Pt(ii)TCPP or *meso*-8F-Pt(ii)TCPP.^27,46,59^ Stern–Volmer plots (Fig. 5 and Fig. S37 of the SI) confirm the very efficient luminescence quenching. Defects in the structure or blocked channels may hinder oxygen diffusion and result in different oxygen sensitivity. To account for that limitation, the so called two-site model was used to describe the quenching behavior of such materials:^60^1
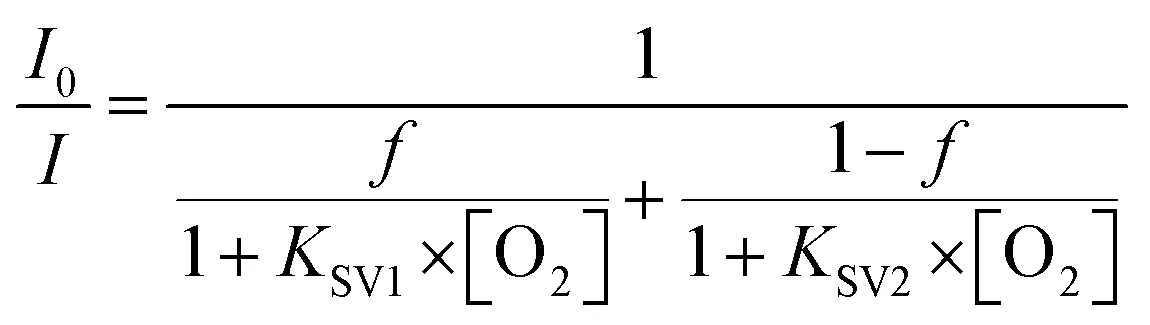


**Fig. 5:**
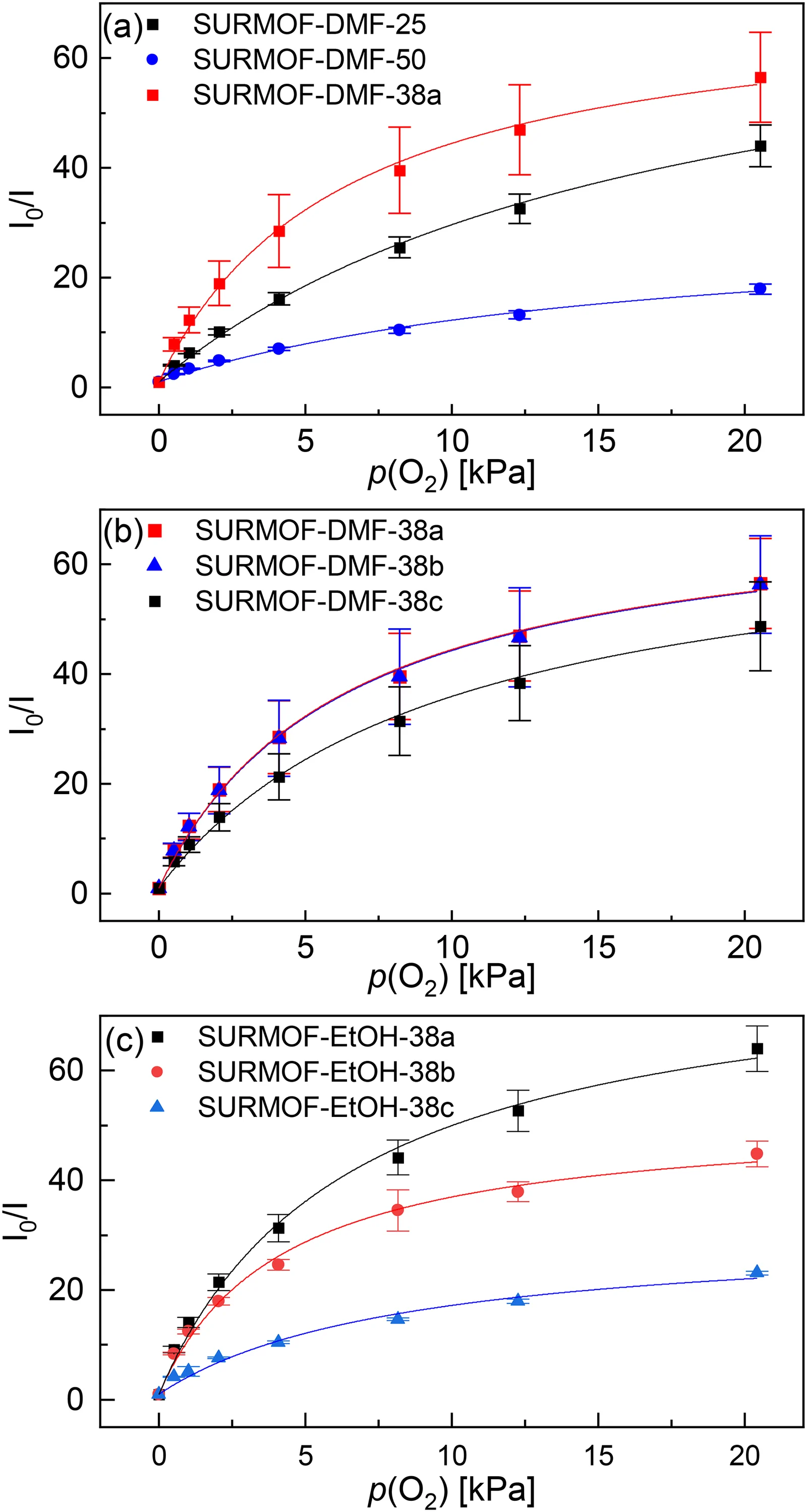
Stern–Volmer plots (fitted according to eqn (1)) of SURMOFs based on Pt(ii)TCPP. The intensity was measured at the maximum of the emission spectrum (*λ*_ex_ = 400 nm).

The equation assumes the location of the indicator in two environments, characterized by two Stern–Volmer constants (*K*_SV1_ and *K*_SV2_) and the distribution coefficient *f* that corresponds to the proportion of the dye in the first environment.

In the Pt(ii)TCPP-based DMF series, the highest sensitivity is observed for the SURMOFs obtained from a ∼30 : 1 molar ratio of indium to porphyrin precursors, whereas higher and lower ratios result in less sensitive MOFs (Fig. 5a). The replicates prepared from DMF solutions show similar response to oxygen (Fig. 5b) despite differences in intensity in the GIWAXS measurements and in morphology, indicating varying degrees of crystallinity. This suggests that sensitivity is not directly related to the crystallinity but rather to the fabrication parameters, which considerably simplifies handling during the manufacturing process. Nevertheless, it is possible that other factors (for example, variations in humidity) also affect the morphology and sensing characteristics of the resulting coating. For instance, the sensitivity of SURMOF-DMF-38c is slightly lower, which correlates with the shorter decay time of the Pt(ii) porphyrin in this material (Table 2).

Not surprisingly, spectral properties of the resulting SURMOF using *meso*-8F-Pt(ii)TCPP as the organic linker were found to be similar to those obtained for the Pt(ii)TCPP-based materials (Fig. S37 of the SI). The efficiency of oxygen quenching (*K*_SV1_ = 6.9 kPa^−1^) was found to be lower than that of the non-fluorinated SURMOF-DMF-38X and a significantly shorter phosphorescence lifetime of the *meso*-8F-Pt(ii)TCPP-based SURMOF (15.5 µs) was recorded.

In contrast, the SURMOFs deposited from ethanolic solutions show much greater variation in sensitivity than those prepared from DMF solutions (Fig. 5c). The poor reproducibility is likely caused by uncontrollable evaporation of ethanol during the deposition, affecting luminescence decay times and the oxygen-sensing properties. Additionally, the higher reproducibility observed for SURMOF-DMF-38X might be attributed to the chemical properties of the respective solvents. DMF provides better precursor solubility and contains traces of decomposition products that may act as modulators. The batch-to-batch variations found for SURMOF fabrication from EtOH might originate from the lower precursor solubility and subsequent aggregation, leading to limited desorption at the substrate interfaces.^34,61,62^

Interestingly, despite strong deviation between the individual samples, the SURMOFs grown from ethanol showed significantly less drift during the measurements. The drift observed for the new SURMOFs is undoubtedly a strong limitation for potential gas sensing applications. This drift is attributed to photobleaching during illumination of the sample (Fig. S41A and B illustrating accelerated bleaching under continuous irradiation).

Similar to the porphyrinic MOFs based on zirconium–oxo-clusters,^46^ the photodegradation of the SURMOFs might involve entrapped traces of solvents such as DMF that are used during the MOF deposition. Here it can be concluded that deposition from solvents with high boiling points appears to lead to coatings with more reproducible characteristics but bears the risk of entrapment of these solvents in the pores and thus accelerates photobleaching. Thus, investigating other solvents for SURMOF preparation (bp. » 78 °C) may lead to more photostable SURMOFs.

It is also interesting to compare the oxygen-sensing properties of the SURMOFs with those of the bulk crystals prepared *via* the solvothermal method (Fig. S48 and S49 of the SI). The excitation spectrum of this material is similar to that of the SURMOF, but in the emission spectrum, the intensity of the second band, associated with the 0,1 transition (*λ*_max_ ∼ 750 nm), is significantly higher than the intensity of the 0,0 band (*λ*_max_ ∼ 670 nm, Fig. S45 of the SI). The phosphorescence decay time of the Pt(ii) porphyrin in the powder MOF (39 µs) is similar to that of the SURMOFs, and it also shows comparable *K*_SV1_ values (10 kPa^−1^).

Thus, the sensitivity of the MOFs based on Pt(ii)TCPP and In–oxo clusters (both in the form of SURMOFs and powder MOFs) is significantly lower than that of the powder MOFs that rely on the same linker and Zr–oxo clusters, for example, Pt(ii)PCN-222 (*K*_SV1_ = 30 kPa^−1^) and (Pt(ii)PCN-224; *K*_SV1_ = 73 kPa^−1^).^27^ The lower sensitivity could be attributed to slower oxygen diffusion through the framework to the indicator linkers and reduced quenching in some areas of the material. Notably, the Stern–Volmer plot of the Pt(ii)TCPP and In–oxo-cluster powder MOF deviates strongly from the linear behavior, indicating a high contribution of the non-quenchable component. The slower diffusion might be explained by the moderate BET surface area assumed for the SURMOFs based on the data reported for the powder MOFs based on In–oxo-clusters and the free base TCPP porphyrin (1200 m^2^ g^−1^). For comparison, the values reported for the PCN-224 MOF are much higher (up to 2700 m^2^ g^−1^).^58,63^ In addition to the oxygen diffusion rates within the framework, the microenvironments surrounding the porphyrins in the SURMOFs may influence the oxygen sensitivity. The environment might promote non-radiative de-excitation pathways and therefore affect the overall sensing performance.

### 3.4. SURMOFs on flexible supports

Poly(ethylene terephthalate) is a flexible transparent substrate utilized in optical sensing technology that allows convenient processing into a desired shape (*e.g.*, cutting or punching into small round sensor spots). In order to generate carboxylic groups for anchoring the SURMOFs onto the PET surface, it was chemically treated. Commercially available indium tin oxide (ITO)-coated PET foils were also investigated since we expected that the ITO layer promotes nucleation and SURMOF formation due to chemical similarity of the ITO layer and the nodes (In–oxo-clusters). The same synthesis conditions as those used for SURMOF-DMF-38X were selected, as they achieved the best luminescence lifetimes and the highest reproducibility.

Indeed, similarly to glass substrates, colored luminescent coatings with good mechanical stability were obtained (Fig. 6a). The emission and excitation spectra of the SURMOF prepared on the chemically treated PET substrate (SURMOF-PET) show high similarity to those prepared on glass, but the coating on the ITO layer (SURMOF-ITO) is characterized by more defined 0,0 and 0,1 transitions in the emission spectra. The luminescence lifetimes were generally similar for both samples (*τ* = 38 and 34 µs, for SURMOF-PET and SURMOF-ITO, respectively). The luminescence of SURMOF-PET is efficiently quenched by molecular oxygen in the gas phase (Fig. 6b), but the sensitivity is significantly lower (*K*_SV1_ = 1.77 kPa^−1^) than for the materials prepared on glass. The Stern–Volmer plot shown by SURMOF-ITO is rather different in form from the one shown by SURMOF-PET, with a lower Stern–Volmer constant of *K*_SV1_ ∼ 0.8 kPa^−1^ (Fig. 6b), and the material also shows particularly fast photodegradation (Fig. S38C of the SI). The difference in the shape of the Stern–Volmer plots and photodegradation speed might be associated with a different amount of residual DMF that might diffuse into the polymeric support during the fabrication process and remain there even after the solvent exchange. The remaining DMF molecules might migrate from the (ITO coated) PET foil into the framework and cause faster degradation during the measurements.

**Fig. 6:**
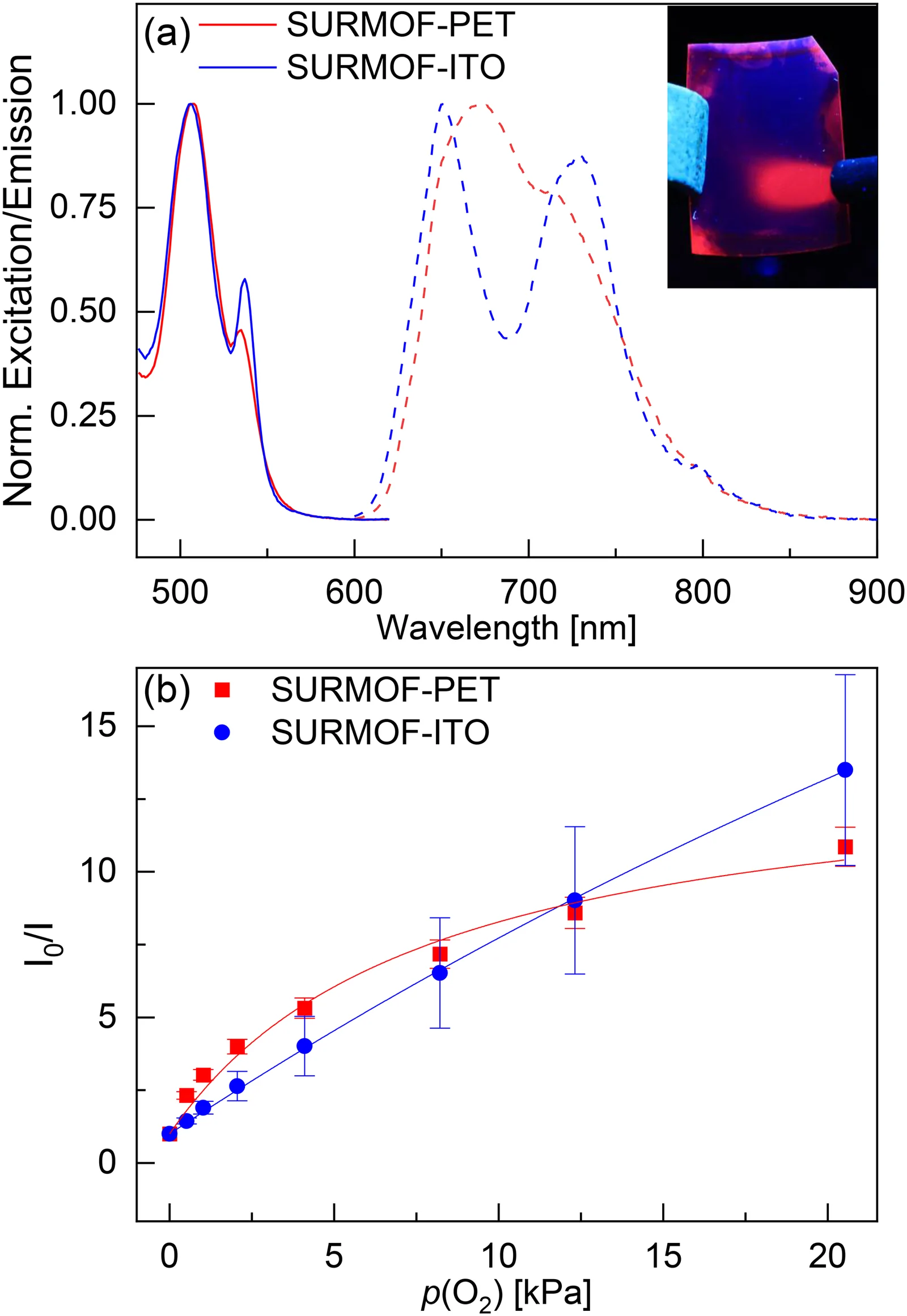
(a) Normalized emission and excitation spectra of SURMOF-PET and SURMOF-ITO. Red lines: excitation (solid line, *λ*_em_ = 670 nm) and emission (dashed line, *λ*_exc_ = 400 nm) of SURMOF-PET, acquired under anoxic conditions; blue lines: excitation (solid line, *λ*_em_ = 650 nm) and emission (dashed line, *λ*_exc_ = 400 nm) of SURMOF-ITO, acquired under anoxic conditions. The photographic image shows the luminescence of the coating on the PET foil under UV excitation and nitrogen jet. (b) Stern–Volmer plots of SURMOF-PET and SURMOF-ITO. The intensity was measured at the maximum of the emission spectrum (*λ*_ex_ = 400 nm).

The coating on the COOH-modified PET substrate remains functional over many cycles (Fig. S38D of the SI), demonstrating the feasibility of the approach for preparation of oxygen-sensitive SURMOFs on flexible substrates.

### 3.5. Photophysical properties of the SURMOFs in toluene

To test the feasibility of oxygen sensing in organic solvents using SURMOFs, we investigated the spectral and oxygen-sensing properties of the coatings submerged in toluene. New SURMOFs were fabricated with the same parameters and labeled SURMOF-DMF-38d and SURMOF-EtOH-38d, respectively. The SURMOFs prepared from DMF and EtOH retained their characteristic excitation and emission bands in toluene (Fig. S40A and B of the SI). The sensitivity is greatly reduced compared to the gas phase (Fig. 7, SURMOF-DMF-38d: *K*_SV1_ = 0.18 kPa^−1^; SURMOF-EtOH: *K*_SV1_ = 0.095 kPa^−1^). This is not surprising, considering the much slower diffusion of oxygen in the condensed phase (diffusion coefficient of 4.38 × 10^−9^ m^2^ s^−1^ in toluene^64^ compared to the diffusion coefficient of 1.98 × 10^−5^ m^2^ s^−1^ in air^65^). A similar effect has been documented before for Pt(ii)PCN-224 powder MOFs that showed significant reduction in oxygen sensitivity when going from the gas phase to aqueous media (*I*_0_/*I*_0.2 kPa_ ∼ 5 and *K*_SV1_ = 73 kPa^−1^ in the gas phase; *I*_0_/*I*_20 kPa_ ∼3 and *K*_SV1_ = 0.0113 µM^−1^ (0.14 kPa^−1^) in aqueous solution).^27,59^ Despite these changes in sensitivity, the SURMOFs proved suitable for sensing oxygen in an organic solvent, albeit not at trace levels. Additionally, both SURMOFs tested showed highly repeatable responses with nearly no drift of the luminescence intensity during several cycles. Notably, both samples remain mechanically stable during the measurements.

**Fig. 7:**
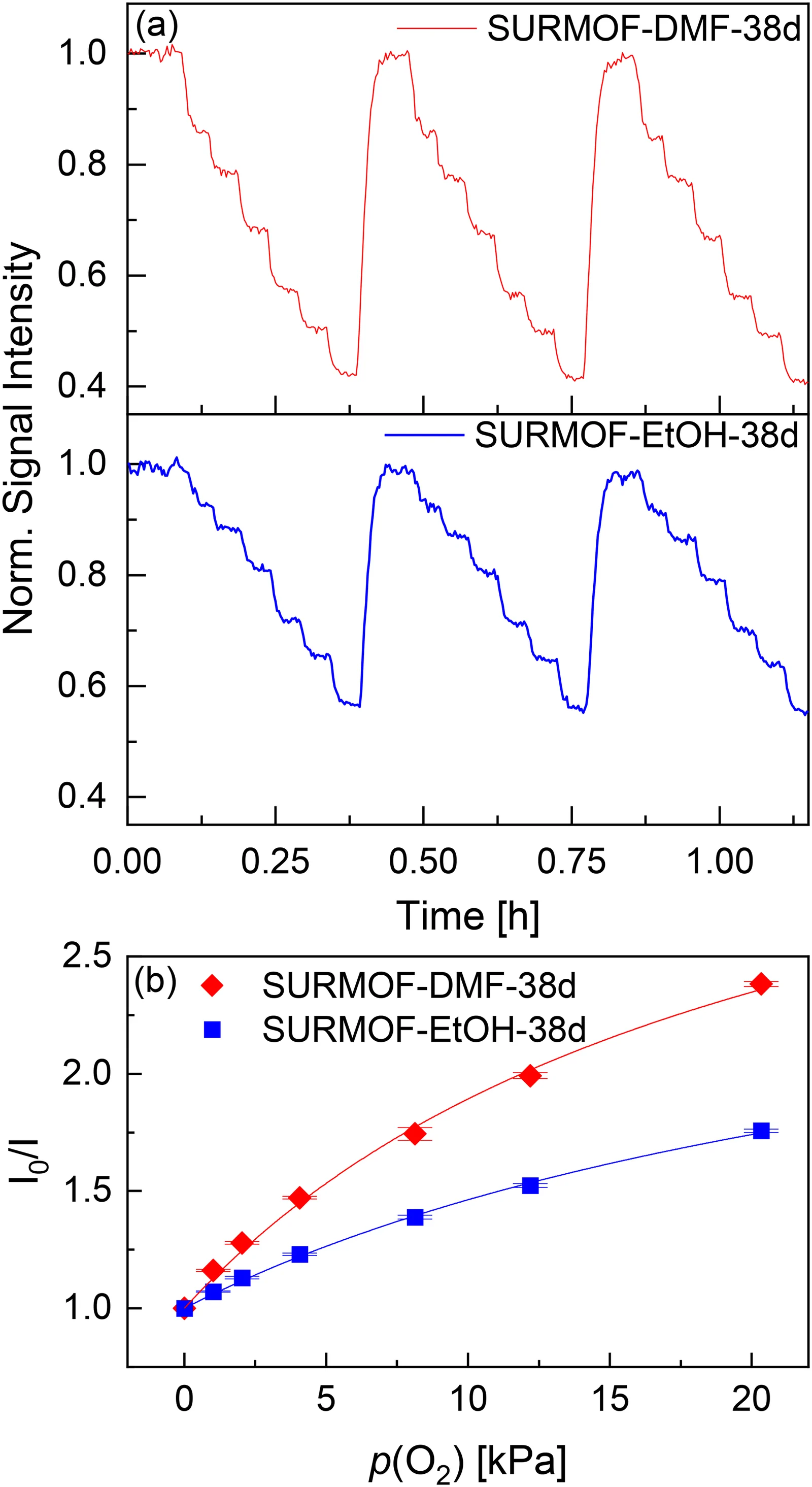
(a) Phosphorescence response of SURMOFs recorded in toluene at 20 °C to alternating oxygen concentration (N_2_, 1.0 kPa, 2.0 kPa, 4.1 kPa, 8.1 kPa, 12.2 kPa and 20.3 kPa) monitored at the emission maximum (*λ*_ex_ = 400 nm) and (b) Stern–Volmer plots of SURMOF-DMF-38d and SURMOF-EtOH-38-d in toluene monitored at the emission maximum (*λ*_ex_ = 400 nm).

### 3.6. Stability of SURMOFs

Luminescent powder MOFs are usually activated at elevated temperatures under vacuum,^18^ since this procedure helps remove residues that may block the pores and slow down oxygen diffusion. Rather surprisingly, the same procedure applied to SURMOFs (7 days, room temperature, and vacuum) results in a drastic decrease in the emission intensity and the sensitivity to oxygen (Fig. S42, SI). The phosphorescence decay time also decreased to ∼26 µs. We noticed the appearance of an additional absorption band (∼585 nm) after activation, which was also observed in the absorption spectra of the SURMOFs stored for a prolonged time (several months) in darkness under ambient conditions (Fig. S28 of the SI). The position of this band is similar to that evolving during the photobleaching of Pt(ii)TCPP in DMF solution.^46^ Additionally, the activation process leads to an increase of the background in the absorption spectra and the bathochromic shift of the Q-bands (Fig. S43 of the SI).

We also noticed NIR phosphorescence upon excitation in the new absorption band (Fig. S42D, SI). This may originate from chlorin species formed *via* reduction of the porphyrin linker by the residual solvent or its decomposition products. In fact, Pt(ii) chlorins of *meso*-unsubstituted porphyrins were reported to show absorption and phosphorescence bands that are bathochromically shifted compared to porphyrins.^66^ It is expected that different factors such as light exposure during the fabrication and storage, humidity, and the amount of trapped guest molecules affect the (photo)degradation of SURMOFs and formation of new species. In fact, a similar effect has been documented before by Adams and co-workers, who suggested the formation of a chlorin derivative upon photodegradation of Pd(ii) porphyrin-based MOFs under light exposure.^67^ Here, entrapped residual solvent molecules are likely to act as a proton source, promoting the reduction process from Pd(ii) porphyrin to Pd(ii) chlorin.^67^ Therefore, we believe that the investigation of SURMOF deposition from other solvents (which either are not involved in the reduction or are easier to remove in the solvent exchange process) may be potentially helpful to mitigate the photobleaching issue in the future. Moreover, a more detailed investigation of the degradation process is considered particularly promising and valuable, as well as the NIR-emissive properties of the formed species.

On the other hand, since only a very minor amount of the new NIR emitting species is formed during drying in a vacuum, this conversion is not likely to be responsible for the drastic change in the emissive properties of the SURMOF. More likely, the bathochromic shift may indicate a significant change in the polarity of the microenvironment of the porphyrin linker due to removal of the residual solvent molecules and activation of non-radiative deactivation pathways. Interestingly, for one of the earlier batches of the SURMOF, obtained in preliminary experiments with similar settings as the SURMOF-DMF-38 series, we did not notice any significant changes in the GIWAX pattern after the vacuum treatment, suggesting that the crystal structure of the framework is not significantly altered during the vacuum drying process (Fig. S44).

The photostability of the SURMOFs was further evaluated by exposing them to continuous irradiation with a Xe-lamp (in contrast to discontinuous acquisition (1 m.p./10 s) used for other experiments) under alternating anoxic and air-saturated conditions (Fig. S41 of the SI). Photodegradation of the SURMOFs was found to be particularly fast in the first 15 min of irradiation when the SURMOFs are exposed to an anoxic atmosphere, especially in the gas phase. We believe that the degradation mechanism may involve entrapped solvent molecules that promote photoreduction to chlorin species under anoxic conditions. After the initial exposure period, the SURMOFs in the gas atmosphere appear to degrade both under anoxic and air-saturated conditions.

For the material submerged in toluene, bleaching appears to occur predominantly under air-saturated conditions (strong intensity drop after 15 min in air and a much smaller decrease during the anoxic period). Degradation of MOFs under air-saturated conditions suggests the potential role of photosensitized singlet oxygen in photodegradation. Therefore, the rather complex photobleaching behavior with degradation under both anoxic and air-saturated conditions suggests that different pathways may be favored in different environments,^68^ which might be due to stabilization of certain motifs (*e.g.*, carboxylate groups). Undoubtedly, photodegradation of the SURMOFs is a serious drawback for potential applications; however, they can still be considered usable for short-term applications. In fact, continuous irradiation is not needed and acquisition of a measuring point typically requires 20–100 ms integration time (signal and equipment dependent), translating to thousands of measuring points until the photodegradation becomes significant and requires sensor recalibration.

## 4. Conclusions

Several luminescent SURMOFs built from Pt(ii)porphyrins and In–oxo-clusters have been successfully fabricated on the transparent substrates. We demonstrated the growth of the SURMOF on rigid glass and flexible, transparent PET substrates, both chemically pre-treated to generate COOH groups on the surface. Evaluation of DMF as an alternative solvent for SURMOF fabrication revealed higher homogeneity of the resulting layers and more reproducible oxygen sensing properties compared to ethanol, supposedly due to the much higher boiling point of the former.

Whereas the GIWAXS measurements confirmed the crystallinity of the SURMOFs, SEM images indicated strong variation in morphologies of different samples, presumably due to poorly controllable factors during the fabrication process. Importantly, the morphology does not appear to significantly affect the oxygen sensitivity. All of the samples are composed of small MOF islands grown together, which introduces macropores beside the micropores of the SURMOF crystal lattice. The microporosity of the MOFs is thus held responsible for the very efficient luminescence quenching by molecular oxygen in the gas phase, which is generally comparable to the response of the crystalline powder MOFs prepared from the same indicator and indium–oxo-clusters but several times lower than that for the PCN-type composed of the Pt(ii) porphyrin and zirconium–oxo-clusters.

Importantly, the SURMOFs remain functional in toluene, paving the way to their use for sensing oxygen dissolved in organic solvents, which is currently not possible with established state-of-the-art oxygen-sensing materials. However, rather fast photodegradation remains a limitation, as traces of entrapped solvents or their decomposition products are possibly causing particularly fast photodegradation during the initial illumination period. Therefore, investigating SURMOF deposition from a broader range of organic solvents also remains of high interest.

## Author contributions

T. K. M. performed most of the experiments, analyzed the data and prepared the initial draft of the manuscript. F. G. provided structural characterization and F. W. performed SEM measurements. R. R. gave expert advice and support in the interpretation of results. C. S. and S. B. conceived, supervised and directed the study and acquired the funding. All authors contributed to the analysis of the results and to the writing of the manuscript.

## Conflicts of interest

The authors have no conflicts of interest to declare.

## Supplementary Material

TC-014-D6TC02088G-s001

TC-014-D6TC02088G-s002

## Data Availability

The data supporting the paper are available in the supplementary information (SI). Supplementary information: the AFM, SEM and GIWAXS characterization of the SURMOFS, absorption, excitation and emission spectra of the dissolved porphyrins and SURMOFs, luminescence decays, and details on synthesis, activation and photodegradation of SURMOFs. See DOI: https://doi.org/10.1039/d6tc02088g.
