# Supplementary material for: Phosphorescent surface-anchored metal–organic frameworks for optical sensing of oxygen
Source: J Mater Chem C Mater. 2026 Oct 5;14(43):20919–30. doi: 10.1039/d6tc02088g (PMC13659863; doi:10.1039/d6tc02088g)
Supplement: Supplementary file 2 [file TC-014-D6TC02088G-s001.pdf]

Supplementary Information (SI) for Journal of Materials Chemistry C.
This journal is © The Royal Society of Chemistry

Supporting Information

Phosphorescent Surface-Anchored Metal-Organic-Frameworks for Optical Sensing of Oxygen

Theresa K. Mautz[1], Fabian Gasser[2], Roland Resel[2], Fernando G. Warchomicka[3], Christian Slugovc[4], Sergey M. Borisov[1*]

[1] Institute of Analytical Chemistry and Food Chemistry, Graz University of Technology, 8010 Graz.

[2] Institute of Solid State Physics, Graz University of Technology, 8010 Graz, Austria.

[3] Institute of Materials Science, Joining and Forming, Graz University of Technology, 8010 Graz, Austria.

[4] Institute for Chemistry and Technology of Materials, Graz University of Technology, 8010 Graz, Austria.

S1. NMR spectra of meso-8F-PtTCPP 3
S2. Fabrication of SURMOFs 4
S3. Structural Characterization 5
S3.1. Atomic Force Microscopy 5
S3.2. Scanning Electron Microscopy 9
S3.3. Grazing-Incidence X-Ray Diffraction 15
S4. Photophysical Properties and Oxygen Sensitivity of SURMOFs 20
in Gas Phase 20
S3.1 Absorption and Emission Spectra of Dissolved Porphyrins 20
S3.2 Absorption, Excitation and Emission Spectra of Porphyrin-based SURMOFs 21
S3.3 Luminescence Decays 24
S3.4 SURMOFs based on Partly Fluorinated Porphyrin 28
S3.5 Photophysical Properties and Oxygen Sensitivity of SURMOFs on Flexible Materials 29
S3.6. Photophysical Properties and Oxygen Sensitivity of SURMOFs in Toluene 30
S3.7. Photodegradation of SURMOFs in Gas Phase and Toluene 32
S3.8 Activation and Sensitivity Loss 33
S5. In-Pt(II)TCPP powder MOFs 35
S5.1 Synthesis and Activation of In-Pt(II)TCPP powder MOF 35
S5.2 Photophysical and Oxygen Sensing Properties of In-Pt(II)TCPP powder MOF in the Gas Phase 36
S5.3 Thermogravimetric analysis of In-Pt(II)TCPP powder MOF 38
S6. Overview of reported porphyrinic SURMOFs 39
S7. Overview of porous materials for optical sensing of oxygen 40
S8. References 41

S1. NMR spectra of meso-8F-PtTCPP

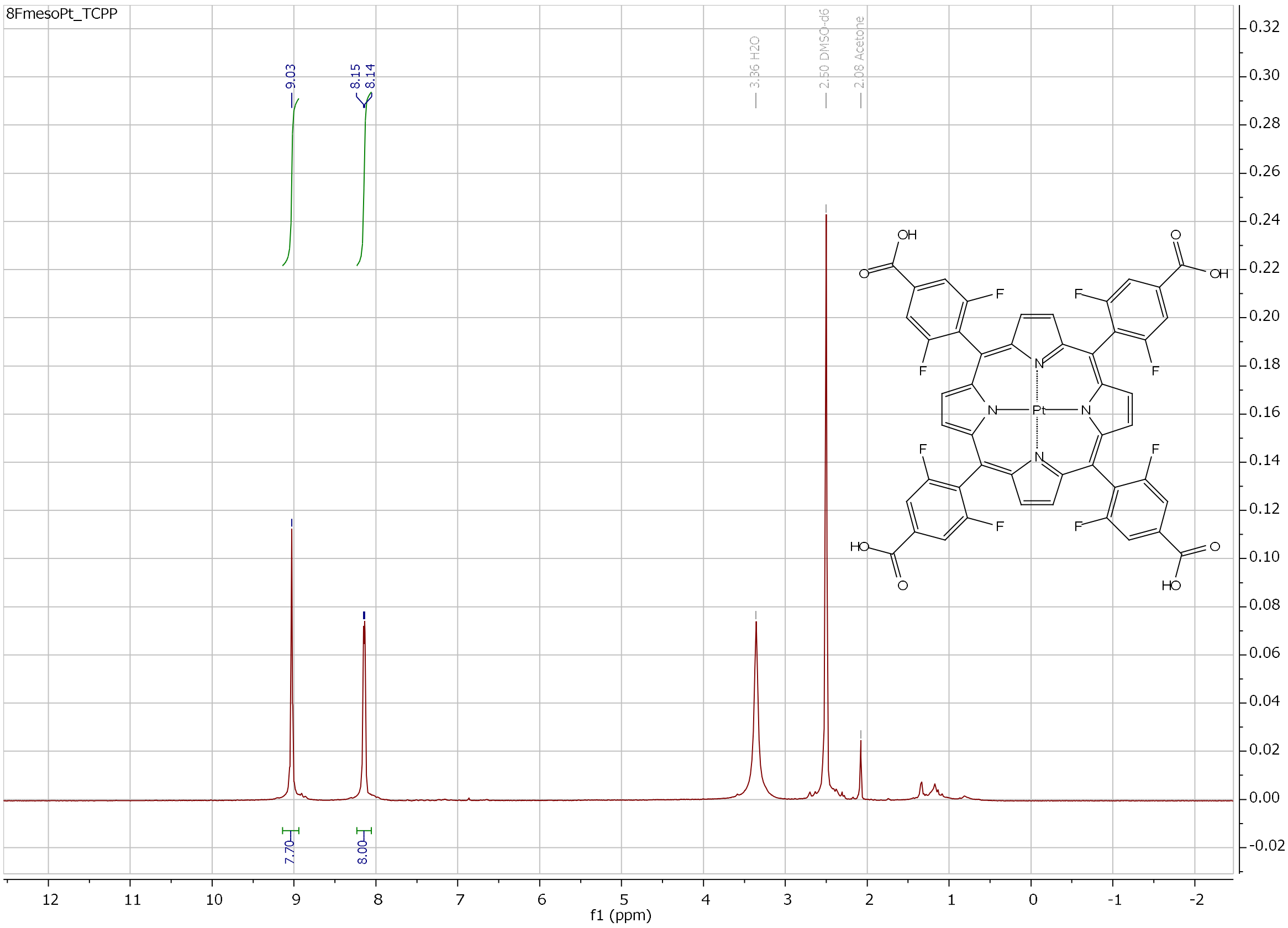

Figure S1: ^{1}H NMR spectrum (500 MHz; DMSO-d6) of meso-8F-PtTCPP*: δ 9.03 (s, 8H), 8.14 (d, J = 6.8 Hz, 8H).*

S2. Fabrication of SURMOFs

Pt(II)TCPP/In-oxo-cluster based surface-anchored-metal-organic-frameworks (SURMOFs) were fabricated by liquid-phase epitaxy (LPE) in the layer-by-layer (LbL) dipping fashion: the modified substrate was subsequently immersed in an organic linker solution and a metal node solution, whereby immersion time was set to 20 min. Between each step, 4 washing steps were performed by immersion of the substrate into the corresponding solvent for 1 min. For each sample 85 cycles (total fabrication time 72 h) were performed.

Immediately after the deposition, the samples were rinsed with the corresponding solvent to remove remaining building blocks. Afterwards, the samples were immersed in ethanol and methanol for several hours at room temperature to allow the diffusion of uncoordinated linkers from the pores. The solvent was exchanged multiple times. The samples were stored in ethanol in darkness until used.

Table S1:Experimental conditions for SURMOFs. SURMOF-DMF-38X and SURMOF-EtOH-38X was fabricated multiple times and named -a, -b, -c, -d, -e and -f.

	**Organic Linker**	**Organic Linker Conc. [µM]**	**Metal Source**	**Metal Node Conc. [mM]**	**Solvent**	**Substrate**
SURMOF-DMF-38X	Pt(II)TCPP	38	$InCl_3$	1.1	DMF	glass
SURMOF-DMF-25	Pt(II)TCPP	25	$InCl_3$	1.1	DMF	glass
SURMOF-DMF-50	Pt(II)TCPP	50	$InCl_3$	1.1	DMF	glass
SURMOF-DMF-8F	*meso*-8F-Pt(II)TCPP	38	$InCl_3$	1.1	DMF	glass
SURMOF-EtOH-38X	Pt(II)TCPP	38	$InCl_3$	1.1	EtOH	glass
SURMOF-ITO	Pt(II)TCPP	38	$InCl_3$	1.1	DMF	PET-ITO
SURMOF-PET	Pt(II)TCPP	38	$InCl_3$	1.1	DMF	PET-COOH

S3. Structural Characterization

S3.1. Atomic Force Microscopy

Atomic Force Microscopy (AFM) images were recorded for SURMOF-DMF-38a and SURMOF-EtOH-38a to evaluate the film thickness. The SURMOF was partly removed with a spatula and the respective area along the scratch was recorded. The average film thickness was calculated from the mean value of three height profiles for each sample (Figure S4-S6 and Figure S9-S11).

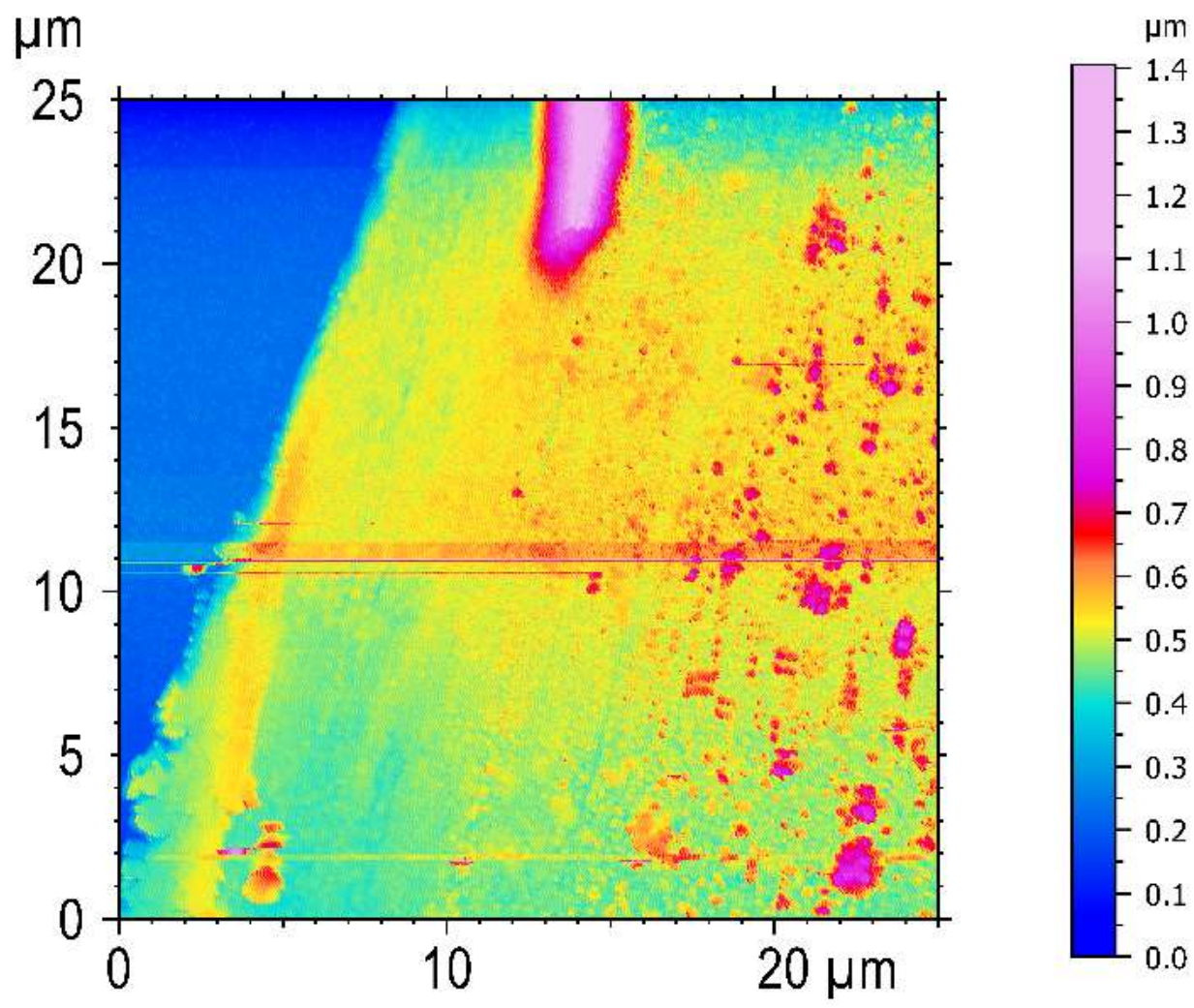

Figure S2: Topographic AFM image of SURMOF-DMF-38a. The blue area represents the glass surface, while the green/yellow area shows the surface of the SURMOF.

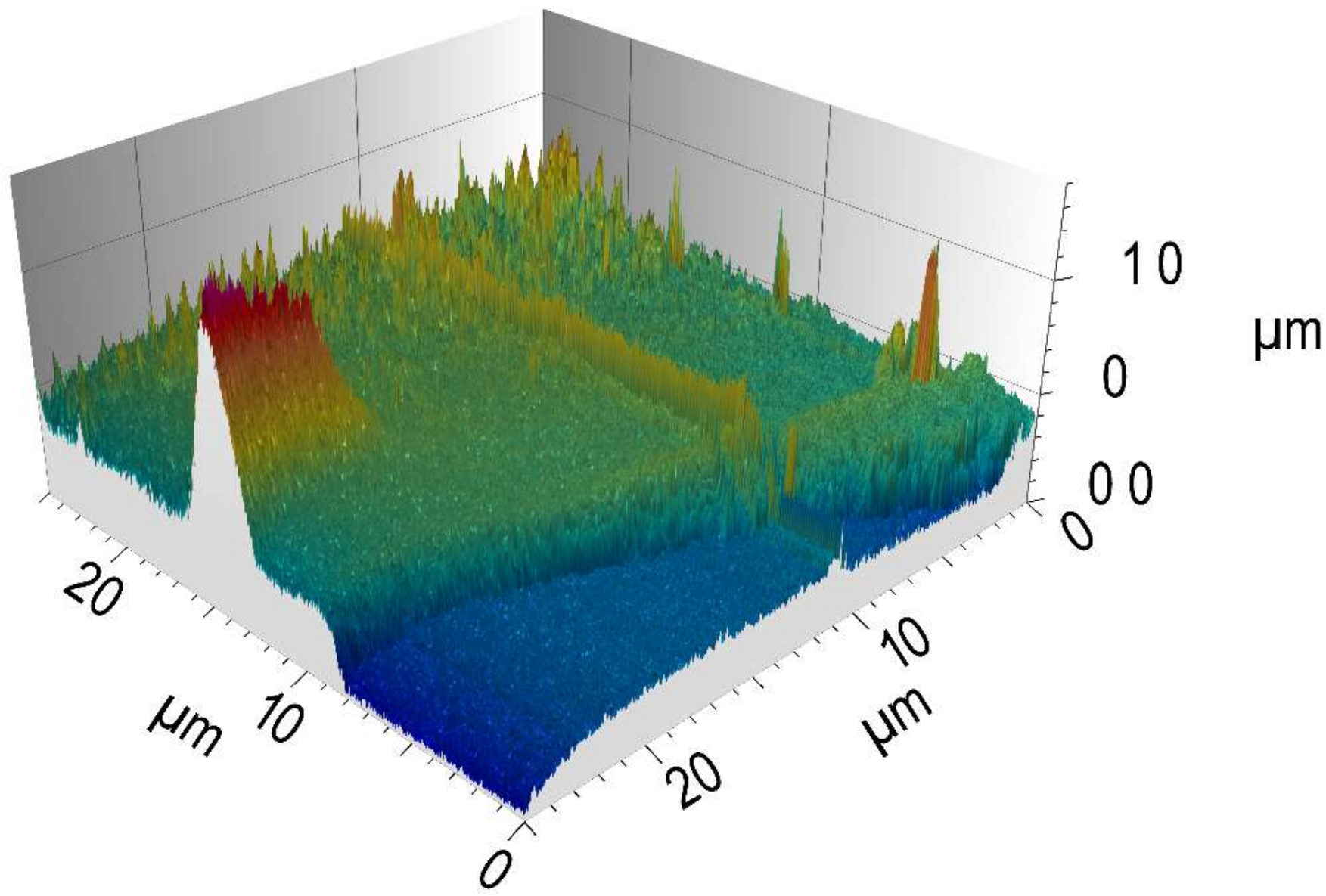

Figure S3: Three-dimensional AFM image of SURMOF-DMF-38a.

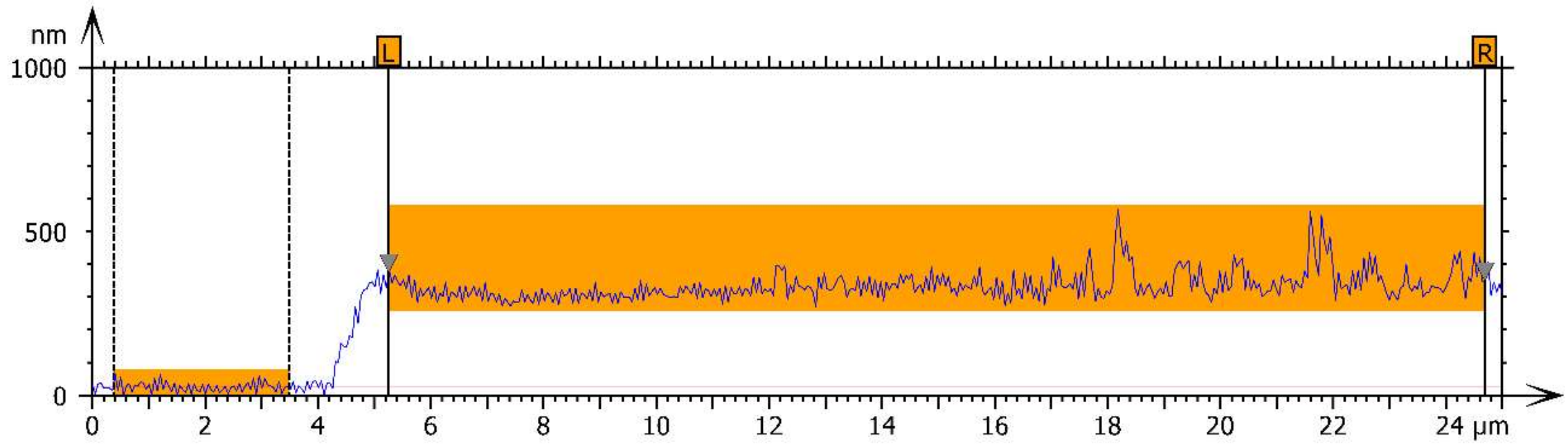

Parameters	Step 1	Unit
Width	19.5	µm
Maximum height	541	nm
Mean height	310	nm

Figure S4: Height profile 1 of SURMOF-DMF-38a.

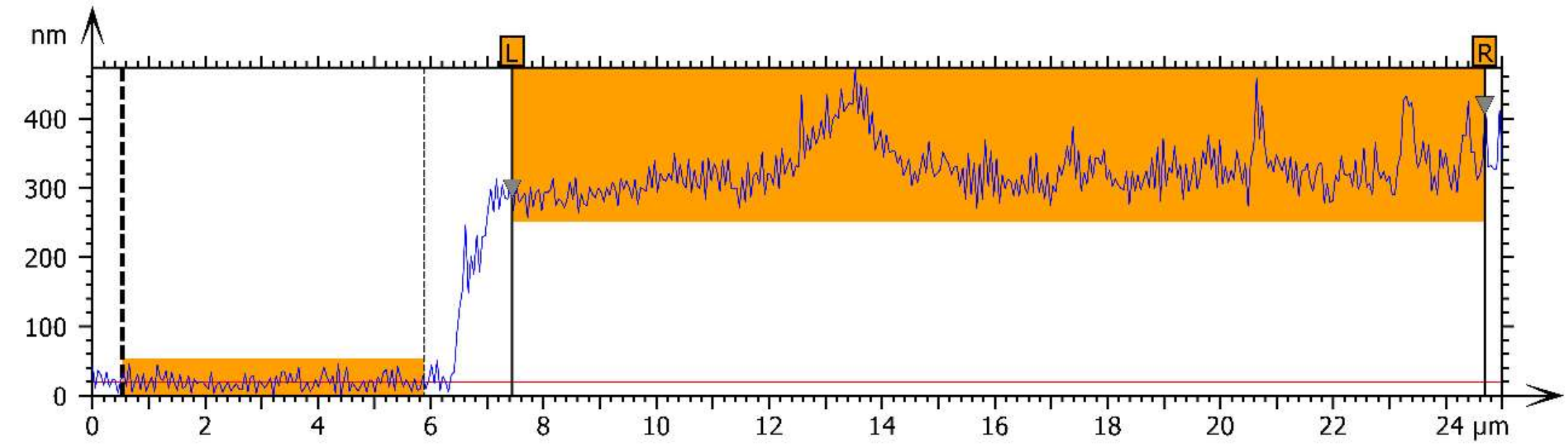

Parameters	Step 1	Unit
Width	17.3	µm
Maximum height	454	nm
Mean height	307	nm

Figure S5: Height profile 2 of SURMOF-DMF-38a.

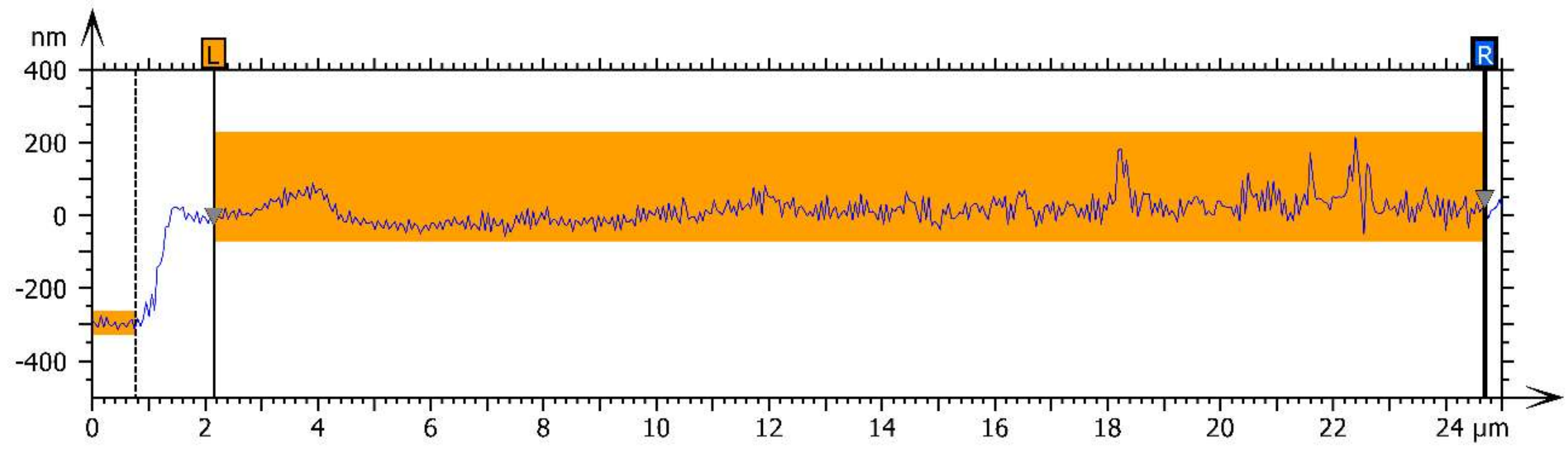

Parameters	Step 1	Unit
Width	22.6	µm
Maximum height	513	nm
Mean height	313	nm

Figure S6: Height profile 3 of SURMOF-DMF-38a.

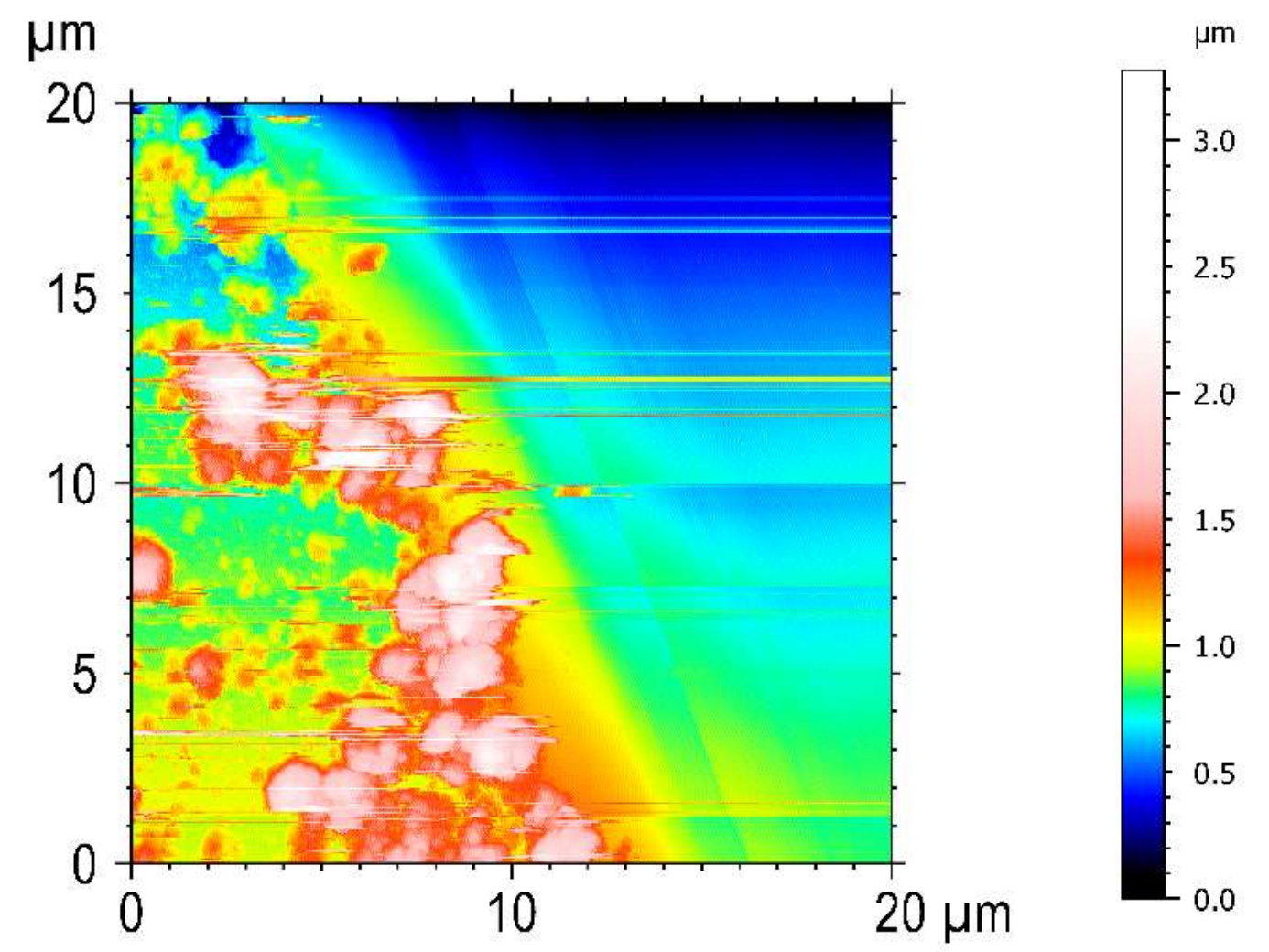

Figure S7: Topographic AFM image of SURMOF-EtOH-38a. The blue area represents the substrate surface, while the green/red parts shows the surface of the SURMOF.

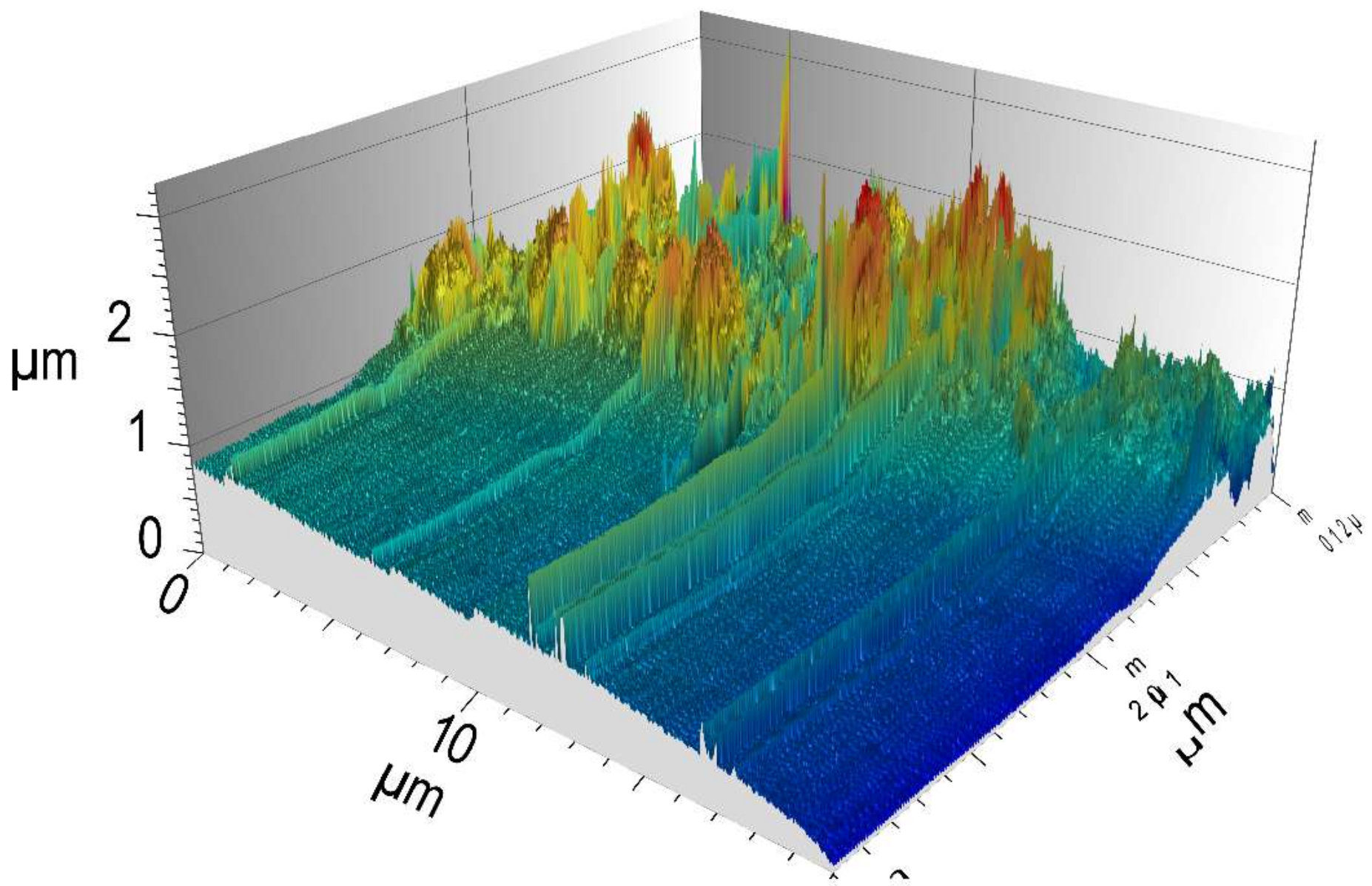

Figure S8: Three-dimensional AFM image of SURMOF-EtOH-38a.

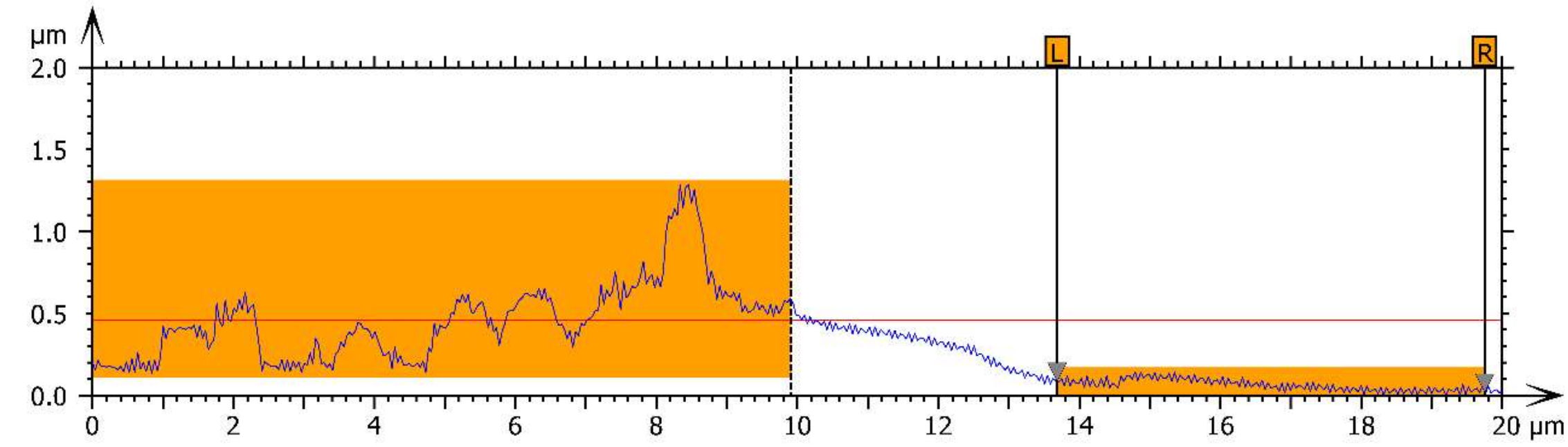

Parameters	Step 1	Unit
Width	6.09	µm
Maximum height	0.458	µm
Mean height	0.395	µm

Figure S9: Height profile 1 of SURMOF-EtOH-38a.

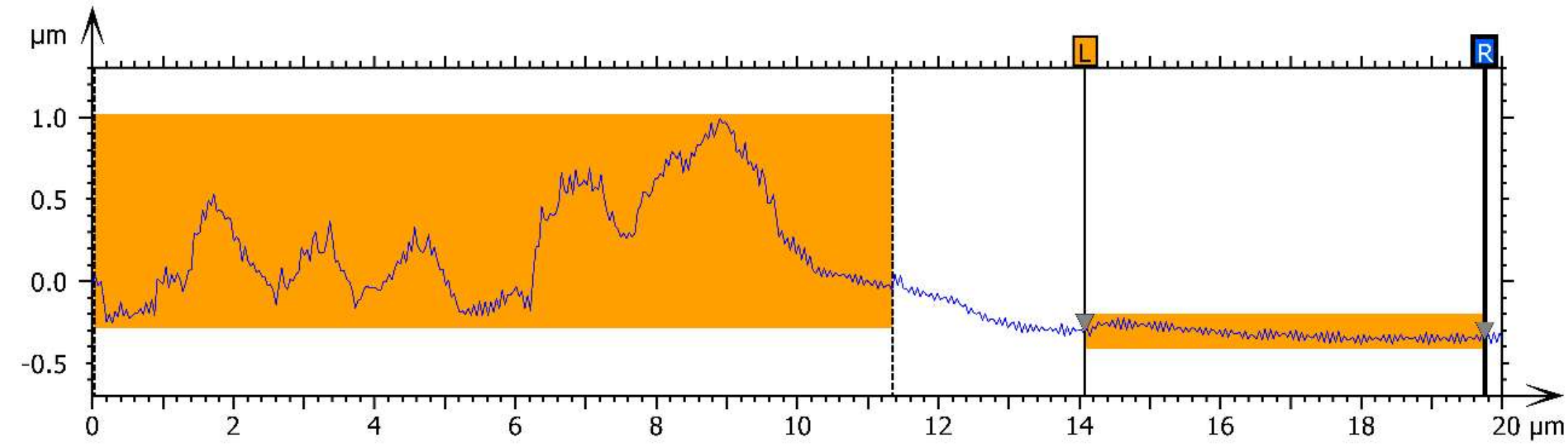

Parameters	Step 1	Unit
Width	5.69	µm
Maximum height	0.599	µm
Mean height	0.534	µm

Figure S10: Height Profile 2 of SURMOF-EtOH-38a.

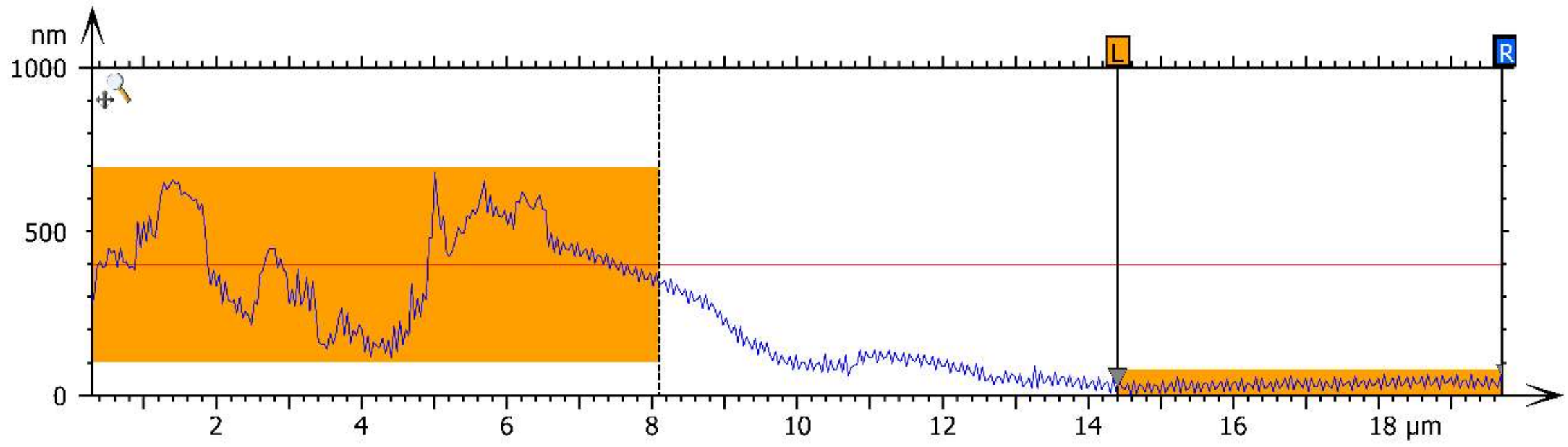

Parameters	Step 1	Unit
Width	5.37	µm
Maximum height	400	nm
Mean height	366	nm

Figure S11: Height Profile 3 of SURMOF-EtOH-38a.

S3.2. Scanning Electron Microscopy

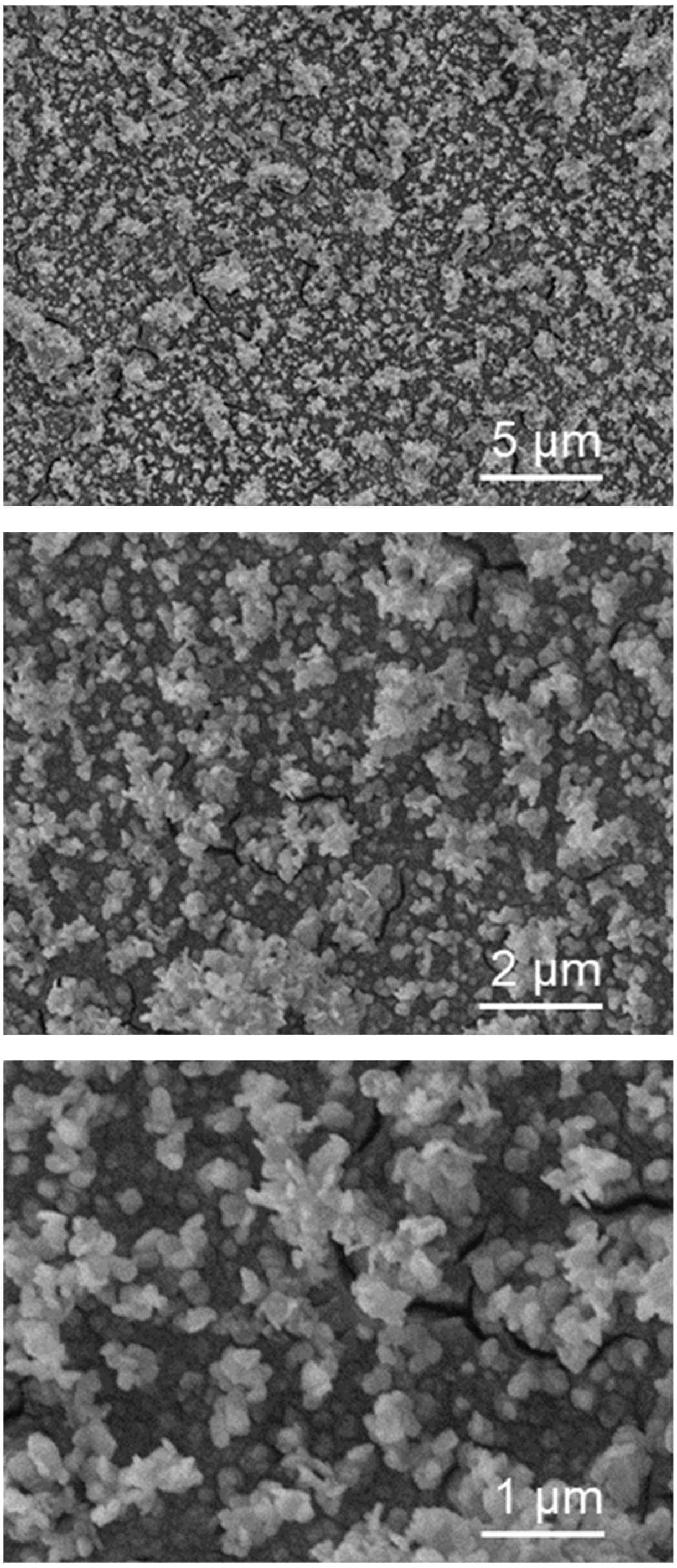

Figure S12: SEM image of SURMOF-DMF-25 at increasing magnification.

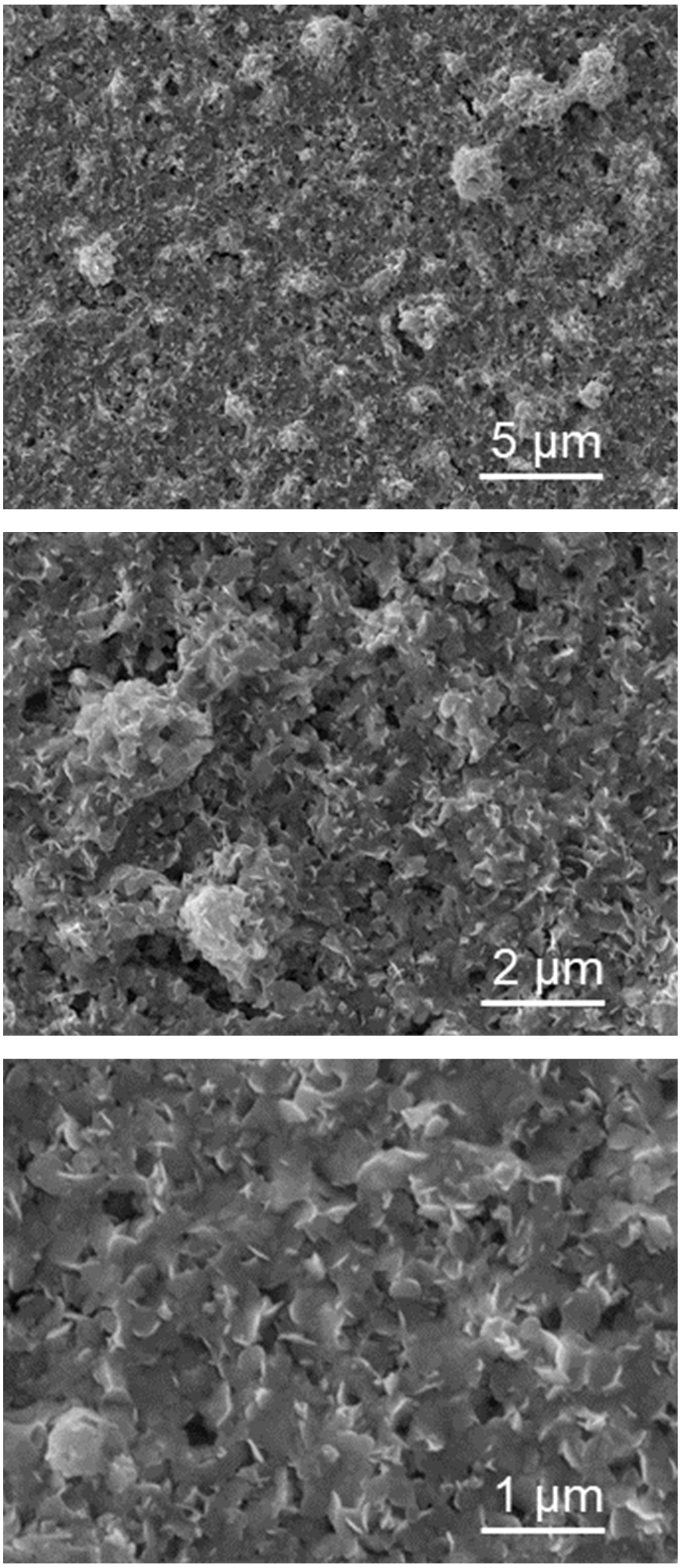

Figure S13: SEM image of SURMOF-DMF-50 at increasing magnification.

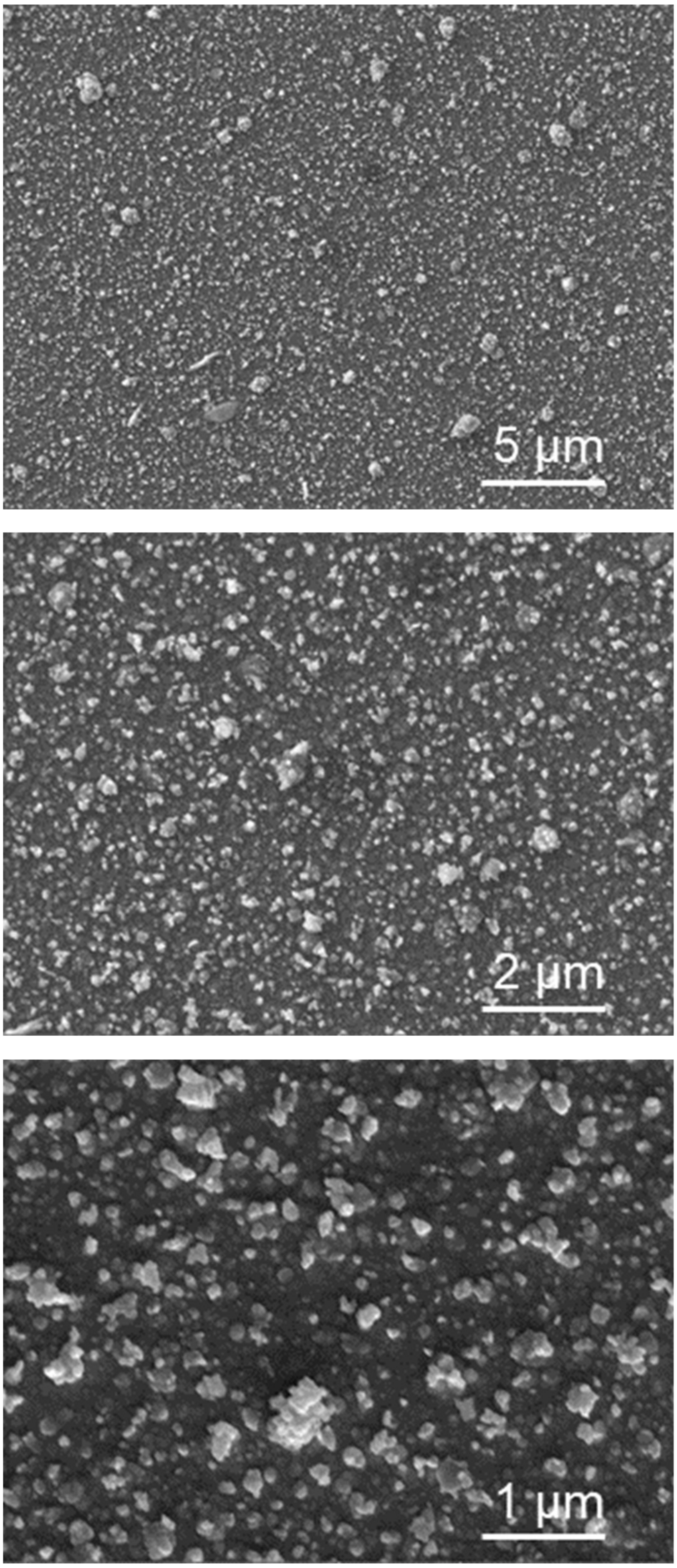

Figure S14: SEM image of SURMOF-DMF-38 at increasing magnification.

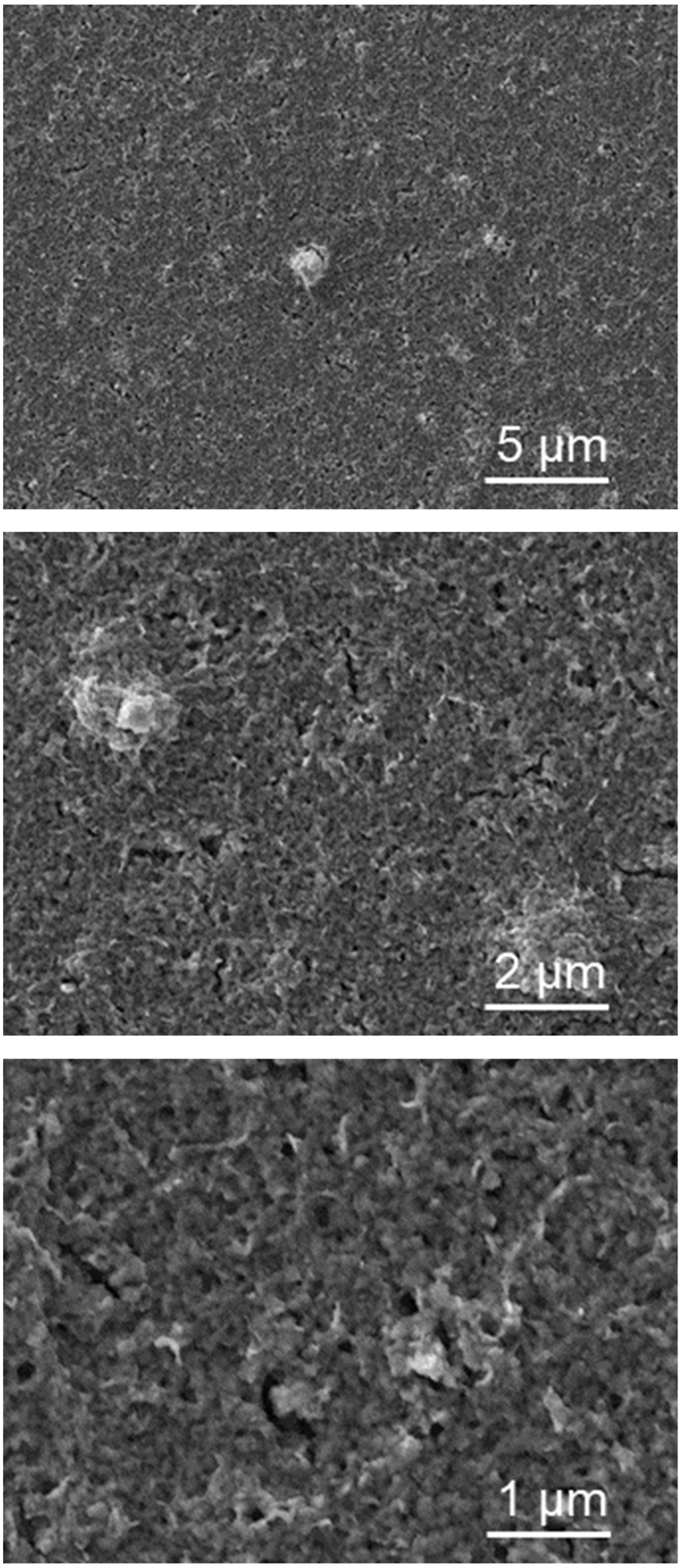

Figure S15: SEM image of SURMOF-DMF-38b at increasing magnification.

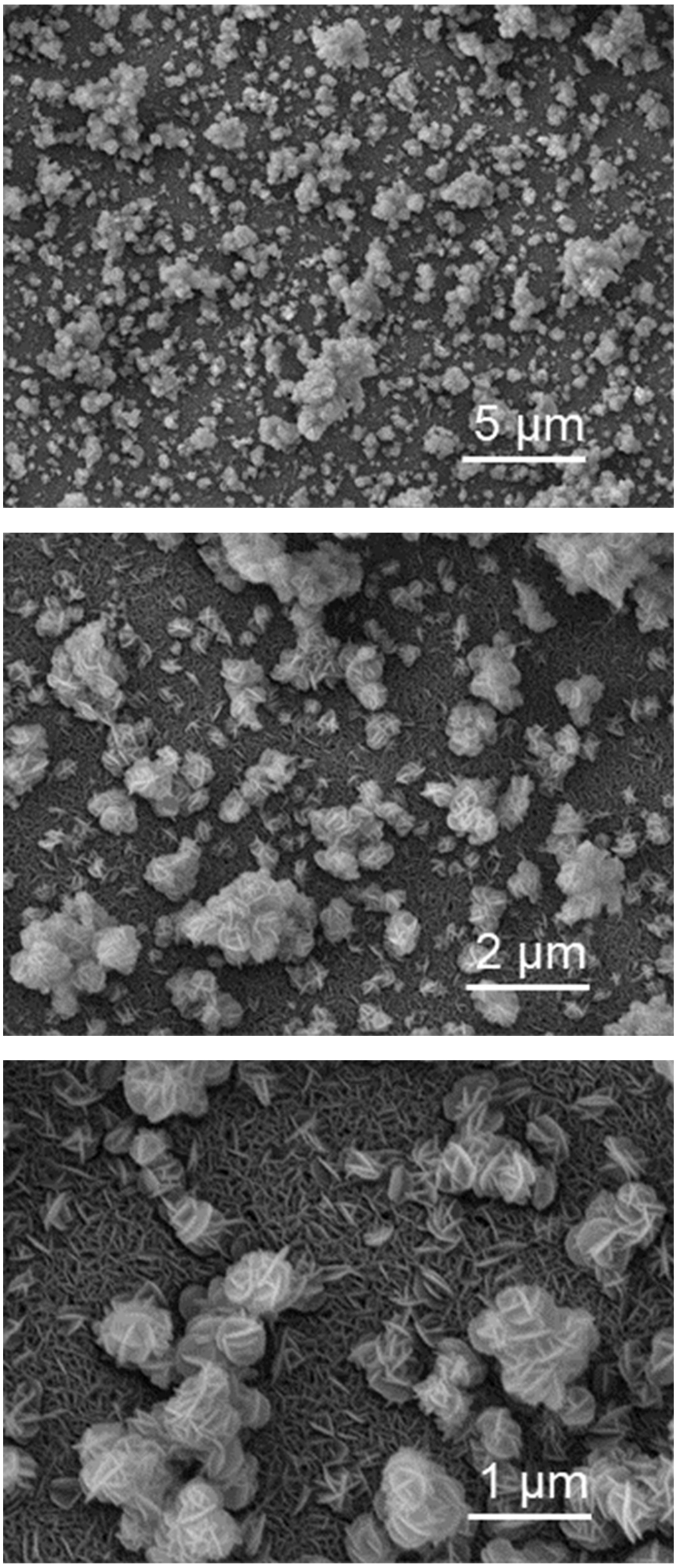

Figure S16: SEM image of SURMOF-EtOH-38 at increasing magnification.

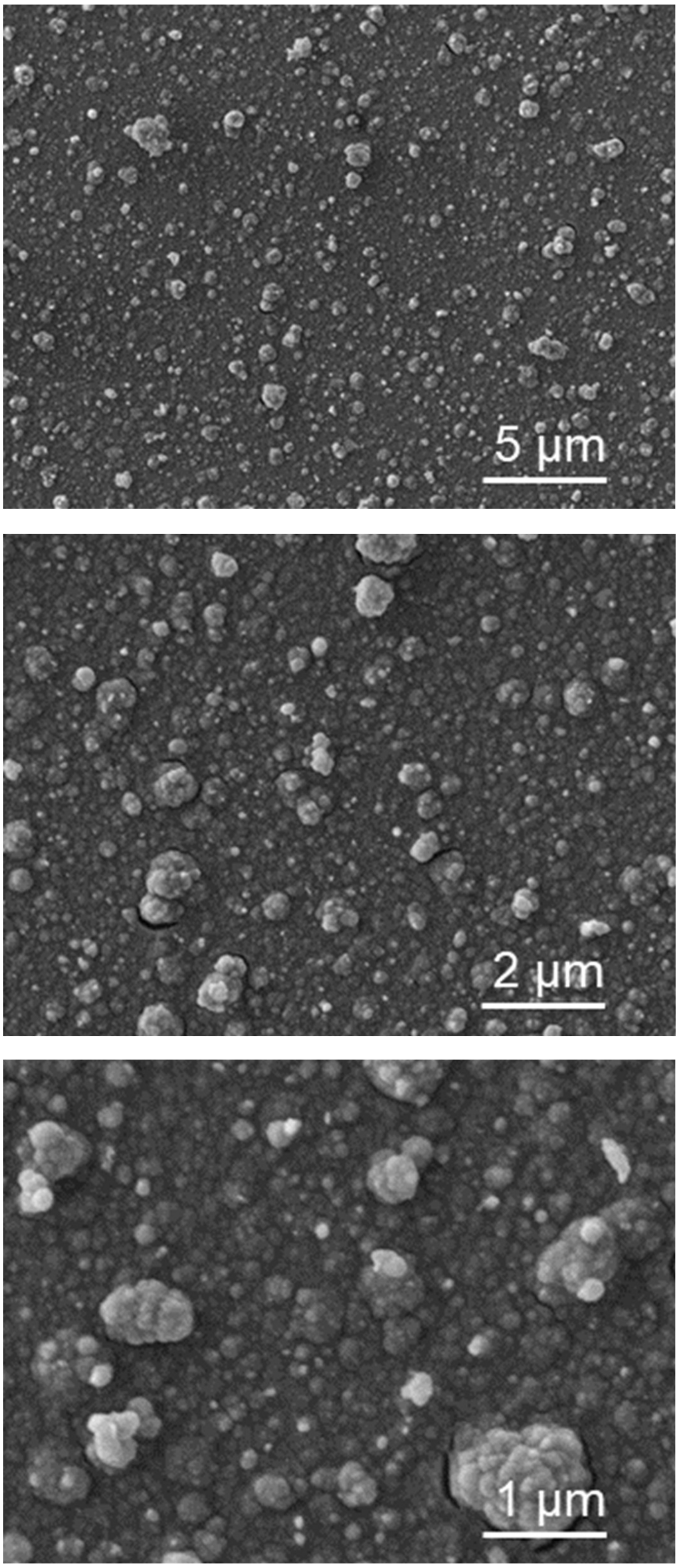

Figure S17: SEM image of SURMOF-DMF-8F at increasing magnification.

S3.3. Grazing-Incidence X-Ray Diffraction

Grazing-incidence wide-angle X-ray scattering (GIWAXS) measurements were performed on the XRD-1 beamline at Elettra Synchrotron in Trieste. To evaluate the structure, the obtained patterns were compared to reported out-of-plane XRD patterns of TCPP/In-Oxo-Cluster MOF.[1]

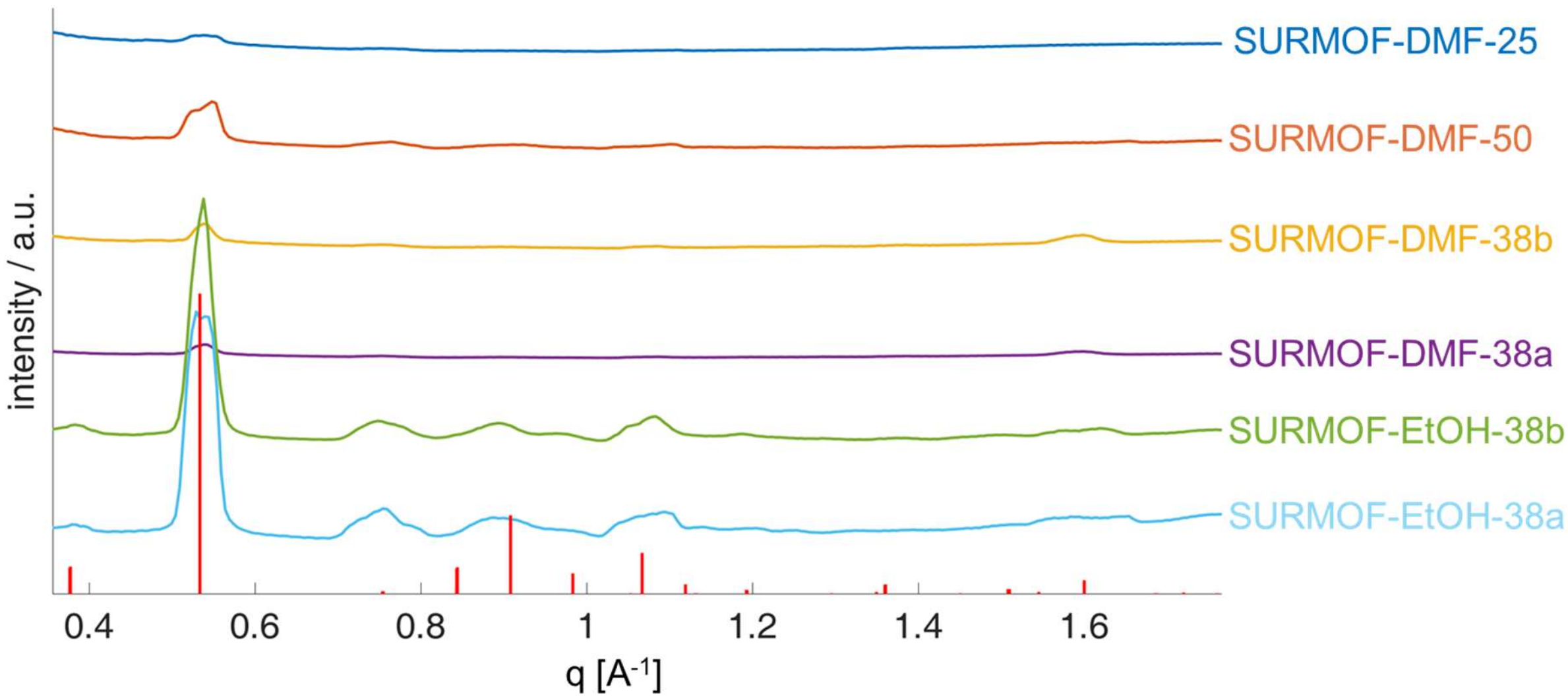

Figure S18: GIWAXS data from Pt(II)TCPP based SURMOFs. Azimuthal integration (along Debye-Scherrer rings) result in diffraction pattern as a function of the length of the scattering vector q. The vertical bars denote peak positions and peak intensities calculated on basis of the crystal structure of ERLAN. Please note, that the peak located at q = 0.75 Å-3 can be indexed by peaks with Laue indices 002 and / or 040 but with very small intensity.

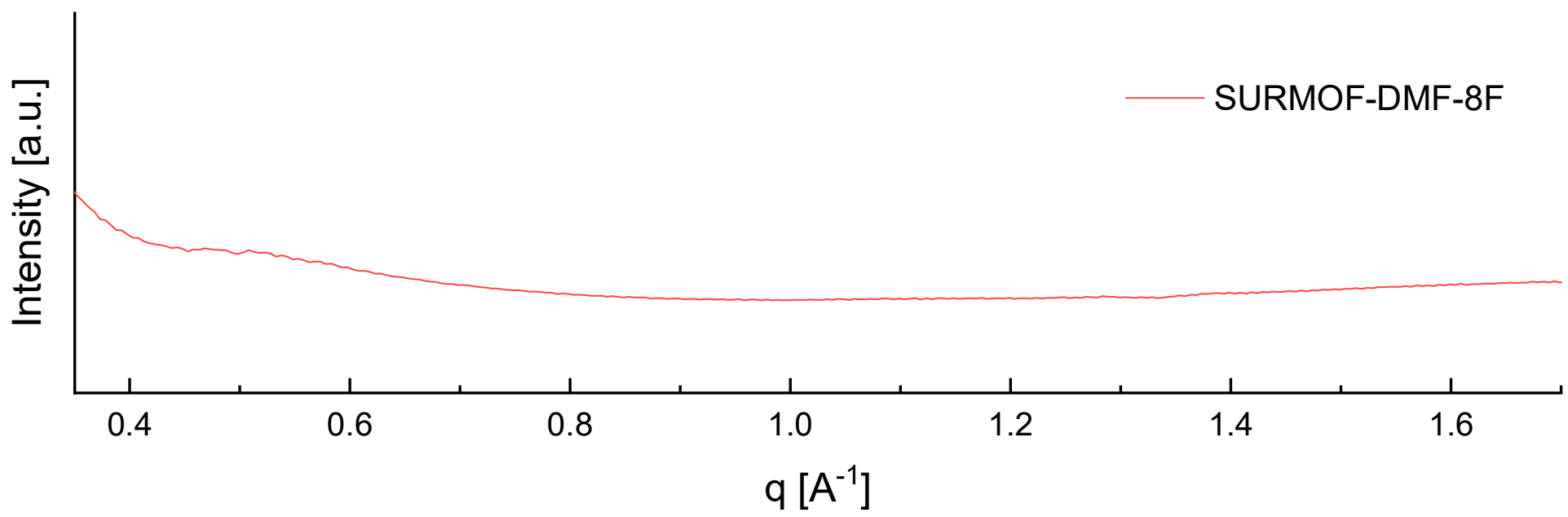

Figure S19: GIWAXS data from 8F-mesoPt(II)TCPP based SURMOF. Azimuthal integration (along Debye-Scherrer rings) result in diffraction pattern as a function of the length of the scattering vector q.

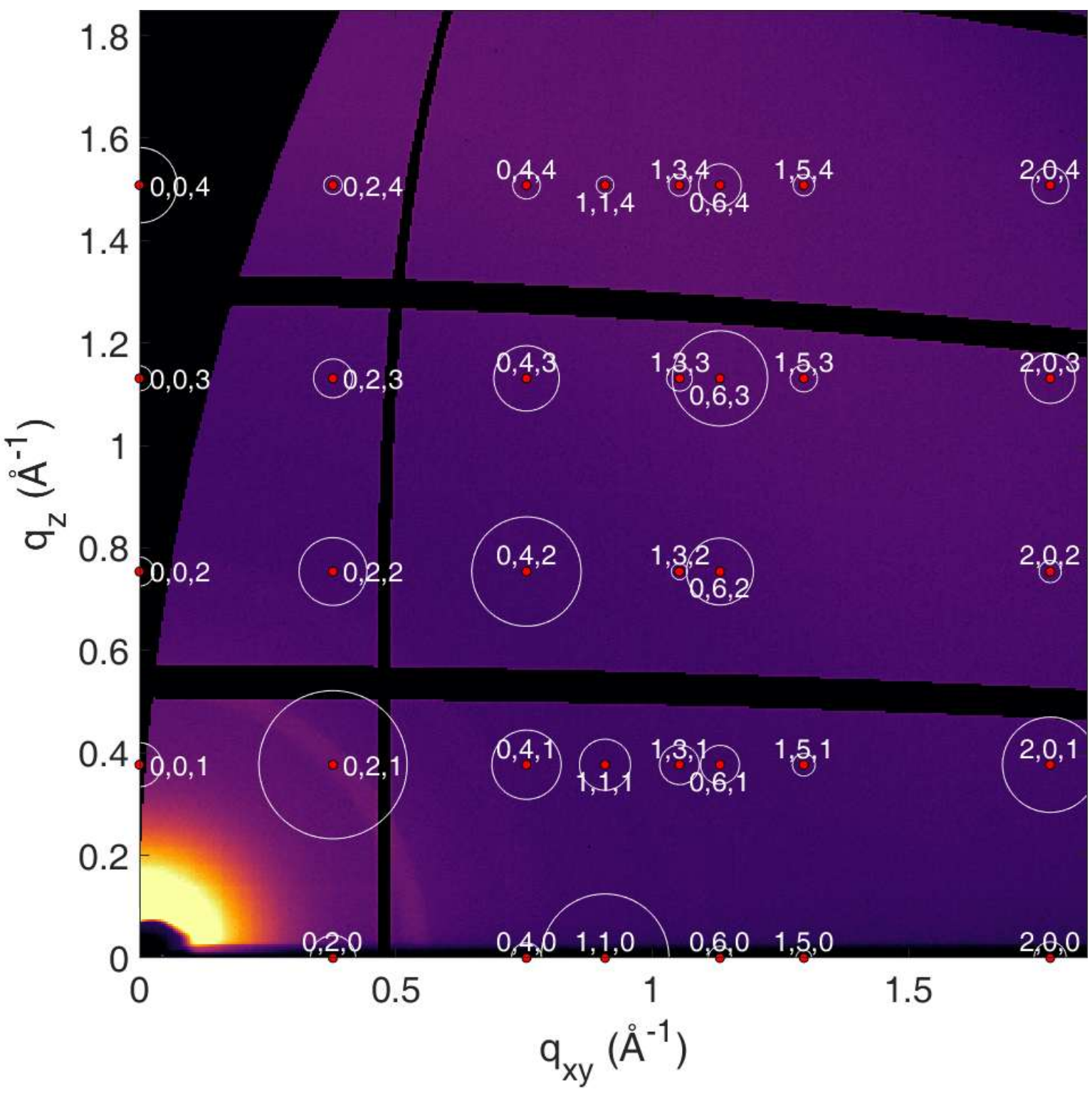

Figure S20: GIWAXS pattern of SURMOF-DMF-25.

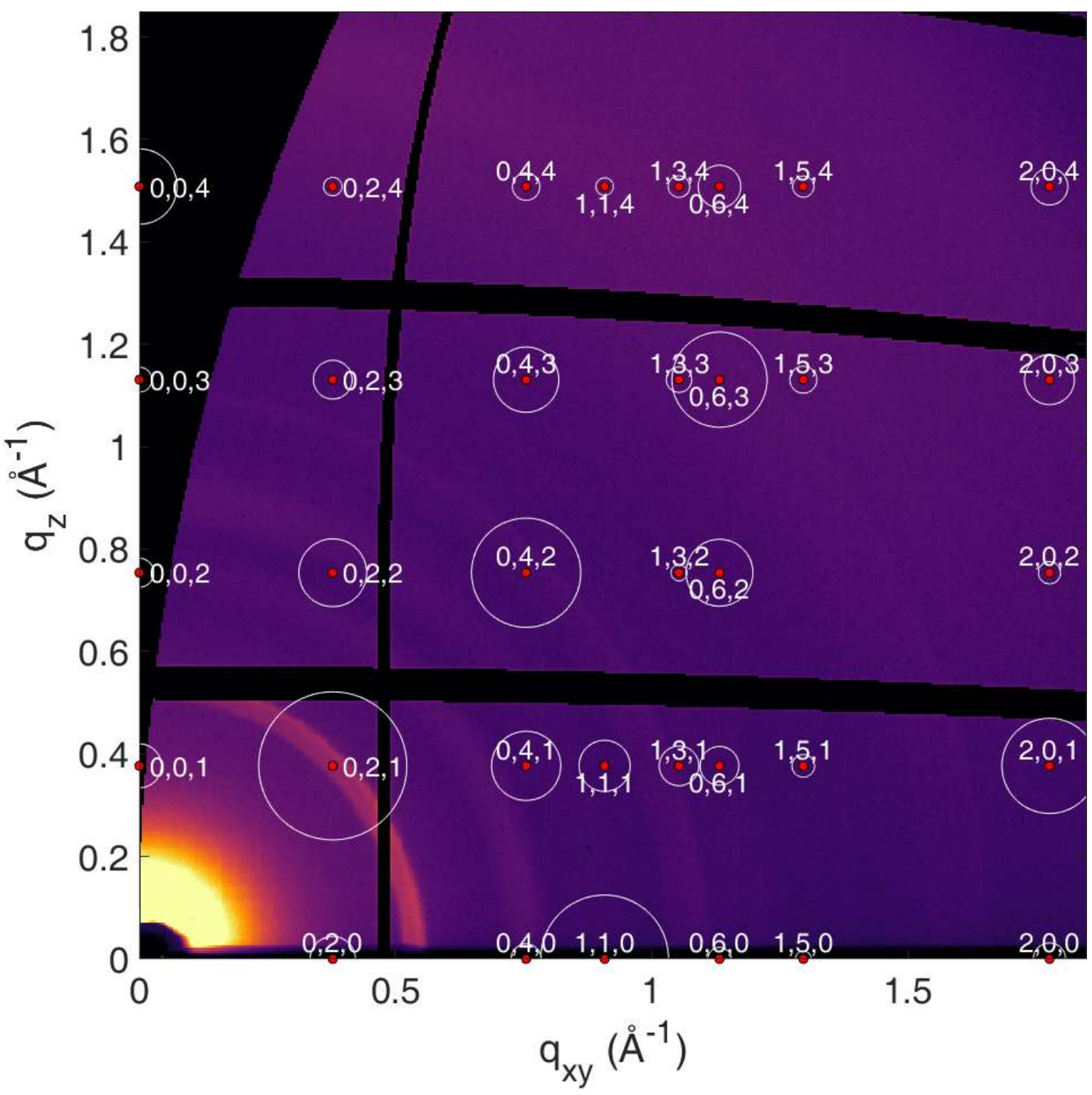

Figure S21: GIWAXS pattern of SURMOF-DMF-50.

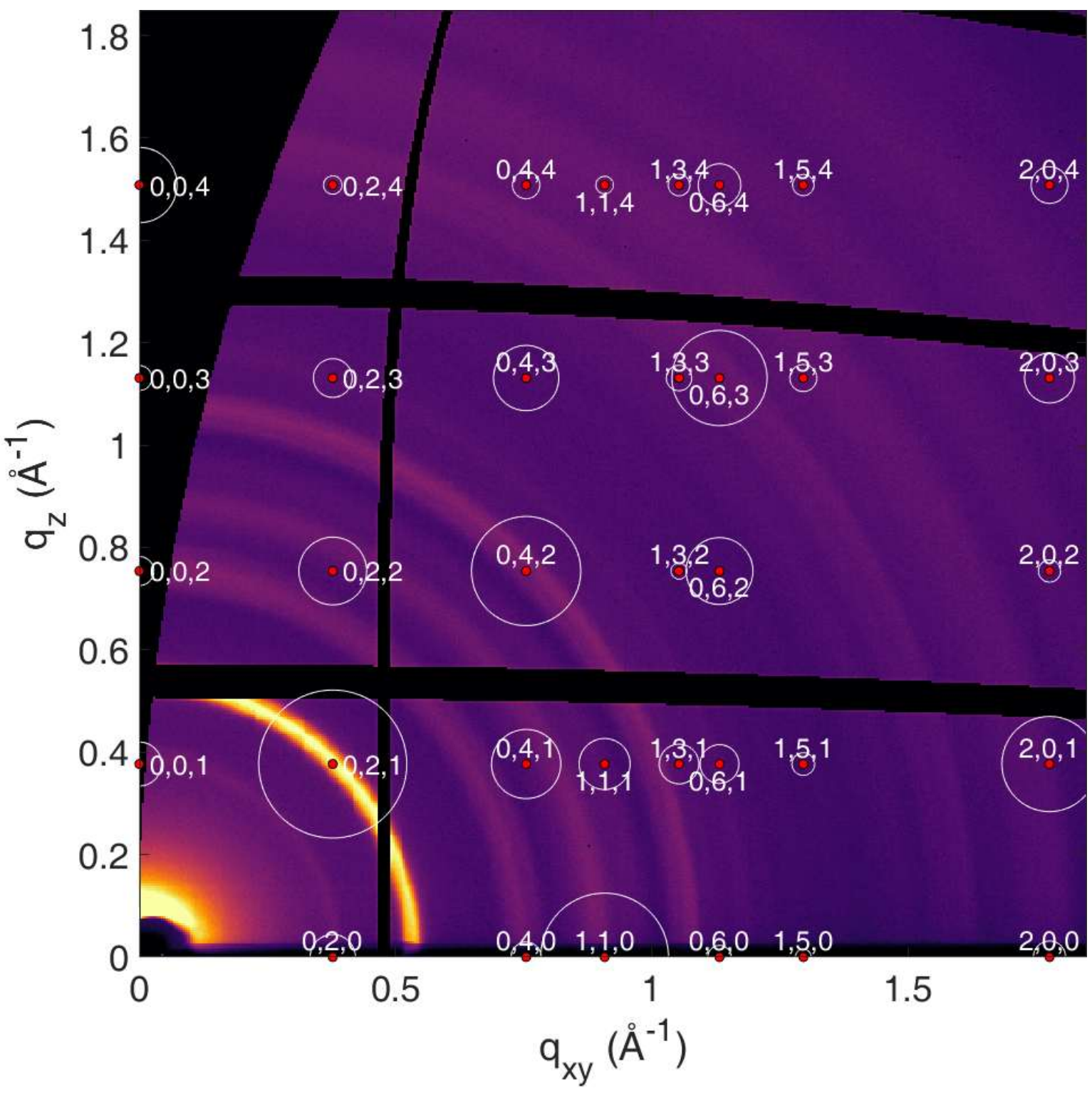

Figure S22: GIWAXS pattern of SURMOF-EtOH-38a.

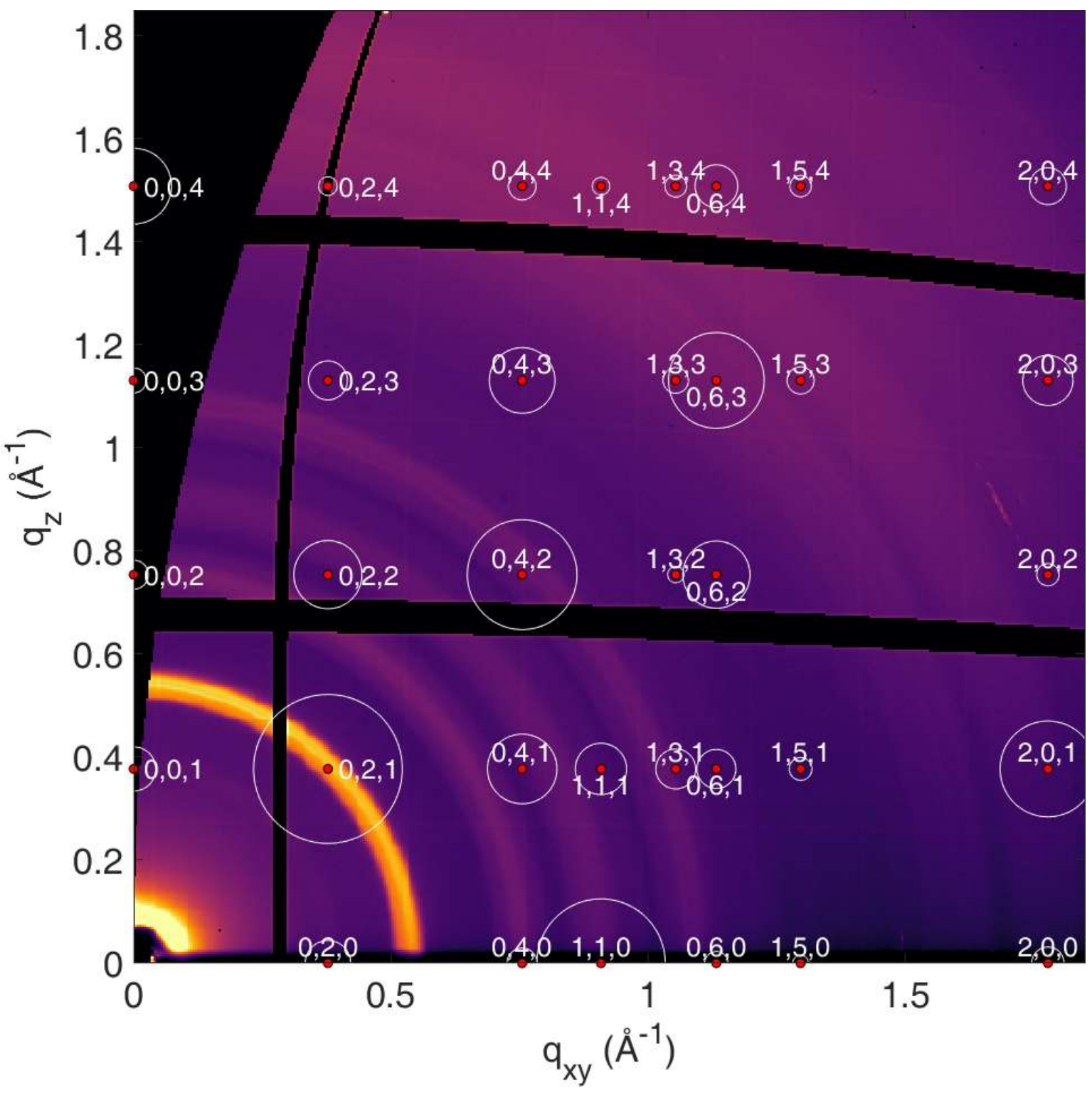

Figure S23: GIWAXS pattern of SURMOF-ETOH-38b.

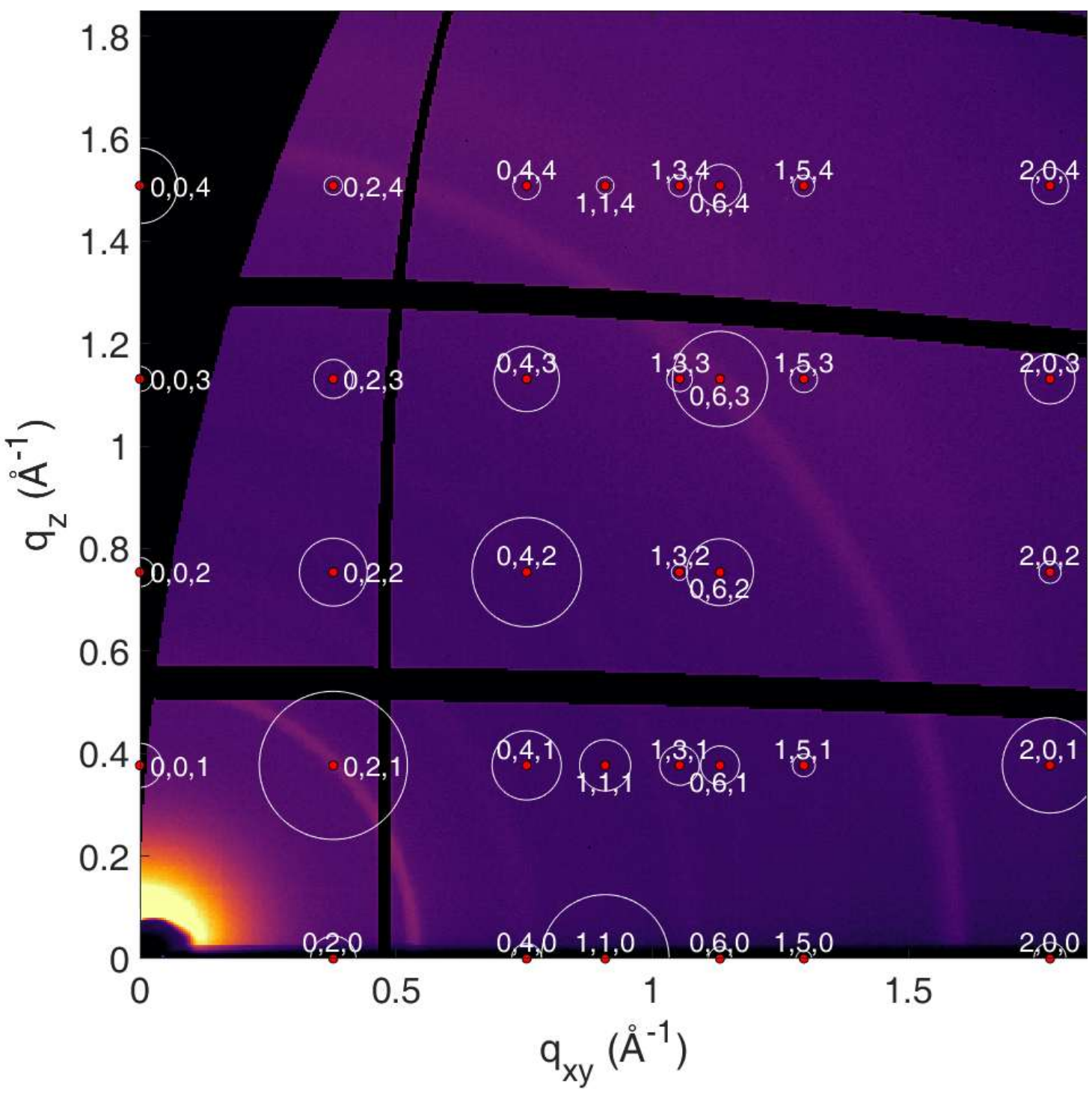

Figure S24: GIWAXS pattern of SURMOF-DMF-38a.

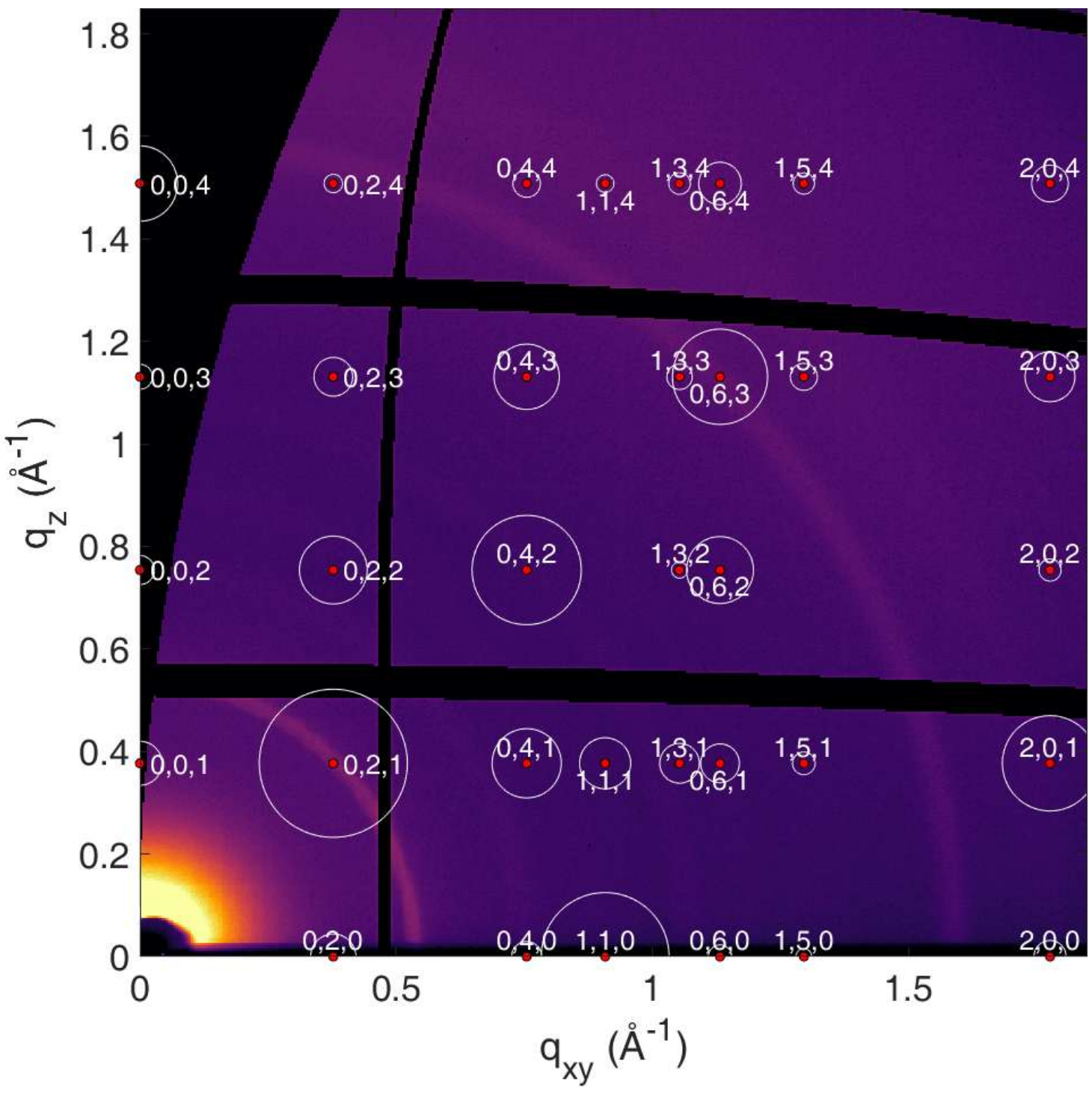

Figure S25: GIWAXS pattern of SURMOF-DMF-38b.

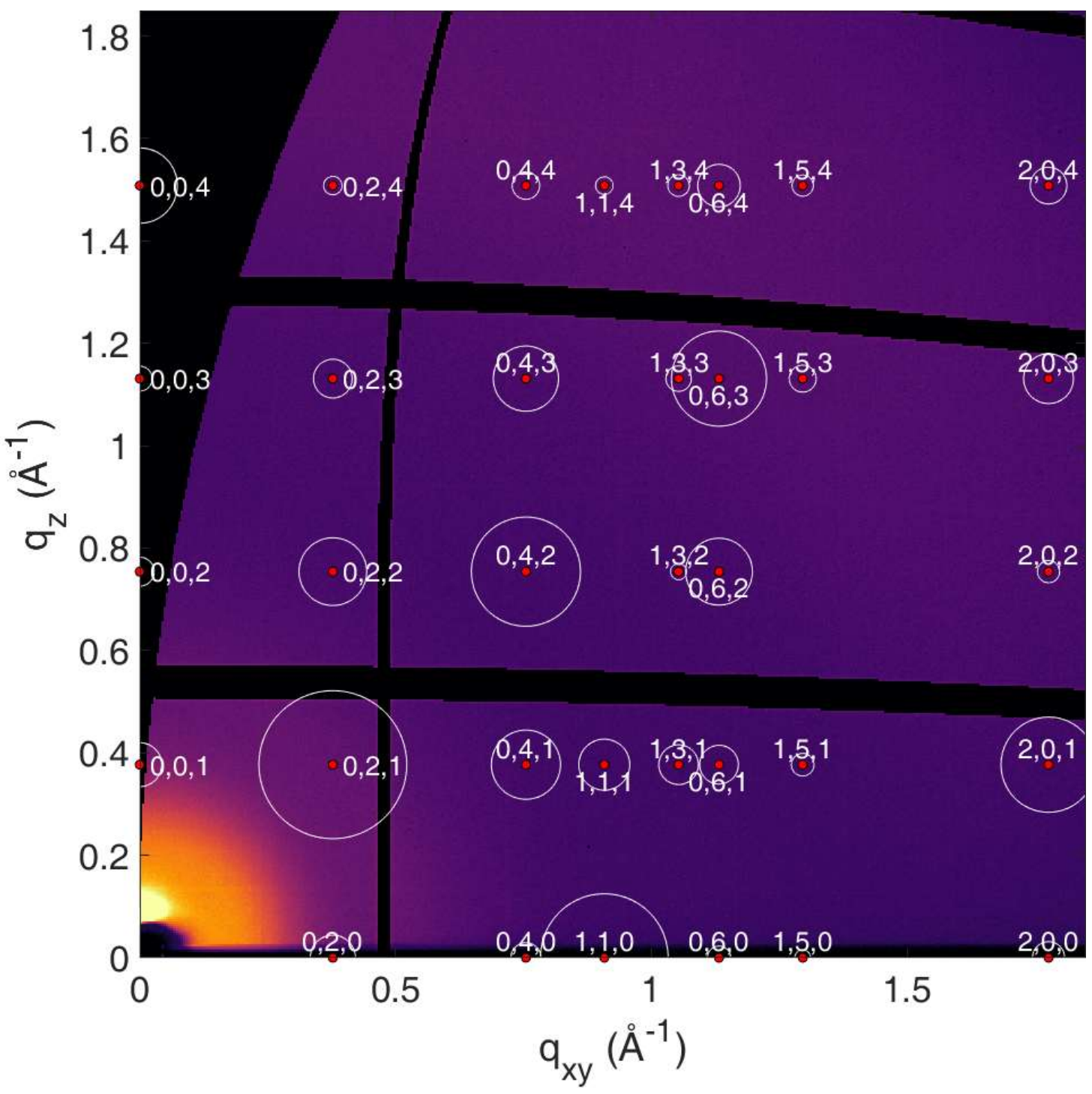

Figure S26: GIWAXS pattern of SURMOF-DMF-8F.

S4. Photophysical Properties and Oxygen Sensitivity of SURMOFs in Gas Phase

S3.1 Absorption and Emission Spectra of Dissolved Porphyrins

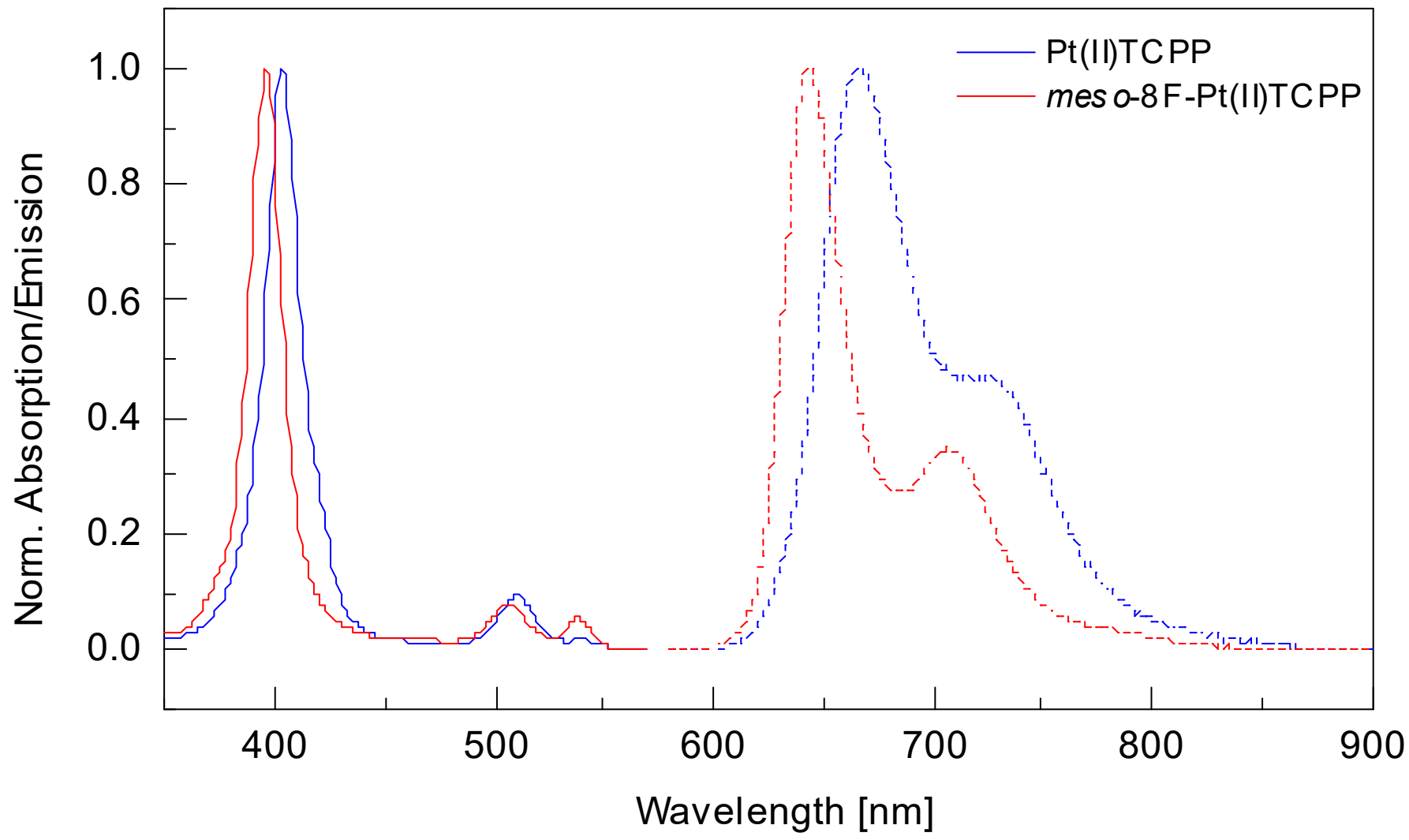

Figure S27: Normalized absorption and emission spectra. Blue lines: absorption (slid line) and emission (dotted line, λ_{exc} = 400 nm) of Pt(II)TCPP in DMF at 23 °C; red lines: absorption (solid line) and emission (dotted line, λ_{exc} = 392 nm) of 8F-meso-Pt(II)TCPP in DMF at 23 °C. The emission spectra were acquired in anoxic conditions.

S3.2 Absorption, Excitation and Emission Spectra of Porphyrin-based SURMOFs

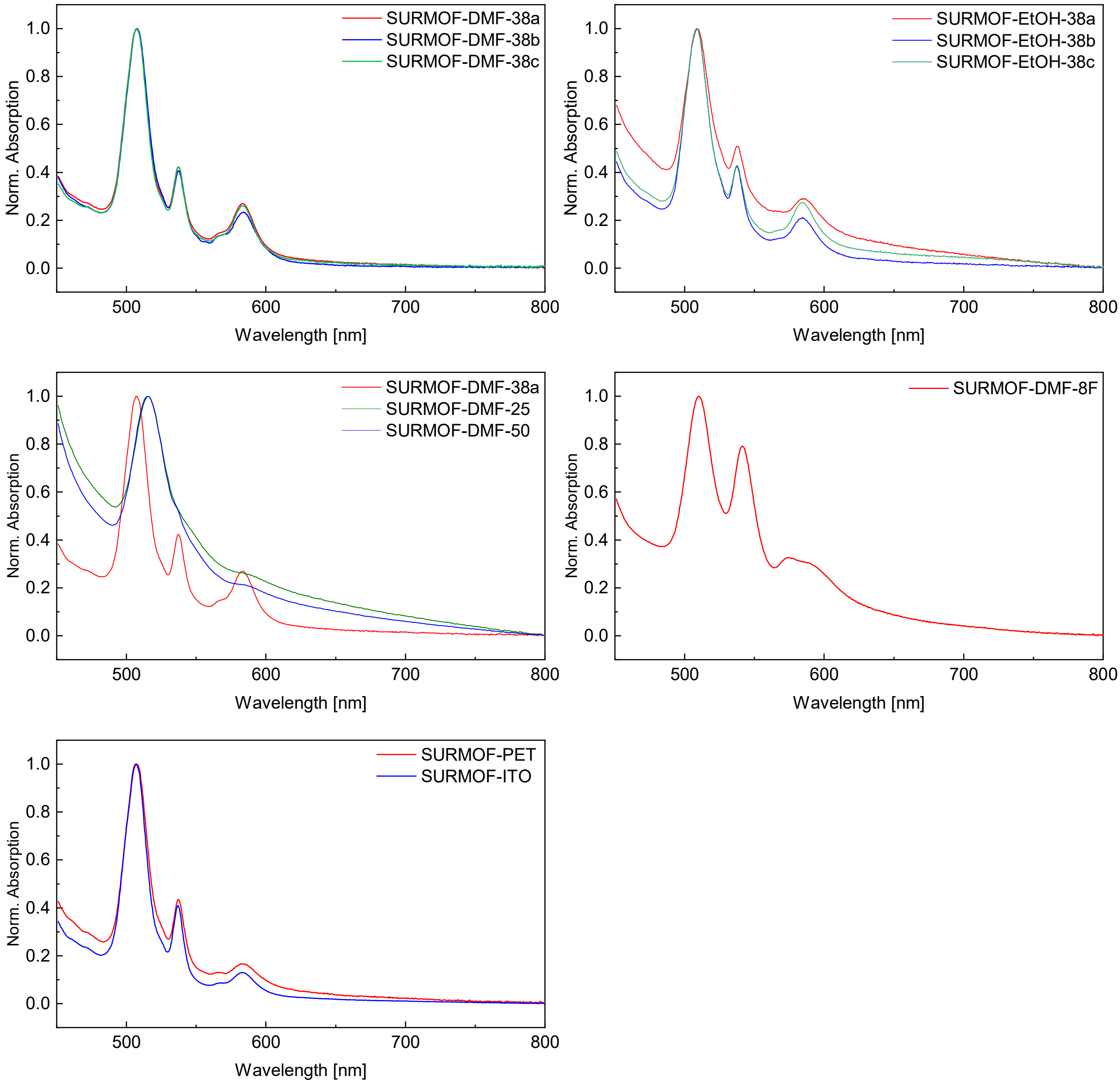

Figure S28: Normalized absorption spectra (of the Q-band region) of SURMOFs. Absorption spectra were not recorded immediately after fabrication.

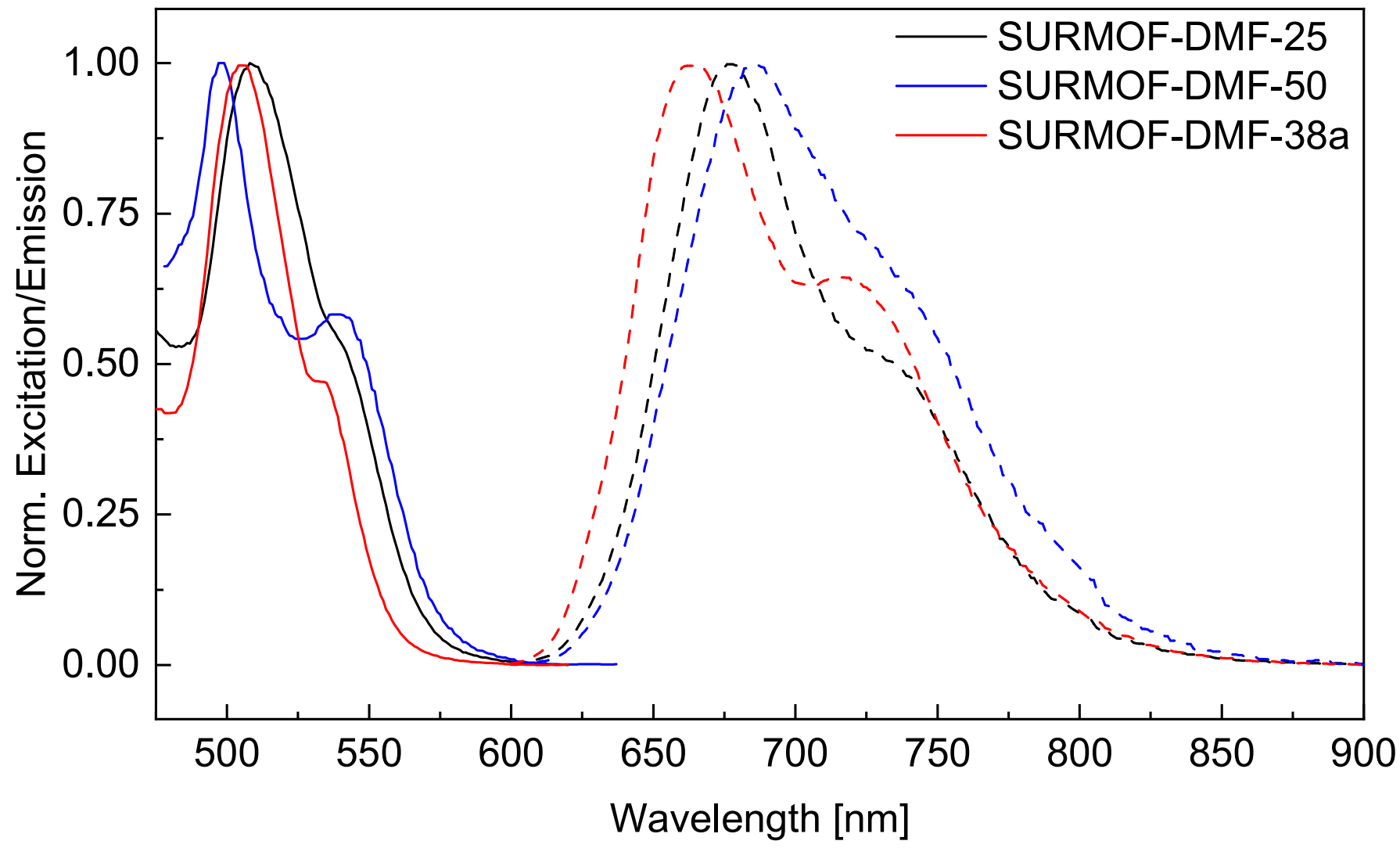

Figure S29: Excitation (solid lines; λ_{em} =660 nm) and emission (dashed lines; λ_{exc} =400 nm) spectra of Pt(II)TCPP based SURMOFs on glass substrates fabricated with different linker concentrations. Excitation and emission spectra were recorded in anoxic conditions at 23°C.

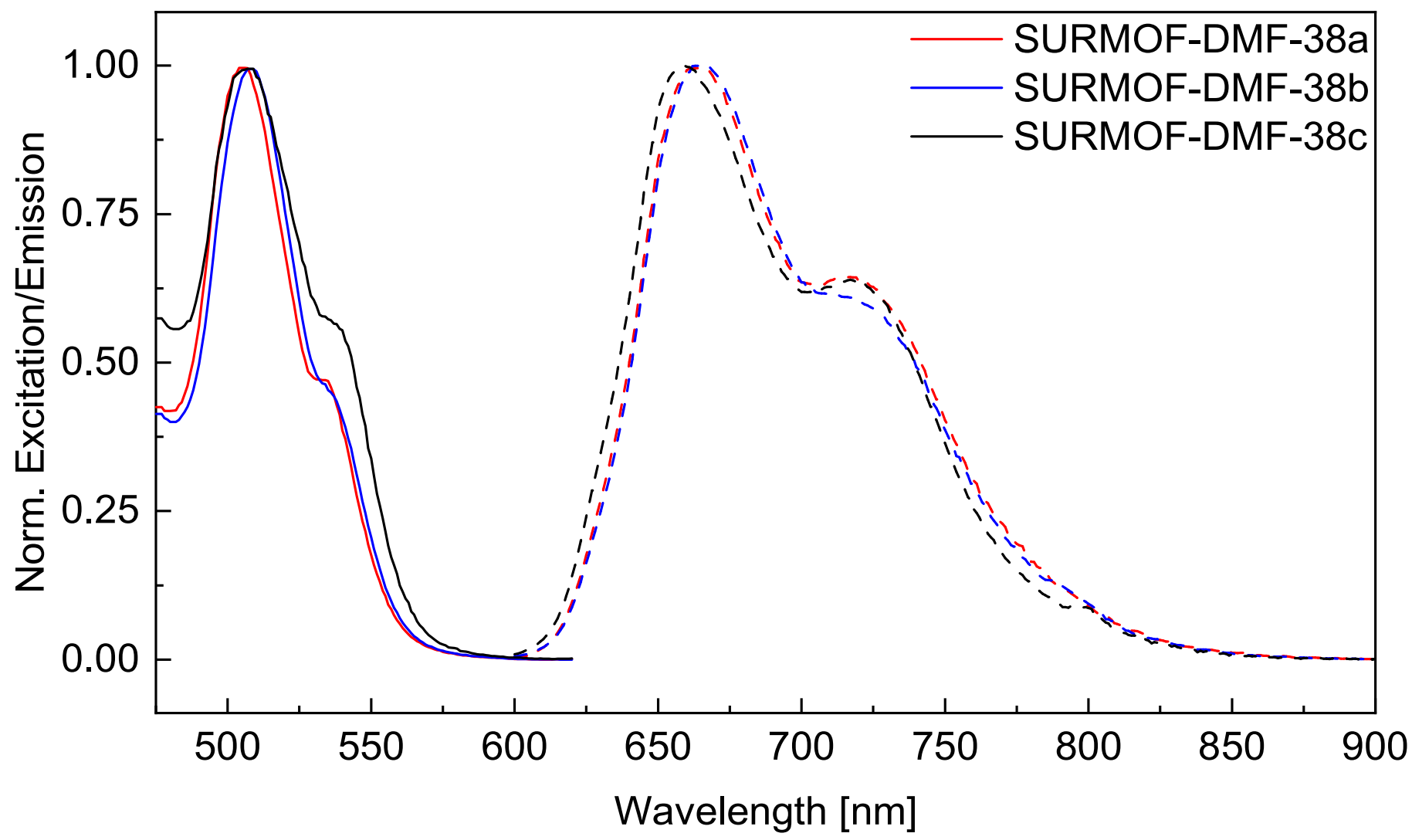

Figure S30: Excitation (solid lines; λ_{em} =660 nm) and emission (dashed lines; λ_{exc} =400 nm) spectra of Pt(II)TCPP based SURMOFs on glass substrates. Excitation and emission spectra were recorded in anoxic conditions at 23°C.

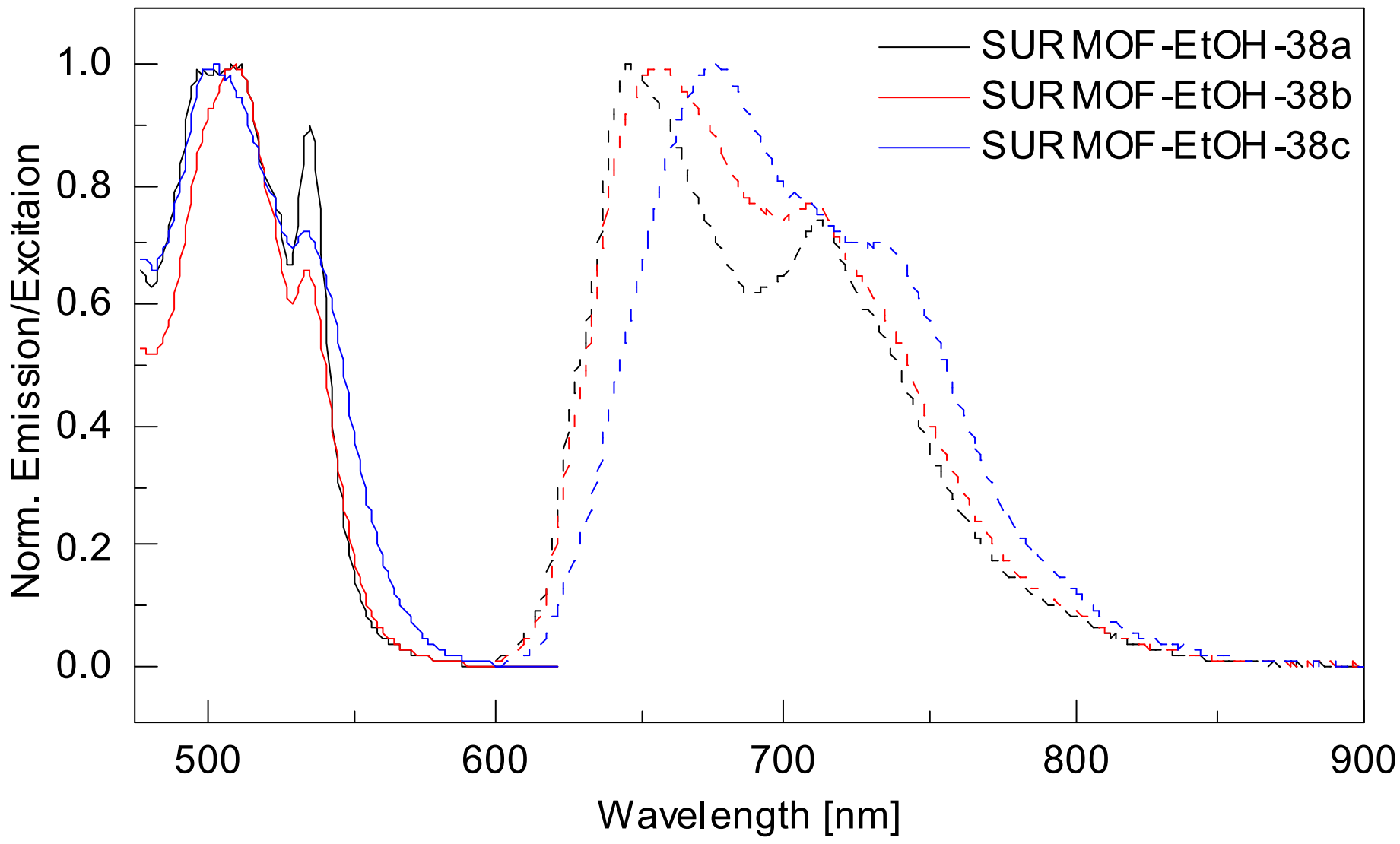

Figure S31: Excitation (solid lines; λ_{em} =660 nm) and emission (dashed lines; λ_{exc} =400 nm) spectra of Pt(II)TCPP based SURMOFs on glass substrates. Excitation and emission spectra were recorded in anoxic conditions at 23°C.

.

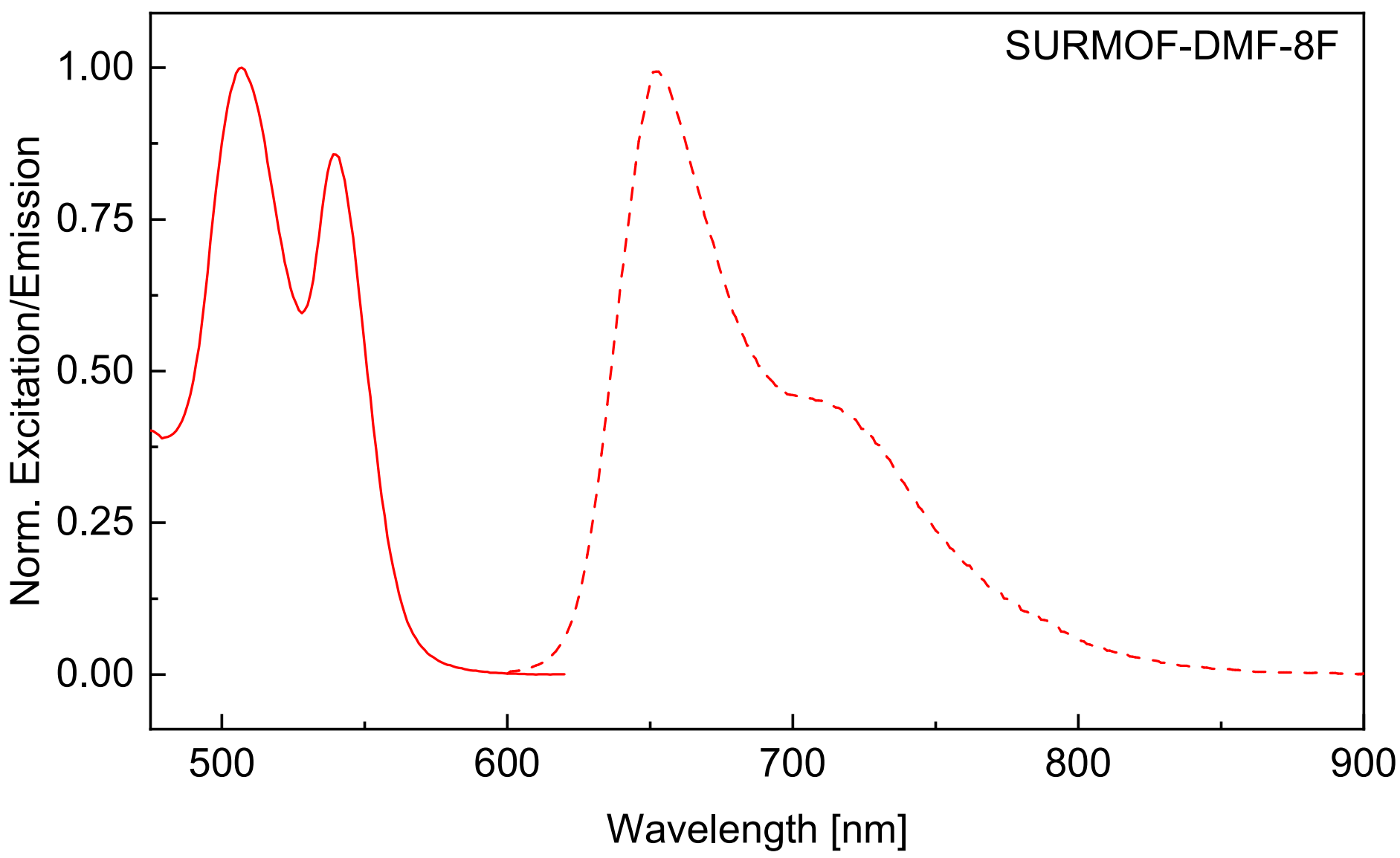

Figure S32: Excitation (solid line; λ_{em} =650 nm) and emission (dashed line; λ_{exc} =393nm) spectra of meso-8F-Pt(II)TCPP based SURMOF on glass substrates. Excitation and Emission spectra were recorded in anoxic conditions at 23°C .

S3.3 Luminescence Decays

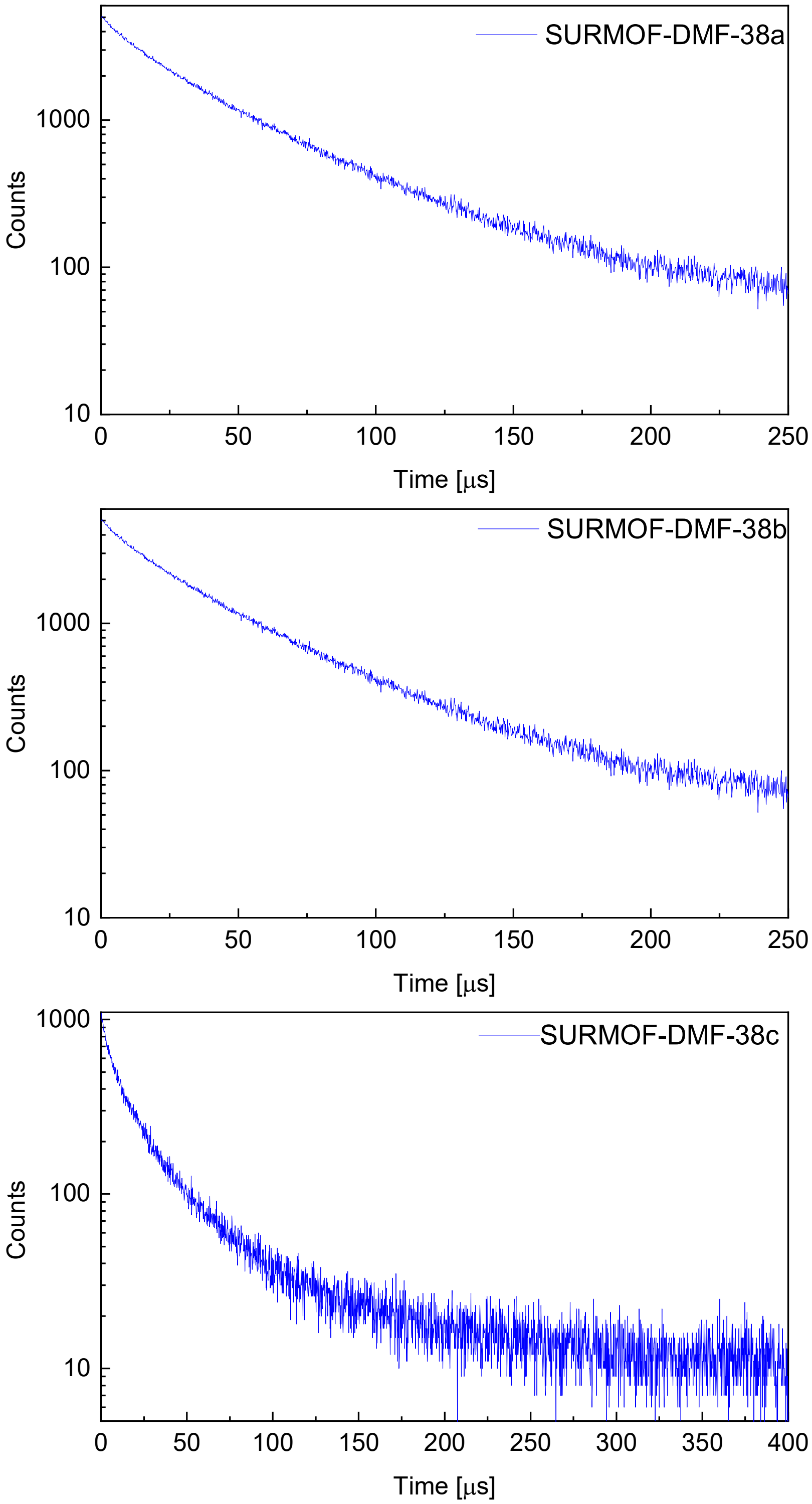

Figure S33: Luminescence decays of SURMOF-DMF-38X at room temperature in anoxic conditions. The decays were recorded at the emission maxima of the SURMOF.

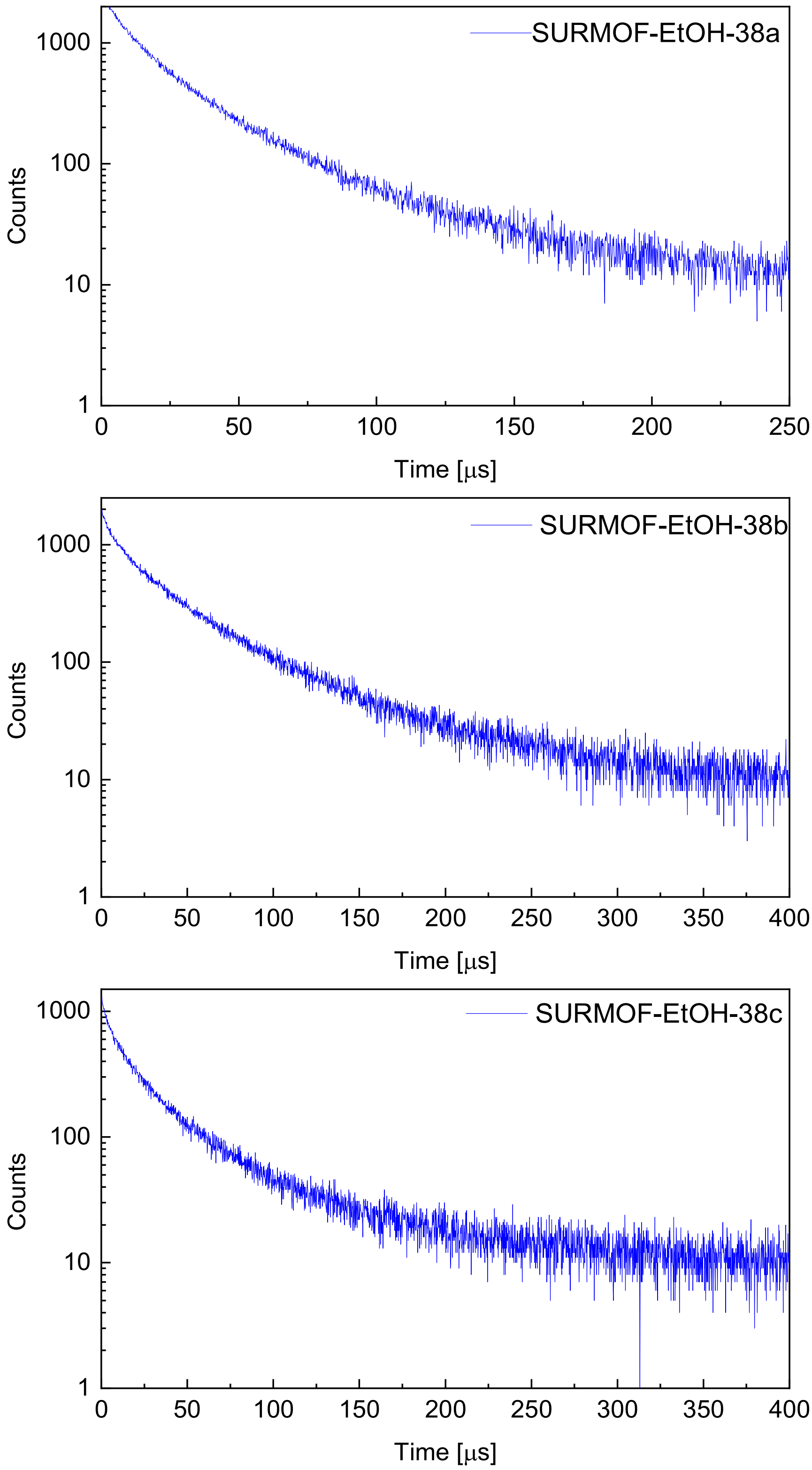

Figure S34: Luminescence decays of SURMOF-EtOH-38X at room temperature in anoxic conditions. The decays were recorded at the emission maxima of the SURMOF.

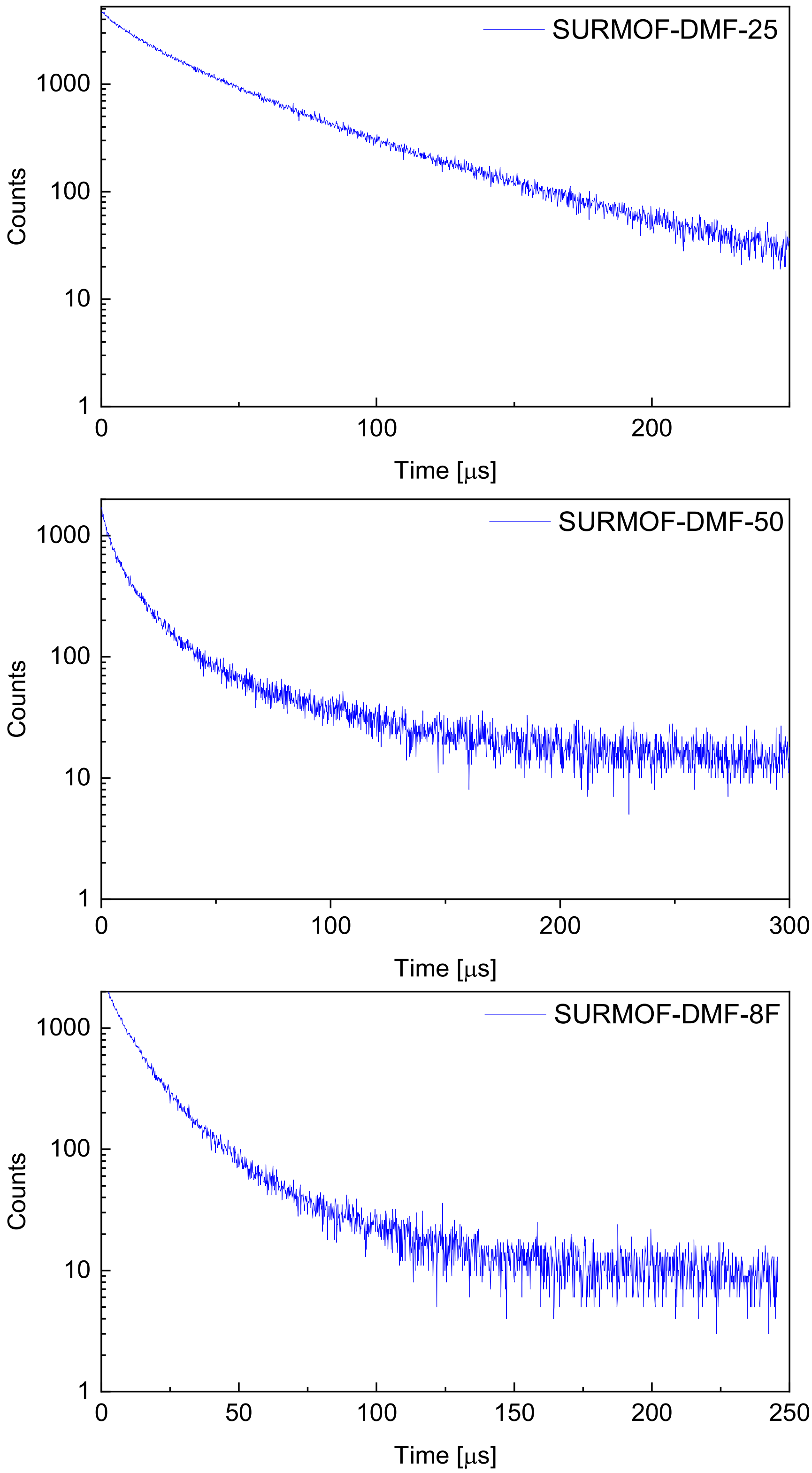

Figure S35: Luminescence decays of SURMOF-DMF-25, SURMOF-DMF-50 and SURMOF-DMF-8F at room temperature in anoxic conditions. The decays were recorded at the emission maxima of the SURMOF.

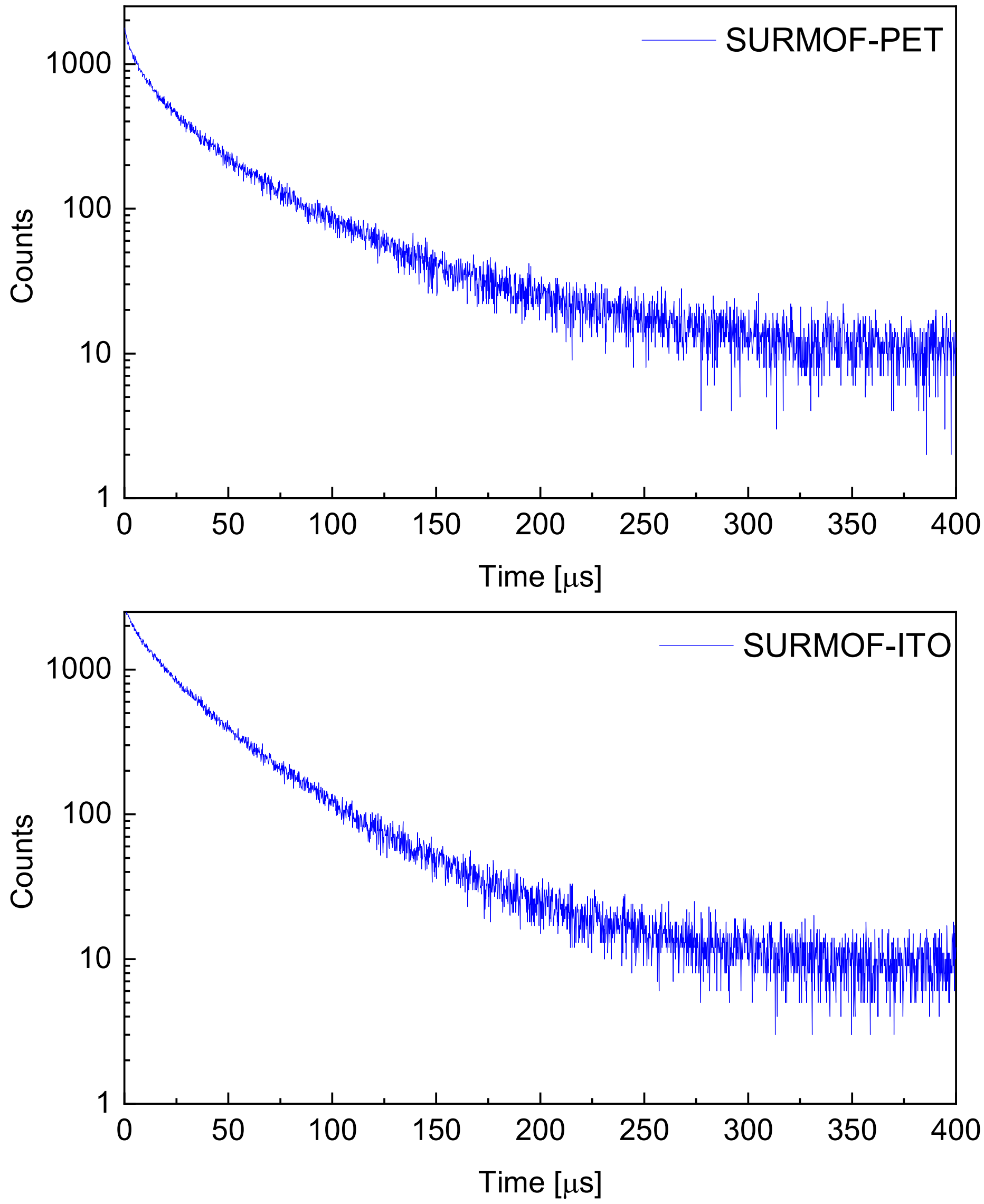

Figure S36: Luminescence decays of SURMOF-ITO and SURMOF-PET at room temperature in anoxic conditions. The decays were recorded at the emission maxima of the SURMOF.

S3.4 SURMOFs based on Partly Fluorinated Porphyrin

In addition, a second organic linker was tested for the fabrication of SURMOFs, *meso*-8F-Pt(II)TCPP, that bears 8 fluorine atoms in the *meso*-position of the porphyrin. SURMOF-DMF-8F was prepared similar compared to SURMOF-DMF-38X. Overall, the spectral properties of the SURMOF are rather similar to the ones obtained from the non-fluorinated linker (Figure S32). The position and relative intensity in the excitation and emission maxima are in line with the spectral properties of the linker in solution (Figure S27). As expected, the luminescence is quenched by oxygen (Figure S37).

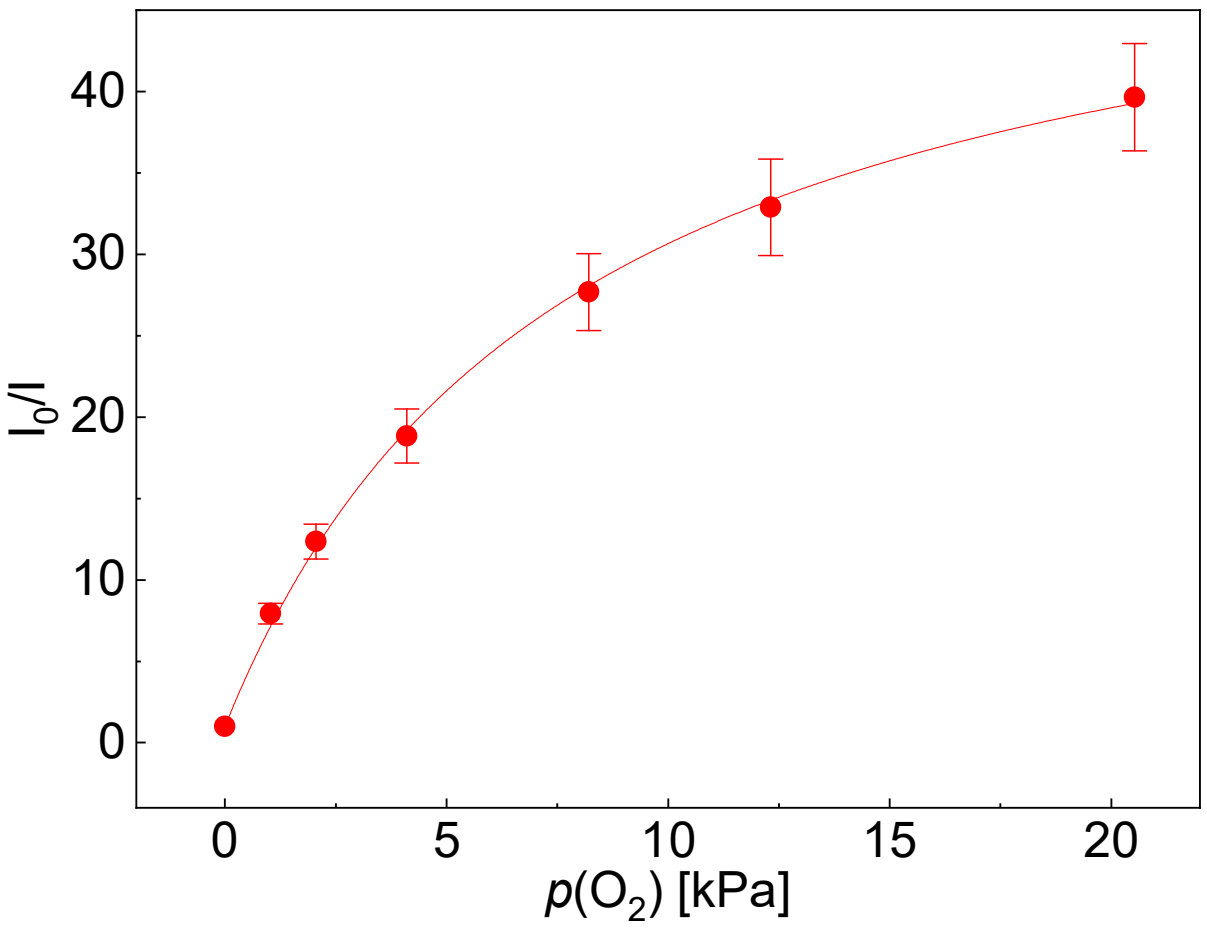

Figure S37: Stern-Volmer Plots of SURMOF-DMF-8F; intensity was recorded at the emission maximum (λ_{ex} =393 nm).

S3.5 Photophysical Properties and Oxygen Sensitivity of SURMOFs on Flexible Materials

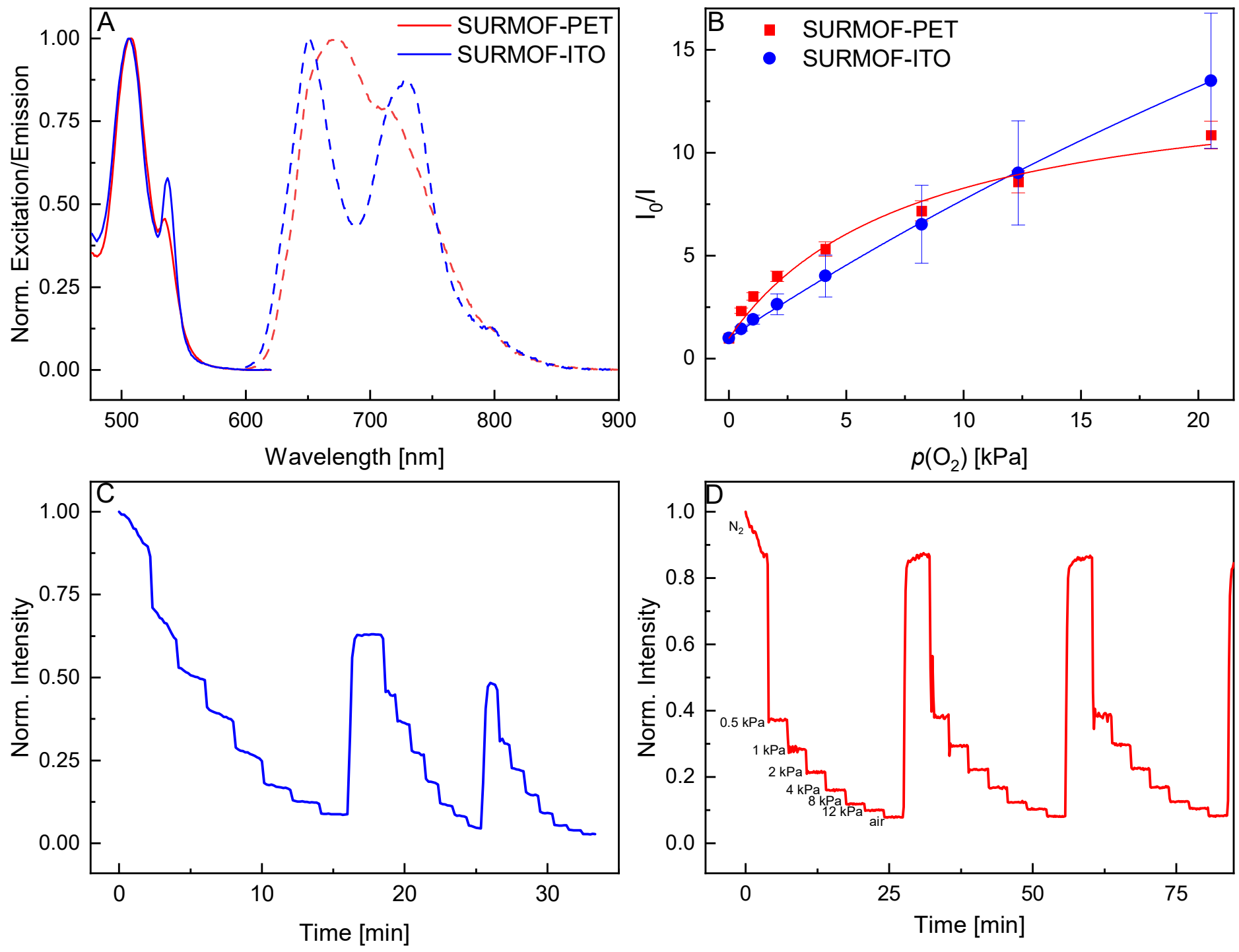

Figure S38: (A) Excitation (solid line; λ_{em} =650 nm) and emission (dashed line; λ_{em} =400 nm) spectra of Pt(II)TCPP/In-Oxo-Cluster based SURMOF on flexible supports. (B) Stern Volmer Plot of SURMOF-DMF on flexible supports; intensity was recorded at the emission maxima (λ_{ex} = 400 nm). (C) Dynamic response of SURMOF-ITO to different partial pressures of oxygen. (D) Dynamic response of SURMOF-PET to different partial pressures of oxygen (N_2, 0.5 kPa, 1.0 kPa, 2.1 kPa, 4.1 kPa, 8.2 kPa, 12.3 kPa, air).

S3.6. Photophysical Properties and Oxygen Sensitivity of SURMOFs in Toluene

For the characterization of SURMOFs in toluene two new batches of SURMOFs were synthesized following the procedure for SURMOF-DMF-38X and SURMOF-EtOH-38X, respectively. Photophysical and oxygen sensing properties of SURMOFs in organic solvents were acquired in a 2 cm x 1 cm x 5.5 cm quartz cuvette. The SURMOFs were cut to the appropriate size and positioned on the front of the cuvette with the coated side facing inwards. The cuvette was filled with the solvent and closed with a self-build lid made of Teflon. The environment was controlled with a constant stream of gas (40 mL min^{-1}) bubbled through the solvent (schematic illustration in Figure S39). The gas composition was controlled with two mass-flow controllers using compressed air and nitrogen. Prior to the cuvette, the gas was passed through an additional flask containing the respective solvent in order to prevent the solvent evaporation during the measurements.

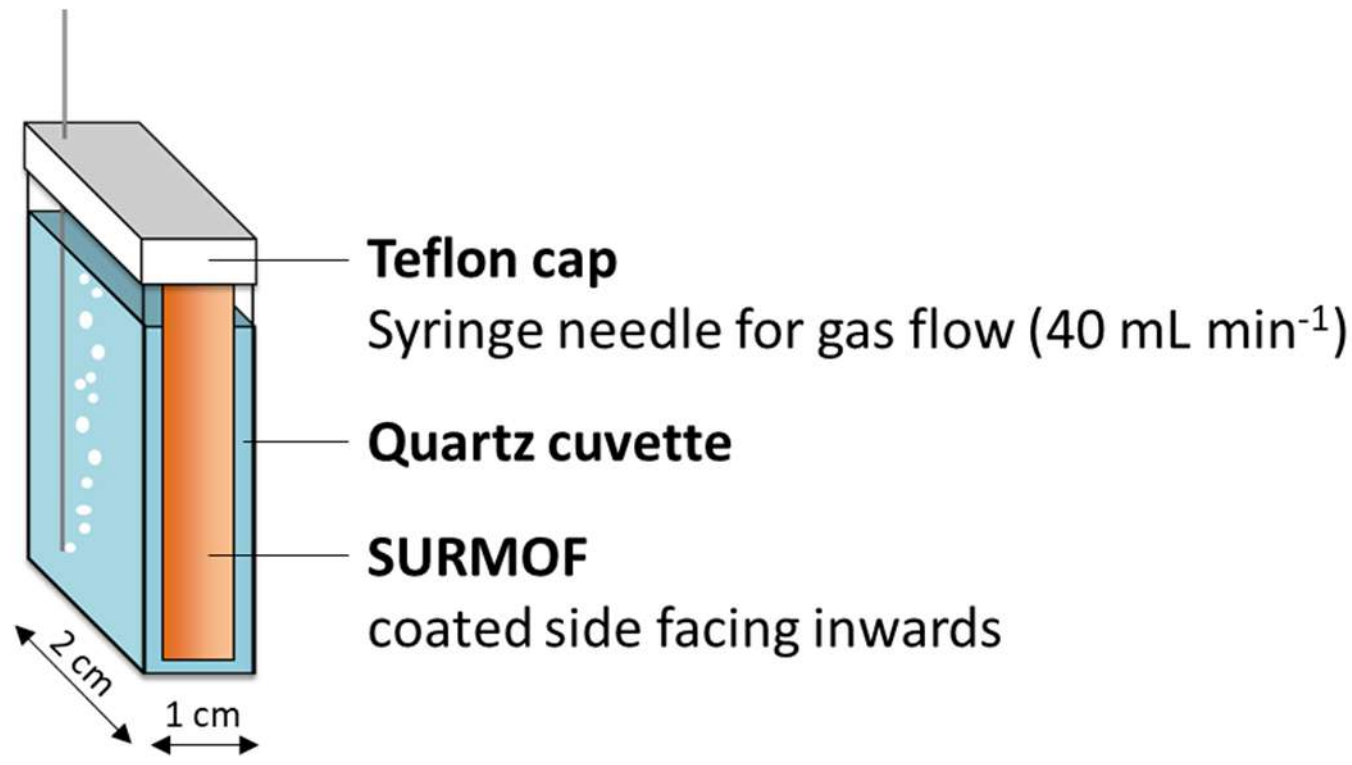

Figure S39: Schematic representation of the measuring cell. The SURMOF is positioned at the front of the quartz cuvette, then closed with a teflon lid. The oxygen content is controlled via the gas flow through the measuring cell.

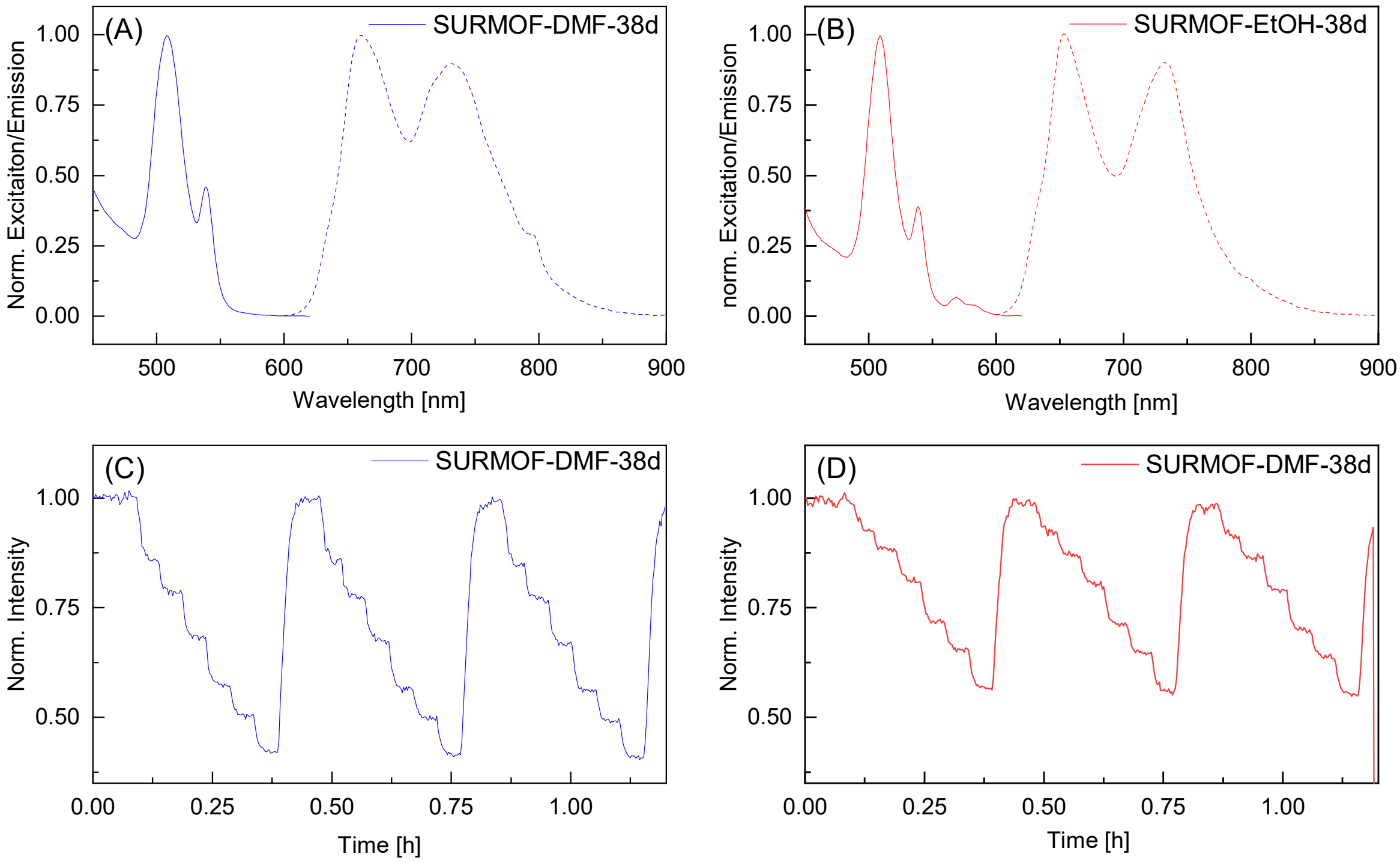

Figure S40: Photophysical properties of SURMOF-DMF-38d (A and C) and SURMOF-EtOH-38d (B, and D) in toluene. A and B: Excitation spectra (solid line; λ_{em} =660 nm) and emission spectra (dashed line; λ_{exc} =400 nm) of the SURMOF in toluene recorded under anoxic conditions; C and D: Phosphorescence response of SURMOFs recorded in toluene at 20 °C to alternating oxygen concentrations; intensity was monitored at emission maximum; (λ_{ex} = 400 nm)

Table S2: Oxygen sensing properties of SURMOFs in toluene.

**Sample**	**I_0/I**	**K_{SV1} [kPa^{-1}]**	**f**	**Intensity loss after 3 h [%]**
SURMOF-DMF-38d	2.38 ± 0.01	0.18	0.73	**45**
SURMOF-EtOH-38d	1.75 ± 0.01	0.095	0.65	**47**

S3.7. Photodegradation of SURMOFs in Gas Phase and Toluene

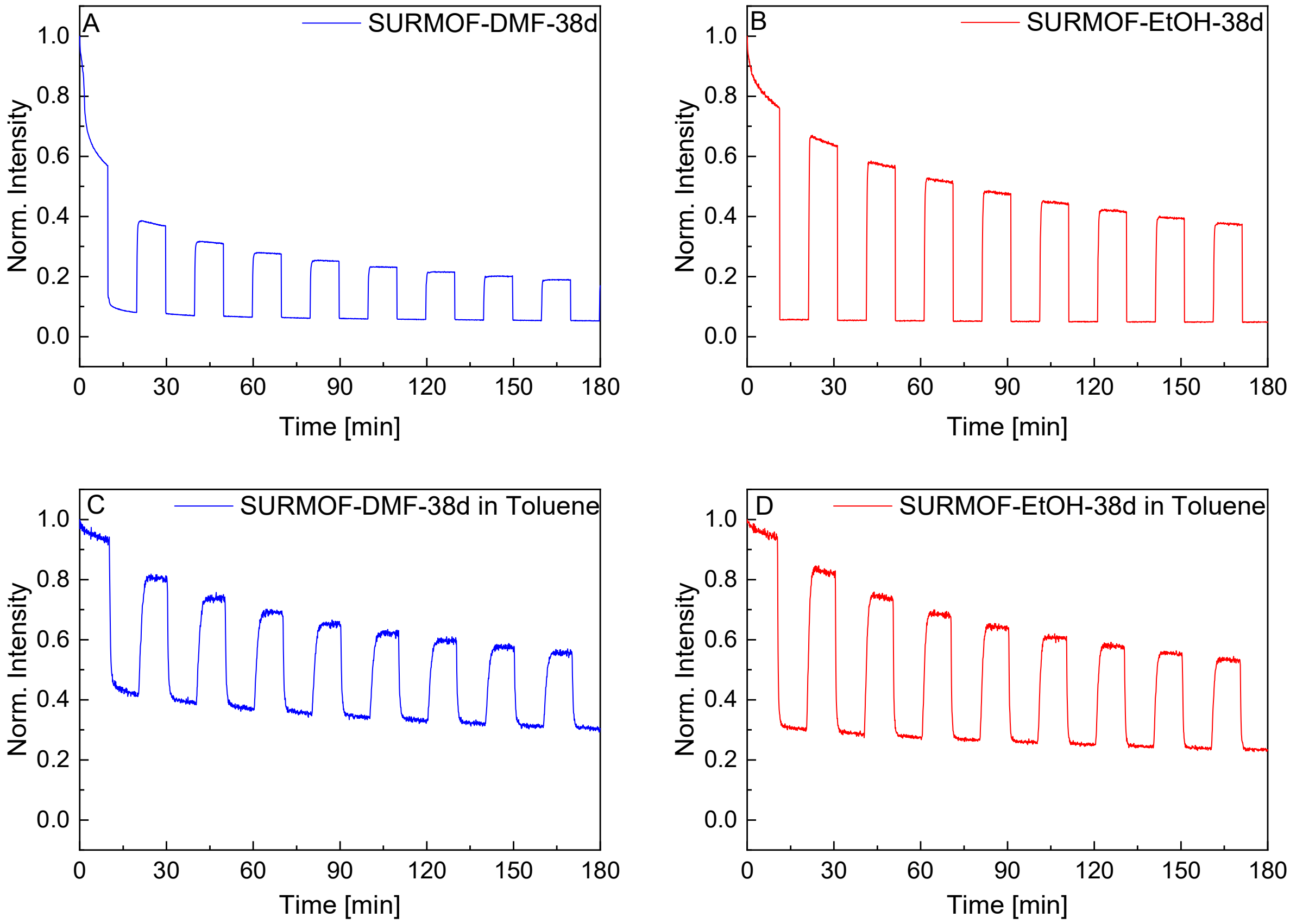

*Figure S41: Photodegradation of SURMOF in toluene (A and B) and in the gas phase (C and D) under continuous illumination with a Xe lamp at the maximum of the Soret band for three hours, monitored in the emission maximum (λ_{ex} = 400 nm). The environment was controlled via an alternating gas stream of nitrogen (10 min) and air (10 min) through. *After the fabrication process, SURMOF-DMF-38d and SURMOF-EtOH-38d were cut in half, to measure the photodegradation process in the gas phase and in organic solvent independently.*

S3.8 Activation and Sensitivity Loss

Regarding powder MOFs, activation is normally performed to remove uncoordinated building blocks, remaining solvents and guest molecules from the network to achieve the highest activity. Typically, no activation step is performed for SURMOFs. Two new SURMOF samples were fabricated with the same parameters as SURMOF-DMF-38X on glass, further referred as SURMOF-DMF-38e and SURMOF-DMF-38f.

The SURMOF-DMF-38e was characterized (Figure S42, SURMOF-DMF-38e-fresh), afterwards dried under vacuum for 7 days and characterized again (Figure S42, SURMOF-DMF-38e-act). The MOF demonstrates only minor changes in the absorption spectrum, but the emission intensity and luminescence quenching by oxygen decrease drastically after the activation step. The emission spectrum of SURMOF-DMF-38e-act shows an additional shoulder at ~ 720 nm. A new absorption band becomes visible an ~ 584 nm after the drying process (Figure S42 A). This correlates with the absorption spectra of Pt(II)TCPP in DMF during photodegradation experiments, where an additional band becomes visible at the same wavelength after continuous illumination for 3 hours.[2] Excitation of the SURMOF-DMF-38e-act in the new absorption band reveals weak emission in the far-red region of the electromagnetic spectrum at ~745 nm, which aligns to the shoulder of SURMOF-DMF-38e-dry. Potential formation of the reduced porphyrin in very minor amounts, however, does not explain drastic changes in the emission of the SURMOF.

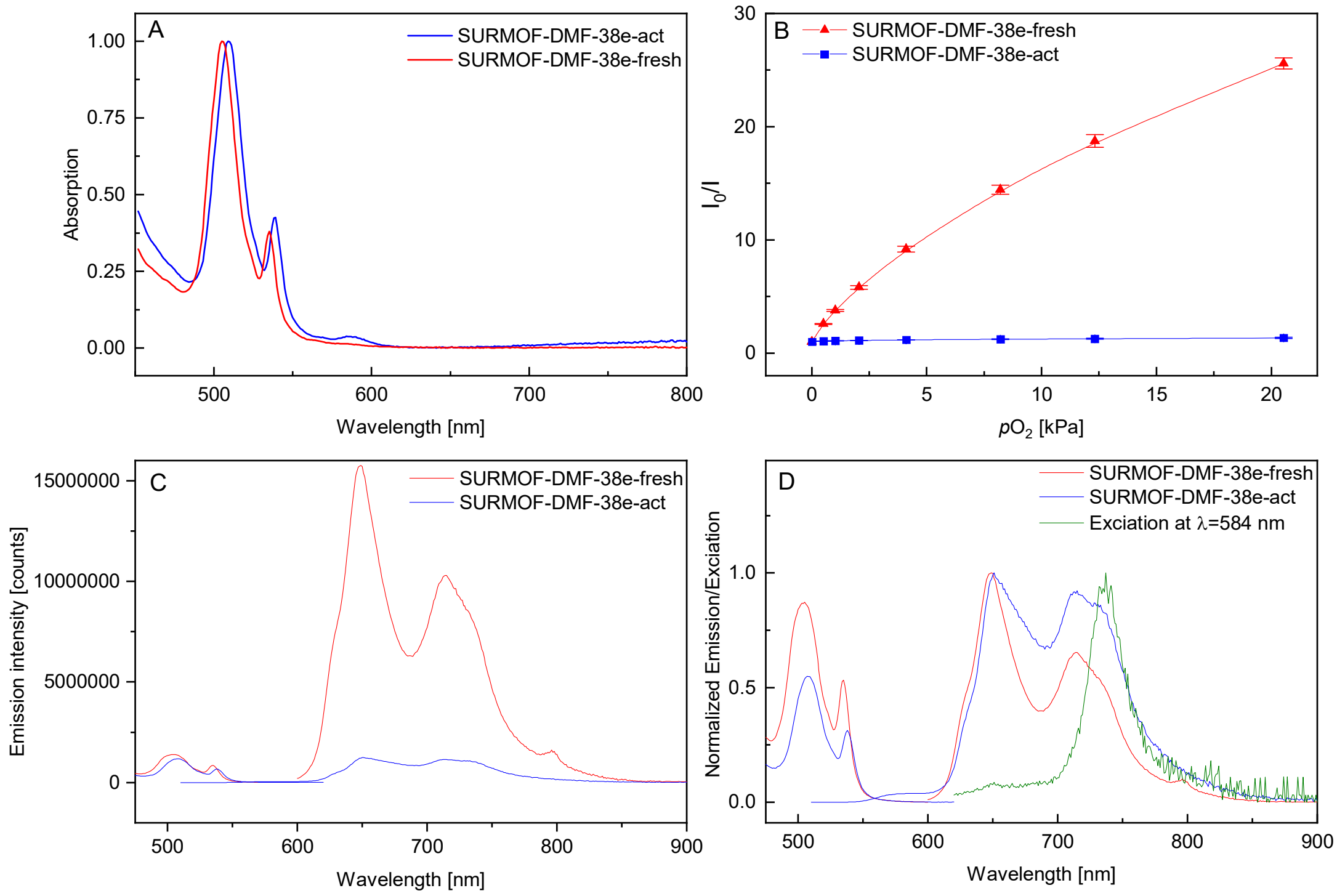

Figure S42: Photophysical characterization of SURMOF-DMF-38e directly after the synthesis and after drying under vacuum for 7 days. (A) Normalized absorption spectrum of the SURMOF of the Q-band region. (B) Stern-Volmer plot of SURMOF-DMF-38e. The intensity was monitored at the emission maximum (λ_{ex} = 400 nm) (C) Excitation (λ_{em} =650 nm) and emission spectra (λ_{exc} =400 nm) before and after the activation step with the same measurement parameters. (D) Normalized excitation and emission spectra of SURMOF-DMF-38e (red and blue line). Green line: emission monitored by excitation at the additional absorption band after drying (λ_{exc}= 584 nm).

In order to investigate the behaviour in more detail we recorded the absorption spectra of SURMOF-DMF-38-f directly after the fabrication und during the activation process (Figure S43). Therefore, the sample was dried under vacuum for three days (Figure S43, 72 h), followed by activation under vacuum at elevated temperature (45 °C) for two days (Figure S43, 96 h and 120 h respectively).

During the activation, we observe bathochromic shift in the position of the Q-band and increase of the scattering background, which may indicate the change in the polarity of the porphyrin environment in the framework. Additionally, a minor band becomes visible between 580 nm and 600 nm, similar to SURMOF-DMF-38e-act.

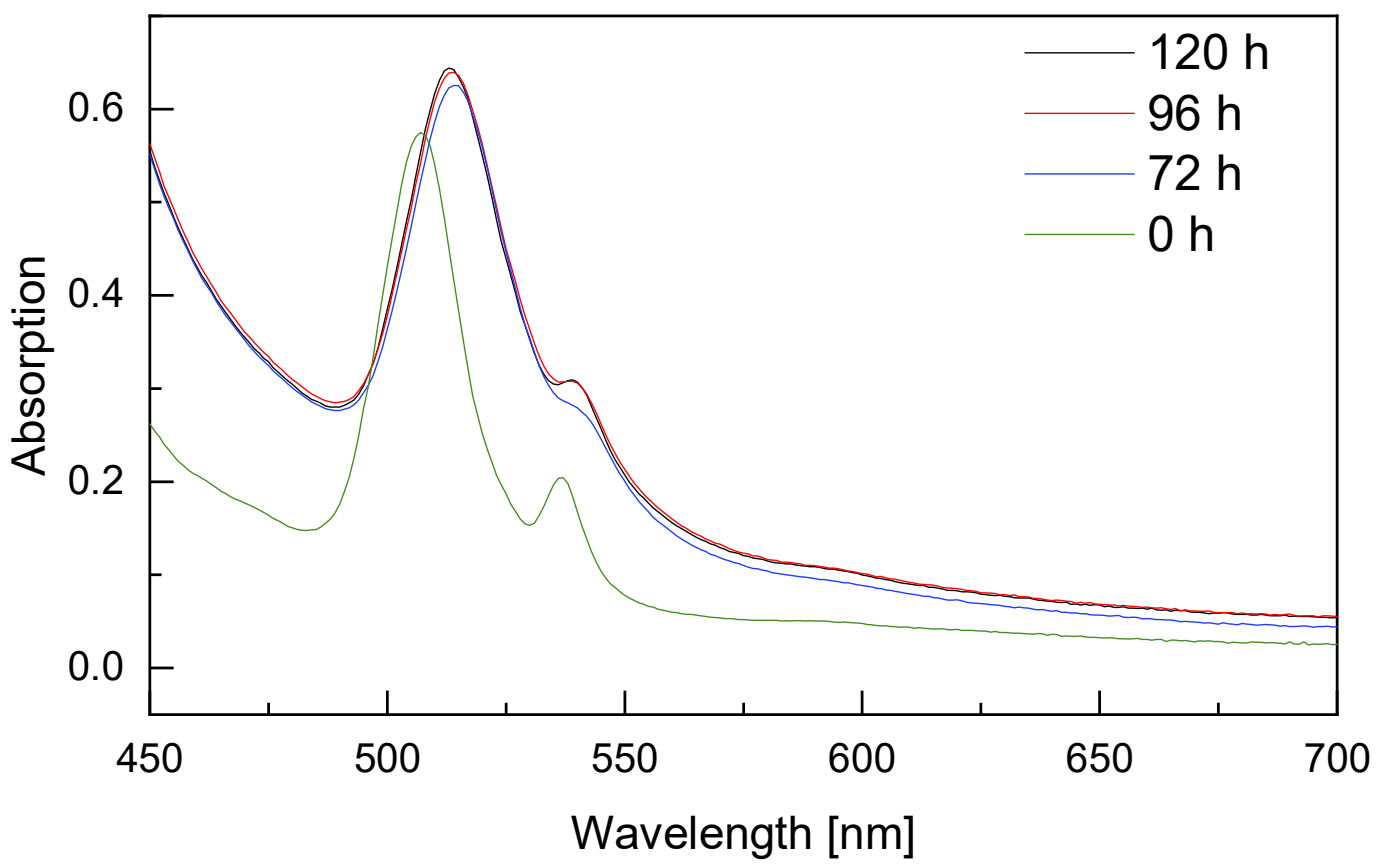

Figure S43: Absorption spectra of the Q-band region SURMOF-DMF-38f *during the activation process.*

One of the earlier samples of the SURMOF prepared from DMF solution in preliminary experiments and investigated with GIWAX showed no significant change in the diffraction pattern after the activation (Figure S44) which suggests that the drastic changes in the photophysical properties are not associated with alteration of the crystal structure or complete collapse of the framework during vacuum treatment.

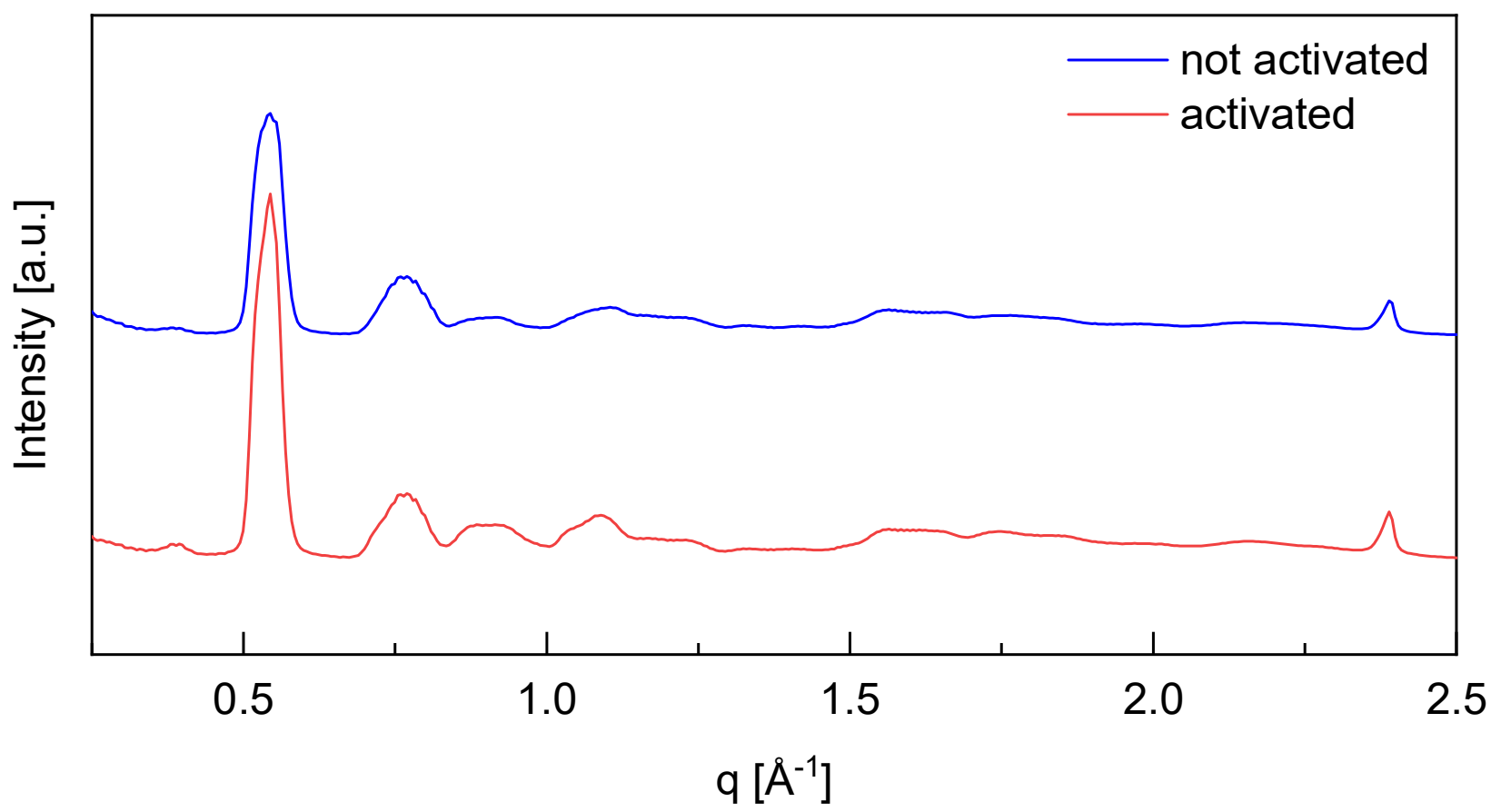

Figure S44: GWAX plots of porphyrin based SURMOF prepared from DMF solution in preliminary experiments before and after drying process.

S5. In-Pt(II)TCPP powder MOFs

S5.1 Synthesis and Activation of In-Pt(II)TCPP powder MOF

In-Pt(II)TCPP MOF was synthesized analogously to the literature procedure reported for the In-MOF based on the free-base porphyrin.[3] Briefly, 11.7 mg Pt(II)TCPP (11.8 µmol, 1 eq.) and 10.54 mg $InCl_3$ (47.6 µmol, 4 eq.) were dissolved in 2 mL DMF for 20 minutes in an ultrasonic bath. Afterwards, 300 µL HNO_3 were added dropwise. The vial was sealed and placed in a preheated oven for 2 days at 100 °C. The resulting crystals were collected by centrifugation and washed three times with DMF and three times with acetone. Solvent exchange was performed by dispersing the crystals in acetone followed by evaporation on a heating plate operated at 80 °C. Finally, the obtained crystals were dried under vacuum at 120 °C for 72 h. Yield: 7.83 mg, 53.5 % (empirical formula: $C_{48}H_{24}In_2N_4O_{10}Pt$), as an orange powder.

X-ray diffraction measurement of the powder sample was performed on an Empyrean powder diffractometer from Malvern Panalytical (www.malvernpanalytical.com) using Cu-Kα radiation.

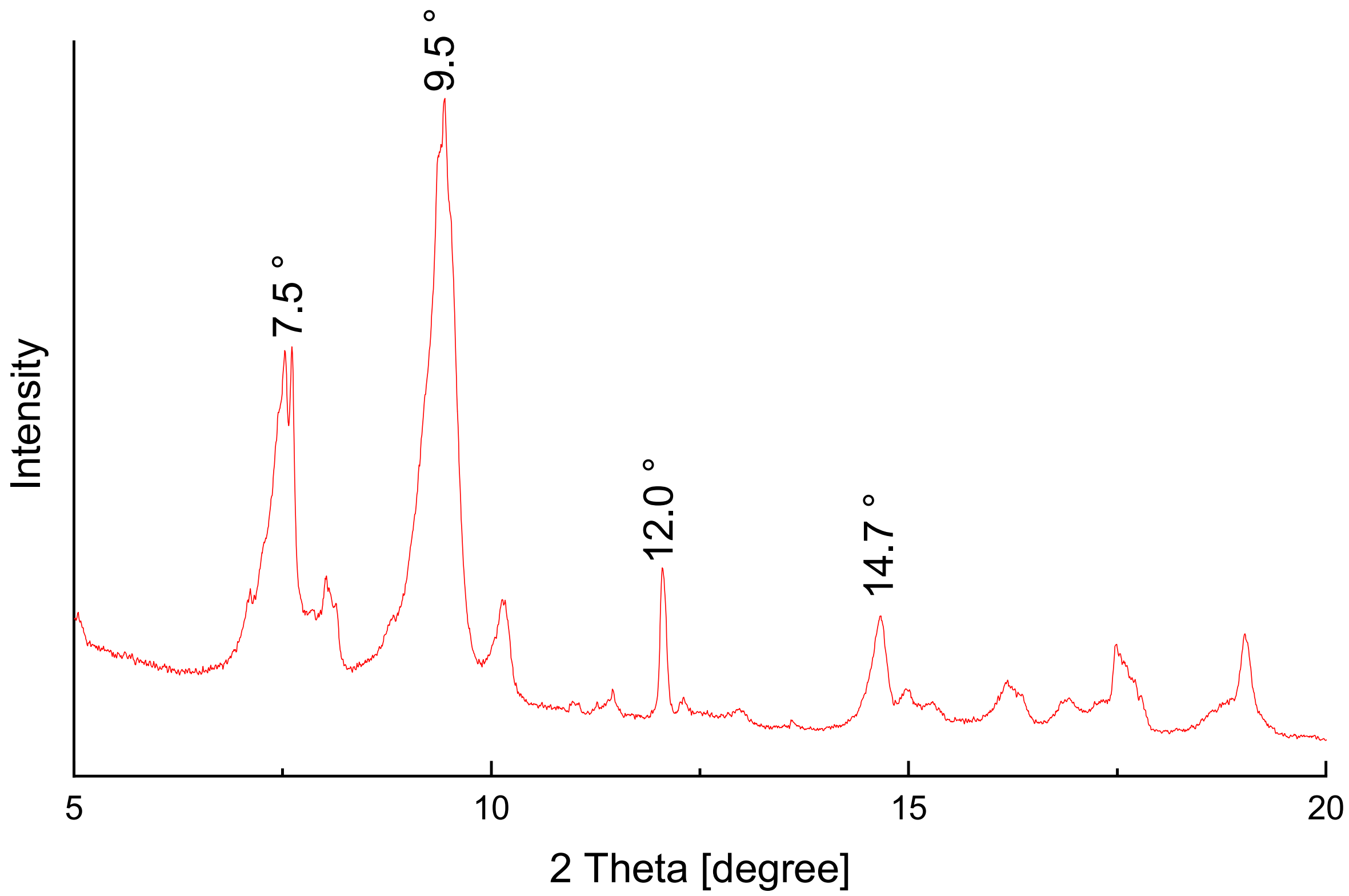

Figure S45: XRD of the powder MOF sample using Pt(II)TCPP and $InCl_3$ as building blocks.

S5.2 Photophysical and Oxygen Sensing Properties of In-Pt(II)TCPP powder MOF in the Gas Phase

Excitation and emission spectra of In-Pt(II)TCPP based MOF are shown in Figure S45. The MOF efficiently absorbs in the visible part of the electromagnetic spectrum and shows phosphorescence in the red to far red part of the electromagnetic spectrum. Compared to the dissolved indicator, the emission spectrum is shifted bathochromically and the intensity of the $Q_x(0,1)$ band in the MOF is higher than for the $Q_x(0,0)$ band. As expected, the luminescence is quenchable by oxygen. Despite the long luminescence lifetime of nearly 40 µs, only ~ 11-fold quenching of the luminescence at 21 kPa oxygen in the gas phase compared to nitrogen was recorded, which is significantly less than the SURMOFs.

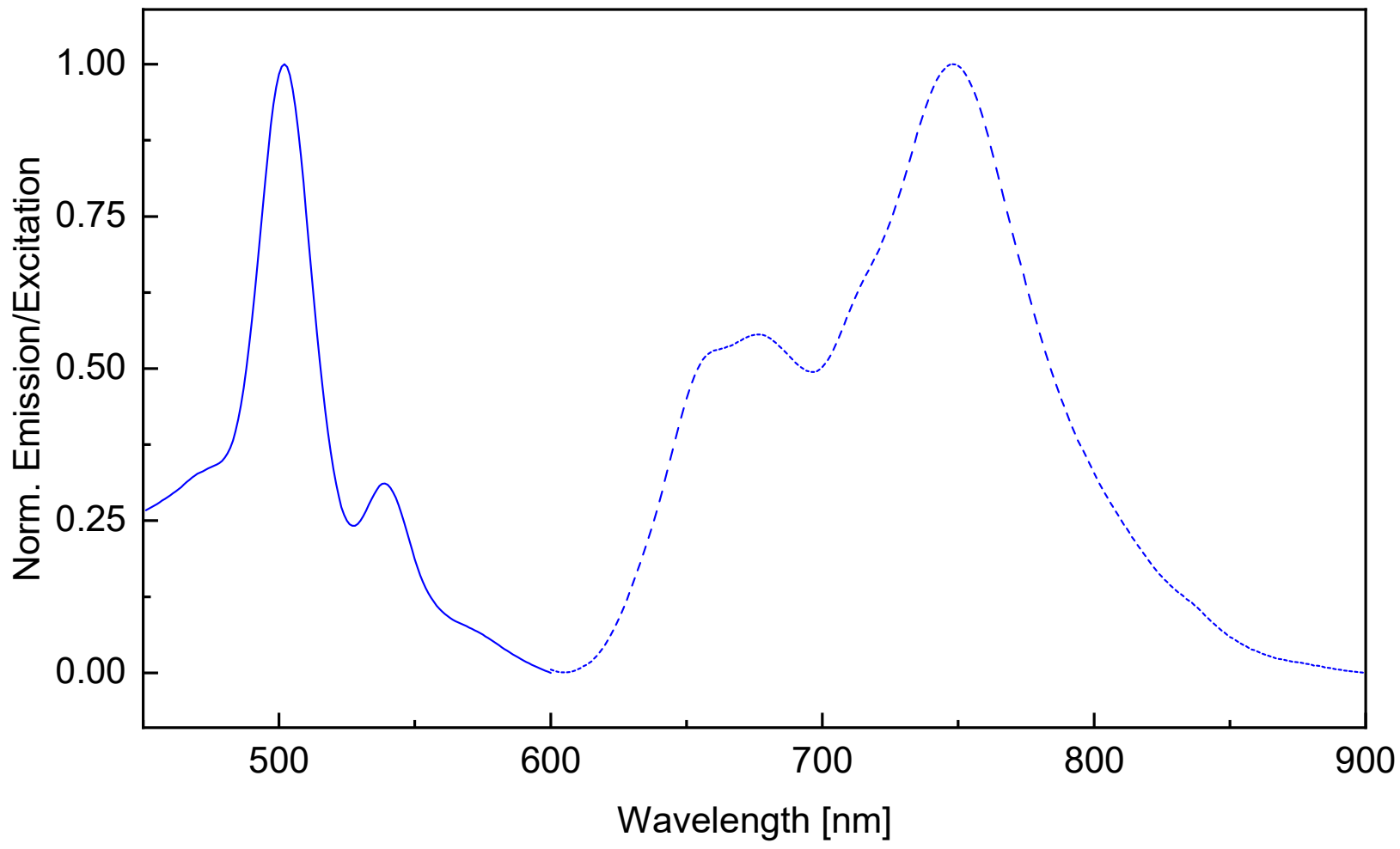

Figure S46: Normalized excitation (solid line; λ_{em} =650 nm) and emission spectra (dashed line; λ_{exc} =400 nm) of In-Pt(II)TCPP powder MOF acquired under anoxic conditions at room temperature.

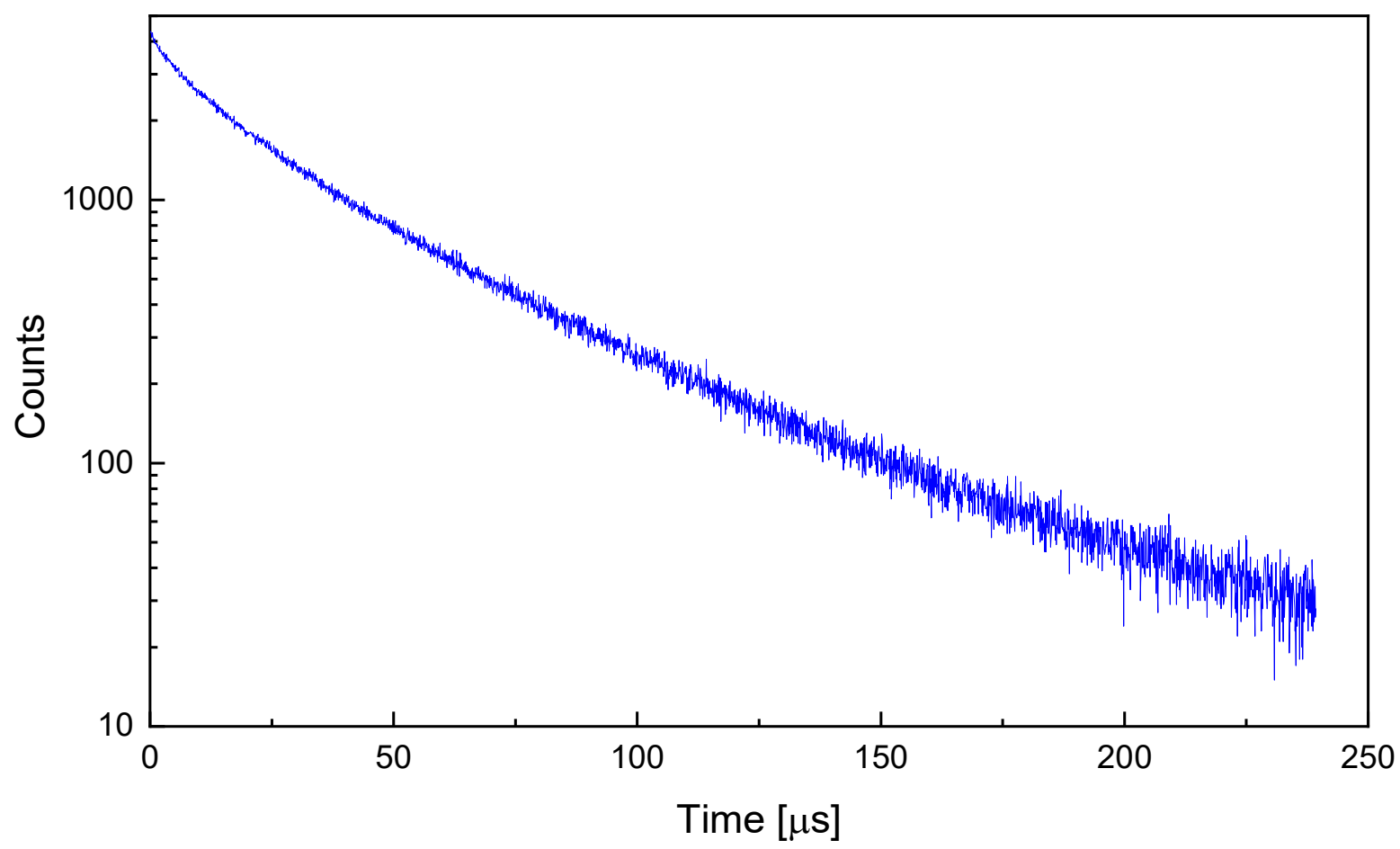

Figure S47: Luminescence decays of In-Pt(II)TCPP powder MOF at room temperature at anoxic conditions. The decay was recorded at the emission maxima of the MOF.

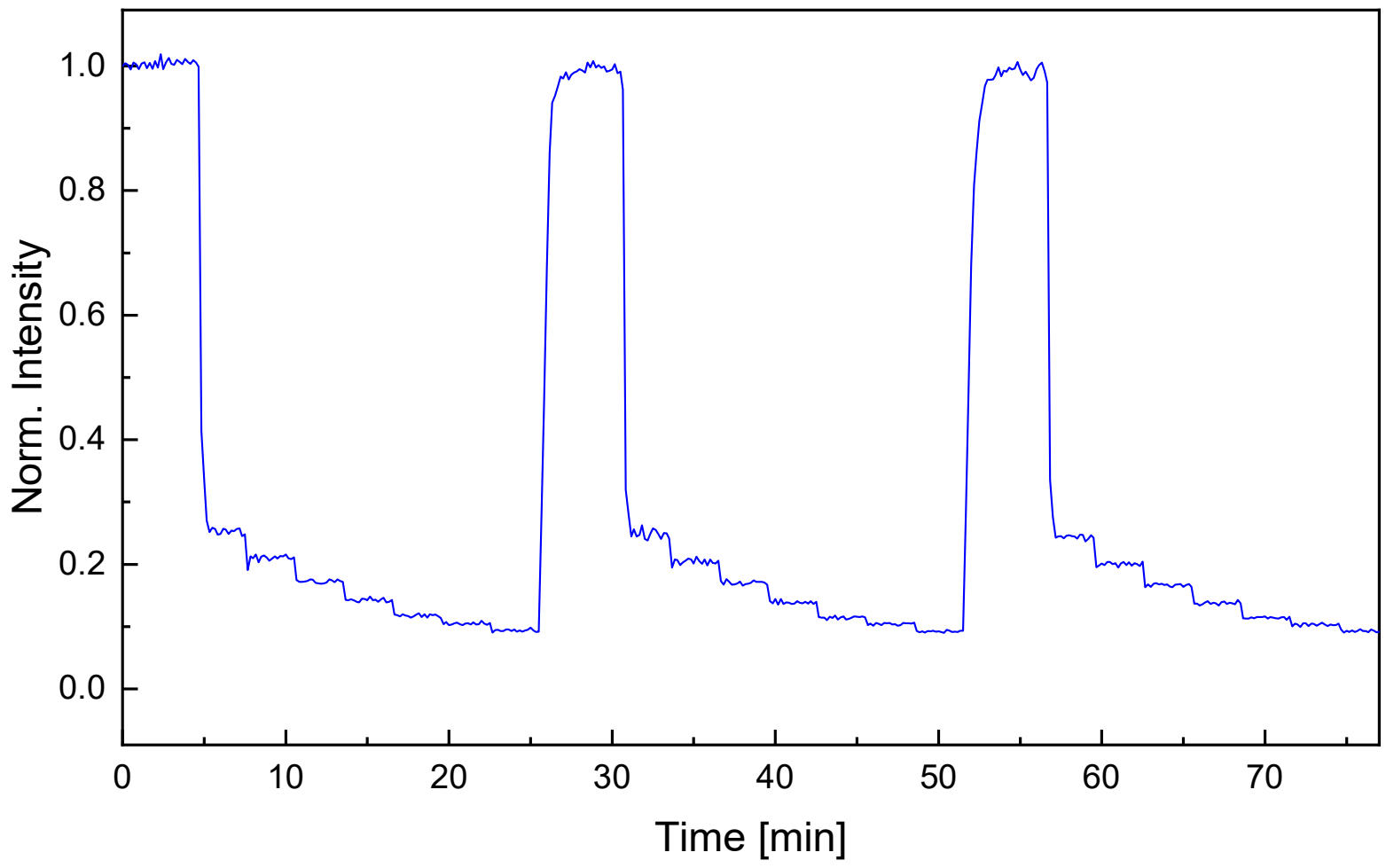

Figure S48: Luminescence response of In-Pt(II)TCPP powder MOF to varying oxygen partial pressure in gas. Intensity was monitored at the emission maxima (λ_{ex} = 400 nm).

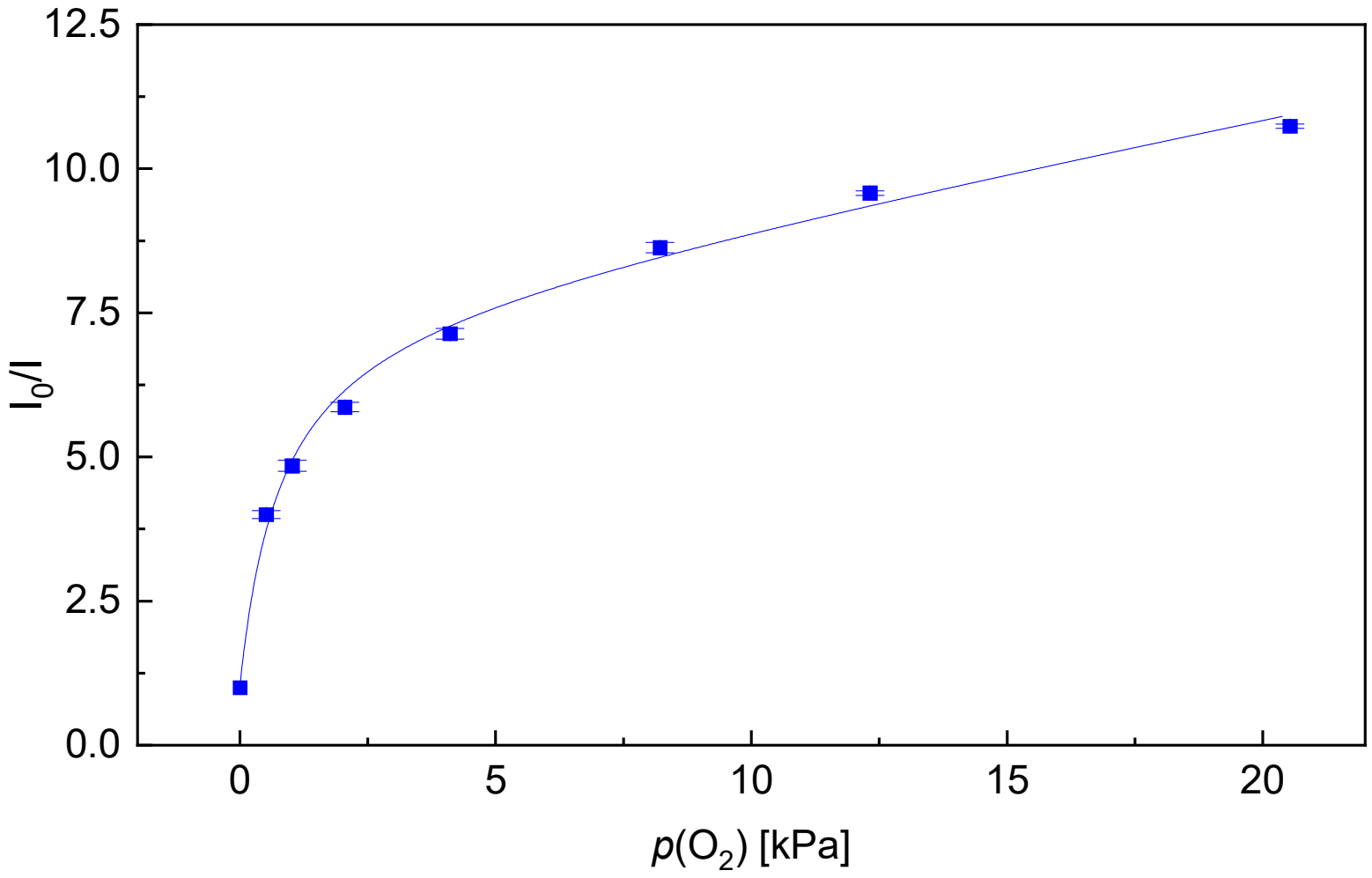

Figure S49: Stern-Volmer plot of In-Pt(II)TCPP powder MOF obtained by monitoring the maximum of the emission spectrum (λ_{ex} = 400 nm).

Table S3: Oxygen sensing properties of In-Pt(II)TCPP MOF. [a] acquired under anoxic conditions.

	**λ_{max} Abs, Q [nm]**	**λ_{max} Em[a] [nm]**	**I_0/I ~ 20.9 kPa**	**K_{SV1} [kPa^{-1}]***	**f**	**τ_0 [µs]**
In-Pt(II)TCPP MOF	502, 588	675, 748	10.7 ± 0.1	10.0	0.87	38.8

*K_{SV2} = 0.00467 kPa^{-1}

S5.3 Thermogravimetric analysis of In-Pt(II)TCPP powder MOF

The thermal stability of In-Pt(II)TCPP based powder MOF was investigated using thermalgravimetric analysis (TGA).

The sample shows two major mass losses during the measurement, at 200 °C to 250 °C and 350°C to 550 °C. Similar mass losses are obtained during TGA of In-TCPP powder MOF.[3] Mass loss below 250°C can be attributed to the loss of entrapped solvents (mainly DMF) in the pores. Following, the framework starts to collapse at 350°C.

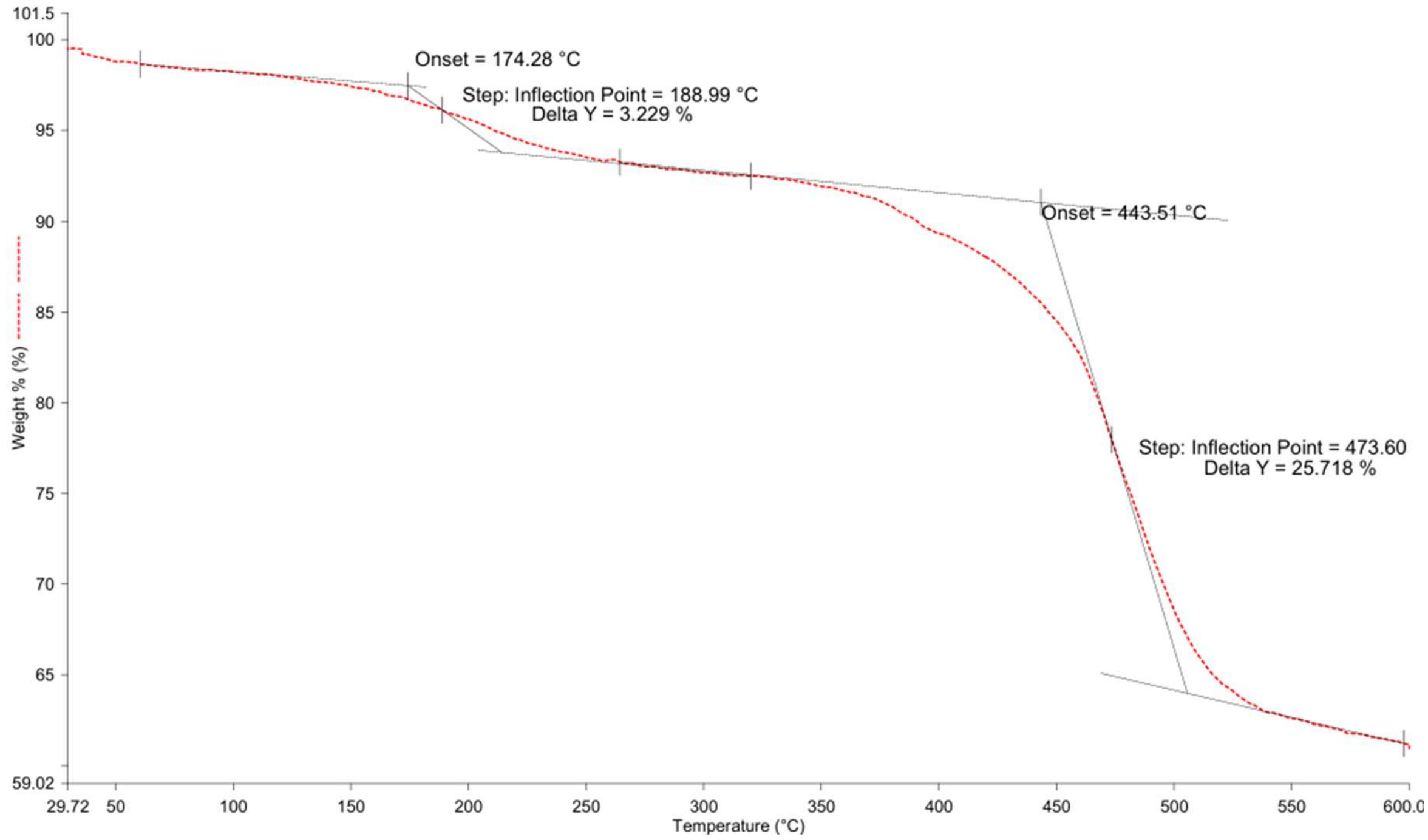

Figure S50: Thermogravimetric analysis of In-Pt(II)TCPP powder MOF samples under nitrogen atmosphere (continuous gas flow of 20 mL min^{-1}). Heating: 10 °C min^{-1}; the temperature was hold for 1 min at 30 °C and 600 °C.

S6. Overview of reported porphyrinic SURMOFs

Table S4: Summary of various porphyrin based SURMOFs. LPE liquid-phase epitaxial

Name	Organic linker	Organic linker conc.	Metal node	Metal node conc.	Solvent	Cycle time	Cyles	Substrate	Method	Reference
In-TCPP SURMOF	TCPP	0.1 mM	$In(NO_3)_3$	1 mM	Ethanol	30 min TCPP / 20 min $In(NO_3)_3$	5-20	OH-functionalized SiO_2/Si substrates	LPE*	[1]
In-TCPP thin film	TCPP	0.1 mM	$In(NO_3)_3$	1 mM	Ethanol	30 min TCPP / 20 min $In(NO_3)_3$	200	ITO glass and Si/SiO_2 substrates	LPE*	[4]
021-InTCPP(H_2)	TCPP	1 mM	$In(NO_3)_3$	10 mM	Ethanol	20 min	30	functionalized quartz substrate	LPE*	[5]
011-InTCPP(H2)	TCPP	0.1 mM	$In(NO_3)_3$	1 mM	Ethanol	20 min	30	functionalized quartz substrate	LPE*	[5]
Co-TCPP SURMOF	TCPP	0.2 mM	Co(II)acetate	1 mM	Ethanol	25 s	60	hydroxyl functionalized quartz substrates	spin coating	[6–8]
Zn TCPP thin film	TCPP	0.1 mM	Zinc acetate	1 mM	Ethanol	15 min TCPP/ 10 min Zinc acetate	5	OH functionalized FTO substrate	LPE*	[9,10]
Zn-TCPP-Pt thin film [(1)]	TCPP	0.1 mM	Zinc acetate	1 mM	Ethanol	15 min TCPP/ 10 min Zinc acetate	5	OH functionalized FTO substrate	LPE*	[9]
ZnTCPP(M) films	MTCPP (M = Zn, Mn, Fe)	0.05	Zinc acetate	1 mM	Ethanol	25 s MTCPP/ 15 s $Zn(OAc)_2$	20	OH-functionalized SiO_2/Si substrates	Spray method	[11]
SURMOF-DMF-38x	Pt(II)TCPP	38 µM	$InCl_3$	1.1 mM	DMF	20 min each	~ 85	COOH-functionalized glass/flexible PET	LPE*	this work

SURMOF-EtOH-38X	Pt(II)TCPP	38 µM	$InCl_3$	1.1 mM	Ethanol	20 min each	~ 85	COOH-functionalized glass	LPE*	this work

(1) Pt doped Zn-TCPP thin film obtained by post synthesis modification using K_2PtCl_4

S7. Overview of porous materials for optical sensing of oxygen

Table S5: Summary of (porphyrin-based) metal-organic frameworks for optical sensing of oxygen.

Material		Luminescent species	τ_0	K_{SV1}	I_0/I	Reference
EuNDC-MOF	Gas phase	Eu(III) bridged with 1,4-naphthalenedicarboxylic acid	1.3 ms	0.13 kPa^{-1}	15.4 @ 101 kPa	[12]
Pyrene@MAF-4	Gas phase	Pyrene	58,7 ns	0.061 kPa^{-1}	~ 7 @ 100 kPa	[13]
M66:MAF-2	Gas phase	phosphorescent copper(I) triazolate framework matrix	101 µs	1480 kPa^{-1}	~ 1.7 @ 4.5 ppm O_2	[14]
R-UiO	HBSS buffer	Pt-5,15-di(p-benzoato)porphyrin	28 µs	0.13 kPa^{-1}	-	[15]
Pt(II)PCN-224	Gas phase	Pt(II)TCPP	19 µs	73 kPa^{-1}	~ 5 @ 0.2 kPa	[16]
PCN-224	Gas phase	TCPP	6,9 ns	0.39 kPa^{-1}	6.9 @ 20 kPa	
PCN-223	Gas phase	TCPP	3,1 ns	0.16 kPa^{-1}	4.1 @ 20 kPa	[17]
PCN-222	Gas phase	TCPP	2,4 ns	0.10 kPa^{-1}	2.6 @ 20 kPa	
Pt(II)PCN-224	Aqueous solution	Pt(II)TCPP	24 µs	0.0113 μM^{-1}	~ 3 @ air saturation	[18]
SURMOF-DMF-38**	Gas phase	Pt(II)TCPP	42 µs	11 kPa^{-1} *	57 @ 20.9 kPa	
SURMOF-EtOH-38**	Gas phase	Pt(II)TCPP	40 µs	12 kPa^{-1} *	64 @ 20.9 kPa	this work
SURMOF-DMF-38	Toluene	Pt(II)TCPP	-	0.18 kPa^{-1} *	2.4 @ 20.9 kPa	

* K_{SV2} was set to 0
** the most sensitive sample of the series is listed here

S8. References

1 Y. Tian, N. Vankova, P. Weidler, A. Kuc, T. Heine, C. Wöll, Z. Gu and J. Zhang, *Adv. Sci.*, 2021, **8**, 2100548.
2 T. K. Mautz, G. Schwendt, M. D. J. Velasquez-Hernandez, F. Warchomicka, C. Slugovc and S. M. Borisov, *ACS Appl. Opt. Mater.*, 2025, **3**, 2271–2282.
3 F. Leng, H. Liu, M. Ding, Q.-P. Lin and H.-L. Jiang, *ACS Catal.*, 2018, **8**, 4583–4590.
4 X. Yang, J. Huang, J. Li, Y. Zhao, H. Li, Z. Yu, S. Gao and R. Cao, *Adv. Mater.*, 2024, **36**, 2313608.
5 Y.-B. Tian, Q.-H. Li, Z. Wang, Z.-G. Gu and J. Zhang, *Nano Lett.*, 2023, **23**, 3062–3069.
6 C. Gu, H. Zhang, J. Yu, Q. Shen, G. Luo, X. Chen, P. Xue, Z. Wang and J. Hu, *Nano Lett.*, 2021, **21**, 1102–1107.
7 C. Gu, H. Zhang, P. You, Q. Zhang, G. Luo, Q. Shen, Z. Wang and J. Hu, *Nano Lett.*, 2019, **19**, 9095–9101.
8 J. Yu, Y. Han, Y. Yang, H. Zhang, S. Fang, Z. Wang and J. Hu, *Adv. Opt. Mater.*, 2024, **12**, 2400285.
9 Y.-B. Tian, Y.-Y. Wang, S.-M. Chen, Z.-G. Gu and J. Zhang, *ACS Appl. Mater. Interfaces*, 2020, **12**, 1078–1083.
10 Y. Wang, S. Chen, R. Haldar, C. Wöll, Z. Gu and J. Zhang, *Adv. Mater. Interfaces*, 2018, **5**, 1800985.
11 D.-J. Li, Y.-B. Tian, Q. Lin, J. Zhang and Z.-G. Gu, *ACS Appl. Mater. Interfaces*, 2022, **14**, 33548–33554.
12 T. Xia, L. Jiang, J. Zhang, Y. Wan, Y. Yang, J. Gan, Y. Cui, Z. Yang and G. Qian, *Micropor. Mesopor. Mat.*, 2020, **305**, 110396.
13 J.-W. Ye, H.-L. Zhou, S.-Y. Liu, X.-N. Cheng, R.-B. Lin, X.-L. Qi, J.-P. Zhang and X.-M. Chen, *Chem. Mater.*, 2015, **27**, 8255–8260.
14 S.-Y. Liu, D.-D. Zhou, C.-T. He, P. Liao, X.-N. Cheng, Y.-T. Xu, J.-W. Ye, J.-P. Zhang and X. Chen, *Angew. Chem. Int. Ed.*, 2016, **55**, 16021–16025.
15 R. Xu, Y. Wang, X. Duan, K. Lu, D. Micheroni, A. Hu and W. Lin, *J. Am. Chem. Soc.*, 2016, **138**, 2158–2161.
16 T. Burger, C. Winkler, I. Dalfen, C. Slugovc and S. M. Borisov, *J. Mater. Chem. C*, 2021, **9**, 17099–17112.
17 T. Burger, M. V. Hernández, C. Carbonell, J. Rattenberger, H. Wiltsche, P. Falcaro, C. Slugovc and S. M. Borisov, *ACS Appl. Nano Mater.*, 2023, **6**, 248–260.
18 J. Yang, Z. Wang, Y. Li, Q. Zhuang and J. Gu, *Chem. Mater.*, 2016, **28**, 2652–2658.
